# Evidence for conserved expression of genes annotated as associated with brain-related biological processes in human podocytes and brain

**DOI:** 10.1186/s12882-026-04877-2

**Published:** 2026-03-04

**Authors:** Wasco Wruck, Chantelle Thimm, Abida Islam Pranty-Weßelmann, James Adjaye

**Affiliations:** 1 Institute for Stem Cell Research and Regenerative Medicine, Medical Faculty, Heinrich Heine University, Moorenstr.5, 40225 Düsseldorf, Germany; 2 Centre for Nephrology, Urology and Transplantation, King’s College London, Guy’s Campus, Great Maze Pond, London, SE1 1UL UK

**Keywords:** Podocytes, Brain, Neurons, Microtubules, Actin cytoskeleton, Process formation, Renal glomerulus, Synaptic signaling, Foot processes

## Abstract

**Background:**

Similarities between podocytes and brain reside in biological processes related to actin-based projections such as podocyte foot processes, axons and dendritic spines. Further similarities are associated with cell-cell contacts in synapses and the slit diaphragm where podocalyxin, neurexin, cadherins/protocadherins and Ig-like proteins are crucial for these adhesion processes. Additionally, podocytes employ signaling mechanisms involving neurotransmitters as found in brain synapses such as the glutamate receptors.

**Methods:**

In this study, we analysed brain-associated biological processes, transcription factors and pathways significantly regulated in UdPodocytes, i.e. podocytes differentiated from SIX2-positive UdRPCs (urine-derived renal progenitor cells). We compared gene expression in iPSC-derived brain and kidney organoids with UdPodocytes and mapped the overlapping 344 genes to brain regions via the GTEX (Genotype-Tissue Expression) database and investigated their regulation induced by the mediator of the renin-angiotensin system - angiotensin II (ANG II).

**Results:**

The protein interaction network of UdPodocytes genes associated with brain in GTEX contains modules for pre-synapse, post-synapse, endocrine processes and neural crest differentiation. We also found that the genes overlapping between brain and podocytes are also expressed in iPSC-derived kidney and brain organoids and could map the involved genes to all regions of the brain with the frontal cortex as the most enriched. The genes SUZ*12*,* NFKB1* and *PAX2* were the most significantly over-represented transcription factors. We independently confirmed the conserved expression of *PAX6*,* KCNQ3* and *TUBB3* mRNA in UdPodocytes, biopsy-derived kidney and brain samples. MAP2, TAU and TUBB3 expression is shown by Western blotting and Immunofluorescence analysis. We further investigated if the conserved genes are also regulated by ANGII. This unveiled genes down-regulated upon ANGII-stimulation of Udpodocytes to be associated with axon guidance, Calcium, Hippo and cGMP-PKG signaling as over-represented pathways.

**Conclusion:**

In conclusion, we have identified 344 genes with overlapping expression in brain and podocytes. These are mainly associated with synaptic signaling and cell projections. This implies that human urine-derived SIX2-renal progenitor cells differentiated into podocytes can serve as a platform for dissecting and understanding the relevance of conserved biological processes in podocytes which are currently annotated as neuron projection, axons, neurogenesis and synaptic signaling.

**Supplementary Information:**

The online version contains supplementary material available at 10.1186/s12882-026-04877-2.

## Introduction

The central nervous system (CNS) and the kidneys are very closely connected, for example, the CNS provides afferent impulses that regulate and influence renal blood flow, the glomerular filtration rate (GFR) and the sodium balance [1] and Cao et al. report a kidney-brain neural circuit driving kidney damage and heart failure [2]. With respect to early development, podocytes and neurons are derived from distinct lineages, neurons arise from the ectodermal germ layer and podocytes from metanephric mesenchyme [3] developing from the intermediate part of the mesoderm germ layer [4].

The similarity between podocytes and neurons is largely determined by common features of primary processes operative in both cell types as well as the need to exchange signals across membranes in synaptic or synaptic-like constellations [5]. Processing of formation in podocytes and neurons use the same molecular machinery which include actin-based projections such as podocyte foot processes and dendritic spines [6]. These are organized in microtubules (MT) and intermediate filaments for thick processes and actin filaments for thin projections [6]. Further similarity resides in the cell-cell contacts in synapses and the slit diaphragm where adhesion molecules in neurons and podocytes belong to the same families such as Cadherins/Protocadherins, Ig-like proteins, Neurexin and Podocalyxin [7].

Synaptopodin (SYNPO) is an Actin-associated protein that regulates the cytoskeleton in podocytes. It is not only expressed in mature podocytes but also in parts of the cytoskeleton of postsynaptic densities (PSD) and their dendritic spines. Studies have shown that the expression of SYNPO is restricted to a sub-population of cells within the telencephalic synapse [8, 9].

Furthermore, communication between podocytes and other glomerular cells is mediated via signaling mechanisms involving neurotransmitters which are also found in synapses [7] such as the glutamate receptors NMDAR and GRM1. Within podocytes, Nephrin connects these glutamate receptors through scaffolding molecules, such as PSD95, to the actin cytoskeleton of foot processes [7].

Numerous kidney-associated diseases can result in neuronal damage. A decrease in the glomerular filtration rate (GFR) due to age or disease leads to higher concentration of the filtered waste products in the blood and can cause uremia. This accumulation of uremic substances in the blood can exert toxic effects on neurons such as in uremic encephalopathy, uremic neuropathy and uremic myopathy [10].

Besides the uremic toxins, oxidative stress, inflammation, and impaired blood circulation and blood brain barrier are factors mediating neurological disorders in kidney diseases, particularly in chronic kidney disease (CKD). On the other hand, leakage in the glomerular filtration barrier which leads to low concentrations of molecules such as Albumin in the blood and high concentrations in the urine are another source of kidney damage such as in diabetic nephropathy.

The similarity between podocytes and neurons manifests directly in diseases associated with calcium signaling. Deregulated or non-functional proteins from the TRPC family are associated with focal segmental glomerulosclerosis (FSGS- TRPC6) and TRCP5 induces pathologically motile podocytes while in brain the loss of TRCP5 can result in deficits in gait and motor coordination [7]. Another disease affecting neurons and podocytes, Charcot-Marie-Tooth neuropathy, often co-occurring with FSGS, is associated with mutations in *INF2* which is involved in regulation of the actin and microtubule cytoskeleton [11]. Interestingly, apart from podocytes, brain dysfunction in tubular and tubulointerstitial diseases is more often seen in disorders of water handling such as Bartter and Gitelman syndromes [12]. However, there are tubulointerstitial diseases with neuronal effects such as Nephronophthisis, a nephropathy with comorbidities such as retinal degeneration (Senior–Løken syndrome) and cerebellar vermis aplasia (Joubert syndrome)] [13].

In this study, we further investigated similarities between a set of genes expressed in brain and podocytes with respect to neuronal-associated gene ontologies operative in urine-derived podocytes [14]. We also confirmed expression in iPSC-derived brain and kidney organoid cultures and revealed their responsiveness to ANGII-stimulation. Signalling pathways and gene regulatory networks suggest common functionality in brain and podoytes.

## Methods

### Cell culture conditions

#### Urine-derived renal progenitor cells cultivation and differentiation into podocytes and tubular cells

Urine-derived renal progenitor cells (UdRPCs) from distinct individual males with ages 48 and 51 years (UM48, UM51) and 21 and 27 year old females (UF21, UF27), were isolated following the protocol detailed by Rahman et al. [15]. These cell cultures were maintained on 6- or 12-well plates uncoated at 37 °C in a hypoxic environment. Cells were grown in Proliferation Medium (PM), consisting of a 1:1 mixture of DMEM high-glucose (Gibco) and keratinocyte growth basal medium (Lonza, Basel, Switzerland), supplemented with 5% fetal bovine serum (Gibco), 0.5% non-essential amino acids (Gibco), 0.25% Glutamax (Gibco), and 0.5% penicillin-streptomycin (Gibco).

For differentiation into podocytes, cells were seeded at a low density (50,000 cells per 6-well plate) on a Corning^®^ Collagen I (Merck, Darmstadt, Deutschland) coated six or 12-Well plate and cultured for 24 h in PM. The following day, the medium was replaced with Advanced RPMI 1640 (Gibco) supplemented with 0.5% fetal bovine serum, 1% penicillin-streptomycin, and 30 µM retinoic acid (Sigma-Aldrich Chemistry, Steinheim, Germany). After seven days, cells exhibited the characteristic morphology of podocytes. The cells were cultured for 14 days under these conditions. Furthermore, a human SV40-temperature sensitive immortalized podocyte cell line (AB 8/13) [16] was used as an established reference. The cells were first cultured at 33 °C in RPMI 1640 (Gibco). To induce podocyte differentiation and maturation the, cells were seeded on Corning^®^ Collagen I (Merck, Darmstadt, Deutschland) coated dishes and cultured at 37 °C in Advanced RPMI 1640 (Gibco) supplemented with 0.5% fetal bovine serum, 1% penicillin-streptomycin, and 30 µM retinoic acid (Sigma-Aldrich Chemistry, Steinheim, Germany). Both the urine-derived podocytes and the human immortalized cell line (AB 8/13) were cultured for 14 days at 37 °C under identical conditions.

Tubular cell differentiation was initiated after culturing undifferentiated UdRPC on laminin-421-coated plates to a confluency of approximately 90% (laminin-421, #LN421-02, BioLamina, Sundbyberg, Sweden). Differentiation was induced in renal epithelial cell growth medium (REGM) supplemented with 10 ng/ml bone morphogenetic protein 2 (BMP2) and 2.5 ng/ml BMP7 for seven days.

#### Generation of neural progenitor cells (NPCs) and further differentiation into neuronal networks

A healthy male-derived iPSC line (UM51-iPSC) was used in this study. The UM51-iPSC line was generated from SIX2-positive renal progenitor cells isolated from the urine of a 51-year-old healthy male of African origin (UM51) [17, 18]. iPSCs were cultured on Matrigel-coated (Corning, NY, USA) tissue culture plates in StemMACS (Miltenyi, Bergisch Gladbach, Germany). Cells were passaged every 5–7 days using DPBS (Thermo Fisher Scientific) to dissociate colonies into small aggregates and reseeded at a 1:6 split ratio onto fresh Matrigel-coated plates.

For the induction of neural progenitor cells (NPCs) from iPSCs we employed our previously published protocol- Pranty et al. [19]. iPSCs were dissociated into single cells using accutase (Life Technologies, Waltham, MA, USA). Thereafter they were centrifuged at 200 × g for 5 min, and resuspended in StemMACS supplemented with 10 µM ROCK inhibitor. A total of 20,000 cells in 100 µL suspension were seeded per well of a low-attachment 96-well U-bottom plate to generate embryoid bodies (EBs). Plates were centrifuged at 110 × g for 3 min and incubated at 37 °C in 5% CO₂ for 24 h. On the next day, half of the medium volume was aspirated and replaced with neural induction medium (NIM) with 10µM of ROCK inhibitor. From day three until day seven the EBs were cultured with NIM supplemented with 10 µM SB431542 and 5 µM Dorsomorphin. Medium was refreshed daily. On day 8, 20 to 30 EBs were transferred to a Poly-Ornithine/Laminin (Sigma-Aldrich; Merck KGaA, Darmstadt, Germany) -coated 6-well plate to promote the formation of neural rosettes. From this point onwards the rosettes were cultured in neural differentiation medium (NDM) (Neurobasal A, 1% B27, 1% GlutaMAX, 1% P/S) supplemented with 20 ng/mL EGF and 20 ng/mL FGF2. The incubation took place under static conditions at 37 °C with 5% CO2 with daily medium changes. On day 16, neural rosettes were isolated using STEMdiff™ Neural Rosette Selection Reagent (StemCell Technologies, Vancouver, Canada) for 30 min–1 h at 37°. Rosettes were subsequently dissociated with accutase for 30 min at 37 °C. NPCs were expanded on growth factor-reduced (GFR) Matrigel-coated plates in NDM supplemented with 20 ng/mL EGF and 20 ng/mL FGF2. The expansion and splitting was performed with accutase.

For further differentiation into neuronal cells NPCs were dissociated with accutase and plated onto GFR Matrigel-coated plates. For this, cells were seeded at a density of 500,000 cells per well in 6-well plates and approximately 80,000 cells per well in 24-well plates. For initial cell attachment, cultures were maintained for 24 h in NDM supplemented with 20 ng/mL EGF and 20 ng/mL FGF2. Thereafter (day 2–16), the medium was replaced with NDM supplemented with 20 ng/mL brain-derived neurotrophic factor (BDNF) and 20 ng/mL neurotrophin-3 (NT-3). Cells were maintained until day 16, with medium changes every 2–3 days. Neuronal cultures were harvested on day 19.

#### Formation of neuronal cortical organoids

For the generation of 3D neural cortical organoids, we again employed our previously published protocol described by Pranty et al. [20].For differentiation, approximately 20,000 single iPSCs were seeded per well of a U-bottom 96-well plate (Nucleon™ Sphera™, Thermo Fisher Scientific) in mTeSR Plus medium supplemented with 10 µM ROCK inhibitor Y-27,632 to generate embryoid bodies (EBs). EBs were maintained for five days in neural induction medium (StemCell Technologies) to initiate neural differentiation. On day six, EBs were transferred to a bioreactor (PFEIFFER) containing differentiation medium composed of DMEM/F12 and Neural Basal Medium (1:1), supplemented with N2 (1:200), L-glutamine (1:100), B27 without vitamin A (1:100), penicillin (100 U/mL), streptomycin (100 mg/mL), MEM non-essential amino acids (0.05 mM), β-mercaptoethanol (0.05 mM) (all Gibco), and insulin (23 µM; Sigma). Spinner flasks were pre-treated with an anti-adherent rinsing solution for 10 min at 37 °C before EB transfer. From this time point (differentiation day six), transferred EBs were defined as day-0 organoids, as spontaneous cortical patterning occurred in suspension culture. From day nine onward, dorsomorphin (0.5 µM) and SB431542 (5 µM) were added to the differentiation medium, which was replaced weekly. On Day 20 the cortical organoids were harvested.

#### Immunofluorescence-based detection of protein expression

Cells were fixed with 4% paraformaldehyde for 15 min at room temperature (RT) and subsequently washed three times with PBS. Afterwards, the cells were blocked using 3% BSA in PBS for 2 h at RT on a shaking platform. Primary antibodies (detailed in Table S5) were applied, and the cells were incubated overnight at 4 °C. The next day, the cells underwent three washing steps with PBS. Secondary antibodies (detailed in Table S5) together with Hoechst as a nuclear stain were applied for 1 h at RT. Fluorescence imaging was performed using an LSM700 microscope (Carl Zeiss). For optimal visualisation, 400ms exposure was applied to both 10x and 20x magnifications.

### Western blotting

Cells were lysed using RIPA buffer (Sigma-Aldrich Chemistry) supplemented with 5 M NaCl, 1% NP-40, 0.5% DOC, 0.1% SDS, 1 mM EDTA, and 50 mM Tris (pH 8.0), along with freshly added protease and phosphatase inhibitors (10 µL/mL, Sigma-Aldrich). A total of 20 µg of protein from each lysate was resolved on a 10% SDS-PAGE gel and transferred onto an Immobilon-P membrane (Merck Millipore, Burlington, VT, USA). Membranes were incubated overnight at 4 °C with the primary antibody, followed by three washes with 0.1% Tween-20 in Tris-buffered saline. Secondary antibodies were then applied for 1 h at room temperature. Protein visualisation was performed using the Pierce™ ECL Western Blotting Substrate (Thermo Fisher Scientific, Massachusetts, USA), with both solutions mixed at a 1:1 ratio. Quantification of protein bands was conducted using ImageJ software. Further details regarding the antibodies used are available in Supplemental Table S5.

### RNA isolation

The ZYMO Research Kit Direct-zol™ RNA Miniprep R20 was utilized for isolating RNA. In brief, cells were detached by incubating with Tryple E for approximately 5 min. Thereafter detached cells were pelleted by centrifugation for 4 min at 1000 g prior to RNA isolation. RNA samples were eluted by adding 35 µL of DNase/RNase-free water to the column and centrifuging for 1 min at 16,000 g. The eluted RNA samples were immediately placed on ice, and concentrations measured with a Nanodrop device.

### Semi-quantitative RT- PCR

1000 ng of RNA sample was used for reverse transcription using the TaqMan Kit following the manufacturer’s instructions. PCR was performed using the GoTaq^®^ G2 Hot Start Polymerase Kit from Promega. For each sample, 1 µL cDNA (5 ng/µL) was mixed with 24 µL master mix. As a negative control, 1 µL RNAse-free water was used. As references, commercial RNA samples of Human kidney total RNA (Takara, #636529) and fetal brain (BioChain^®^) were used. PCR was performed using a thermal cycler under the following conditions, polymerase activation: 95 °C for 2 min; 35 cycles amplification: 95 °C for 30 s, 60 °C for 30 s, 72 °C for 30 s; final elongation: 72 °C for 5 min, and finally held at 4 °C. The PCR product was resolved in a 2% agarose gel by gel electrophoresis. *RPL0* was used as the housekeeping gene. The imaging device Fusion FX was used for visualization.

### Acquisition of brain and podocyte transcriptome data

Microarray datasets related to experiments conducted with brain cells and podocytes were downloaded from the public repository at the NCBI GEO (National Center for Biotechnology Information, Gene Expression Omnibus) (Table 1). In order to minimize technical variability only datasets from the same technical platform were employed. Kidney-related datasets are associated with two previous studies from our lab by Erichsen et al. (GSE171240) pertaining to Angiotensin-II-treated podocytes [14] and by Nguyen et al. (GSE186823) containing kidney organoids treated with the nephrotoxin Puromycin Aminonucleoside (PAN) [21]. Brain-related datasets are associated with a previous study from our lab pertaining to brain organoids [22] and with datasets associated with genes expressed in brain regions based on RNAseq data from the Genotype-Tissue Expression (GTEx) project [23].

**Table 1:** Brain and kidney transcriptome datasets from NCBI GEO and other sources used in this study

dataset	contents	brain/kidney region	platform	PubMedId
GSE279611	control	brain organoids	Affymetrix Human Clariom-S	35269426
GTEX	tissue gene expression	brain regions	RNA-Seq	23715323
GSE171240	ANGII treated podocytes	podocytes	Affymetrix Human Clariom-S	35406662
GSE186823	PAN treated kidney organoids	kidney organoids	Affymetrix Human Clariom-S	35203286

### Identification of brain-related biological processes in urine-derived podocytes

This study was based on over-represented brain-associated GOs in transcriptomes of urine derived SIX2-positive renal progenitor cells -UdRPCs differentiated into podocytes and stimulated with ANGII [14]. In the gene expression data from our previous study by Rahman et al. [17] genes expressed in UdRPCs and UdPodocytes [14] were compared in a Venn diagram using the VennDiagram package [24] from the R/Bioconductor environment [25]. Gene expression was determined by a detection-p-value < 0.05 as described in our previous publication [26]. Genes exclusively expressed in the UdPodocytes but not in the UdRPCs were subjected to over-representation analysis via the GOstats package [27].

### Association of genes with whole brain and brain regions

Genes associated with tissues were downloaded from the GTEX portal (version GTEx_Analysis_v6_RNA-seq_RNA-SeQCv1.1.8_gene_median_rpkm.gct) [23] as median RPKM data from RNAseq experiments. From these genes with a median RPKM value five times greater than the mean of all tissues were considered specific for that tissue. Particularly for this study genes associated with brain regions were extracted from the dataset.

### Comparison of brain-related genes in urine-derived podocytes to kidney and brain organoids

Genes expressed in kidney as well as brain organoids were extracted from our previous studies based on iPSC-derived kidney [21] and brain organoids [22]. The gene-set expressed in common was compared to the genes we found above exclusively expressed in UdPodocytes in a Venn diagram via the R package VennDiagram [24]. The intersection set from the venn diagram was further refined in a pie-chart for brain regions associated with the contained genes in the GTEX dataset. The pie-chart of brain regions was drawn with the function “pie3D” from the R package “plotrix”. Analysis of transcription factors over-represented in the promoter regions of the intersection gene set was preformed via the EnrichR web tool [28]. Association of genes to GO terms were made via the package “org.Hs.eg.db” from the R/Bioconductor [25]. For determining transcription factors (TFs), the GO terms of category “Molecular Function” with the 20 most associated genes were extracted via ratios dividing the number of associated genes by the total gene number. TFs were taken from the GO terms nucleic acid binding, DNA binding, transcription regulator activity, sequence-specific DNA binding and DNA-binding transcription factor activity, RNA polymerase II-specific. The protein interaction network was constructed using these TFs and Biogrid interactions [29] as described in our previous publication [22].

### Comparison of neuron-related genes in urine-derived podocytes to brain-associated genes

Genes associated with whole brain, i.e. all brain regions, in the GTEX dataset were compared to the genes exclusively expressed in UdPodocytes in a Venn diagram via the R package VennDiagram [24]. The intersection gene set from the venn diagram was submitted to the STRING-DB web tool [30] to construct a protein interaction network which in turn was used as input for Cytoscape [31]. In Cytoscape a clustering for subnetworks was performed via the plugin MCODE [32]. The sub-networks were highlighted in the whole network and analyzed for enrichment via cytoscape-builtin functions. Most significant terms from the enrichment analysis were plotted as barcharts of negative log10 p-values indicating ratios of involved gene numbers to total gene numbers on a color scale via the R package ggplot2 [33].

## Results

### Urine-derived Podocytes develop intercellular projections

**Fig. 1:**
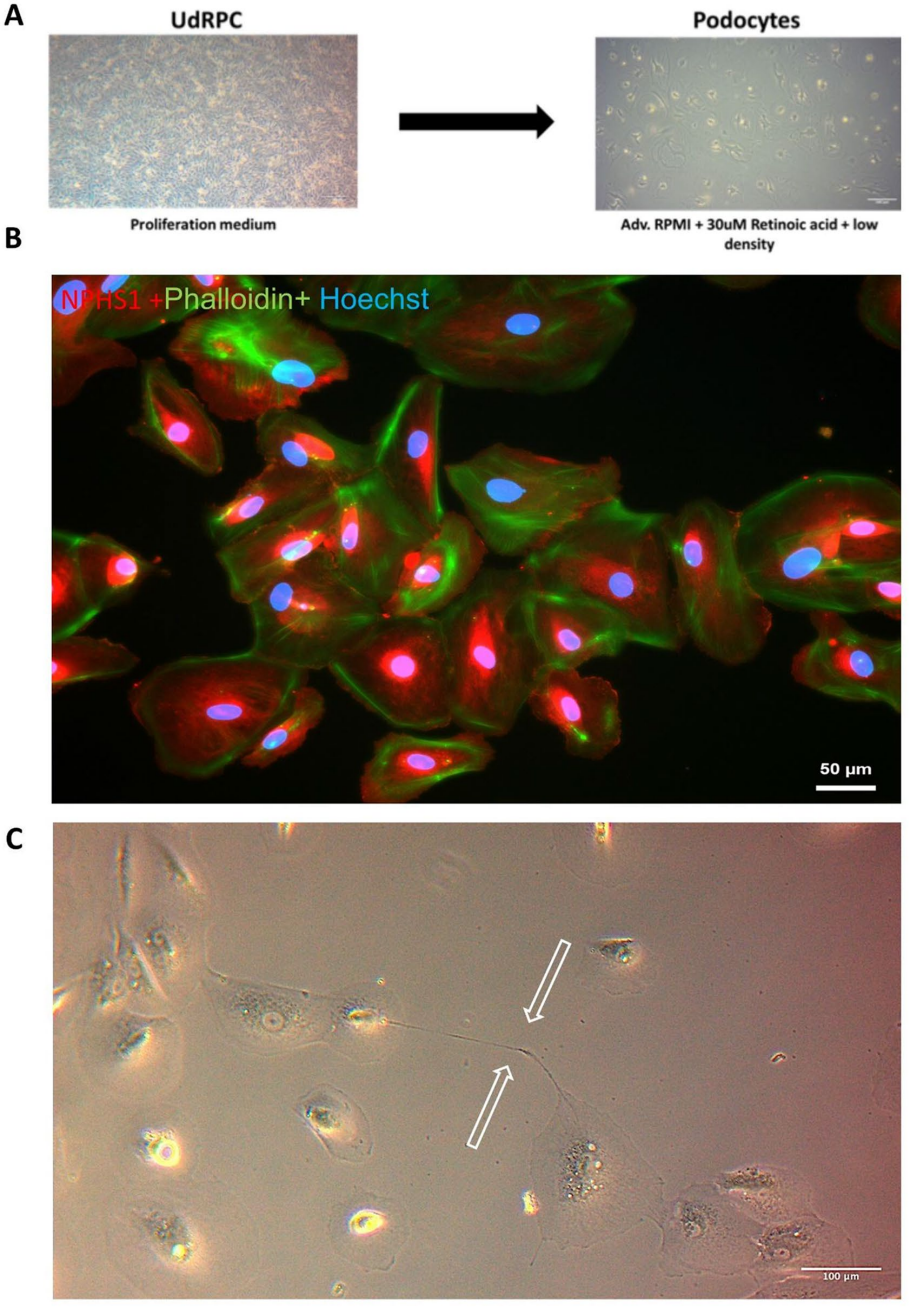
Podocytes develop projections with synapses. (**a**) Urine-derived renal progenitor (UdRPCs) were differentiated into UdPodocytes according to this scheme. (**b**) Nephrin (NPHS1) plays a major role in podocyte development and is found at the slit diaphragm. The staining here shows NPHS1 in red and phalloidin in green in UdPodocytes. (**c**) Podocytes derived from UdRPCs can develop intercellular projections inter-connecting other cells (arrows pointing at examples, more examples are shown in Figure S1)

Urine-derived renal progenitor cells (UdRPCs) were derived from urine and differentiated into podocytes (UdPodocytes) according to the scheme illustrated in Fig. 1a [14, 34]. Nephrin (NPHS1) plays a major role in podocyte development and is localised at the slit diaphragm (Fig. 1b: NPHS1 - red staining, phalloidin - green staining in UdPodocytes). NPHS1 is expressed in developing brain [35] and we found it up-regulated in kidney organoids [21]. The green phalloidin staining shows the actin filament structure typical for podocytes. UdPodocytes can sporadically develop intercellular projections connecting to other cells (Fig. 1c, white arrows pointing to intercellular projections, more examples are shown in Figure S1).

### Identification of brain-associated biological processes in urine-derived podocytes

**Fig. 2:**
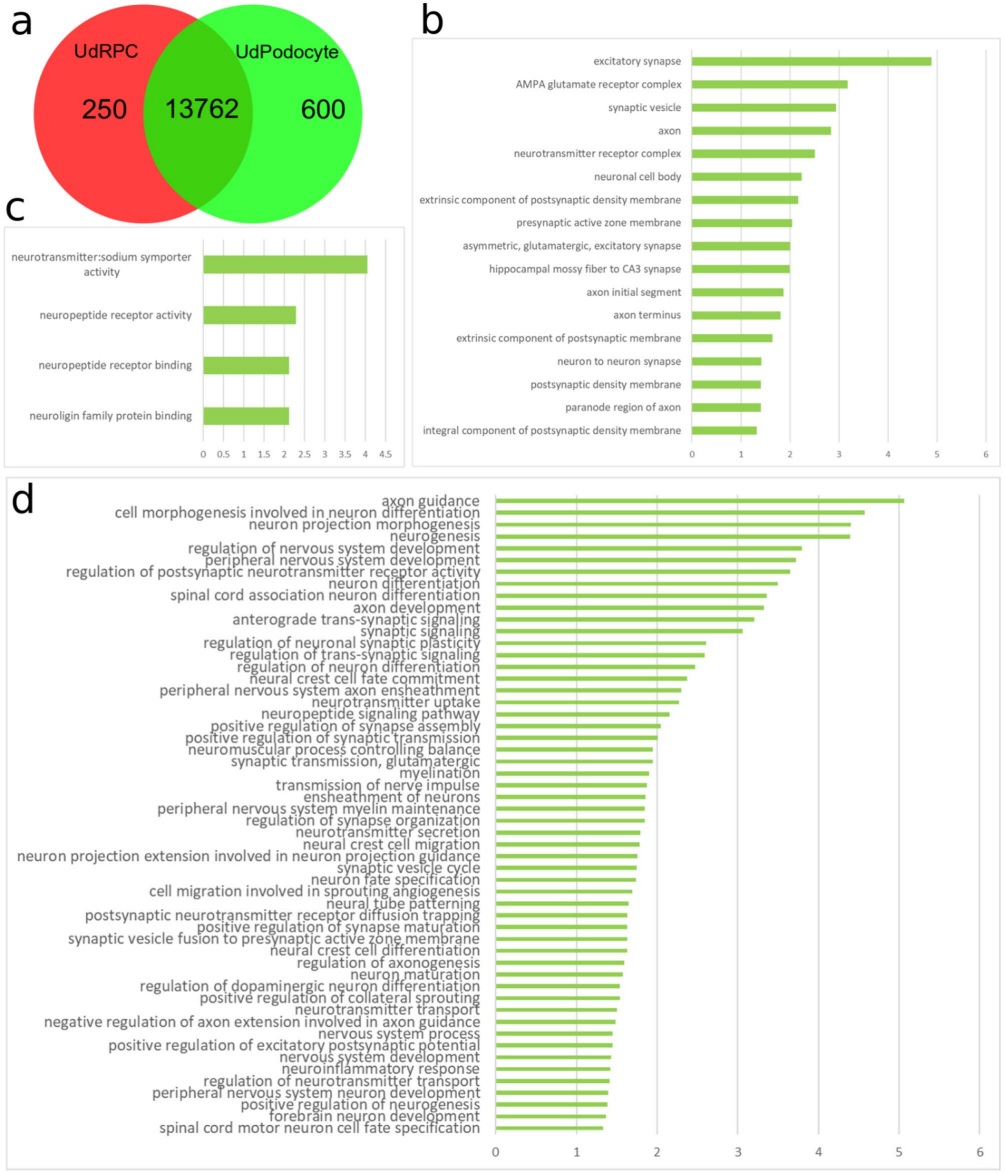
Brain-associated biological processes are enriched in Urine-derived Podocytes (UdPodocytes). Genes expressed exclusively in UdPodocytes in a Venn diagram comparison (**a**) with urine-derived SIX2-positive renal progenitor cells (UdRPCs) were subjected to gene ontology (GO) over-representation analysis and brain-related terms were extracted from them. From the resulting list of GOs (**b**) the cellular components, (**c**) the molecular functions and (**d**) the biological processes with a p-value less than 0.05 are plotted here with bars indicating their negative logarithmic (base 10) p-value

This study was initiated by the observation of a plethora of over-represented brain related GOs in transcriptomes of UdRPCs differentiated into podocytes [14, 34]. A probable explanation for this result is that there is obviously more transcriptome data available of the brain than in the kidney and as such much more GO annotations exist for brain. Nevertheless, these results also demonstrate the similarity in cell-cell communication-associated molecular mechanisms between podocytes and brain. Figure 2 shows neuron-related GO terms extracted from a list of GOs significantly over-represented in urine-derived podocytes (UdPodocytes, 600 genes) extracted from a Venn diagram comparison of expressed genes between urine-derived renal progenitor cells (UdRPCs) and UdPodocytes (Fig. 2a, Figure S2). The most significant Cellular Component (CC) is *excitatory synapse* (Fig. 2b) the most significant Molecular Function (MF) is *neurotransmitter: sodium symporter activity*. The three most significant Biological Processes in the list of GO terms are *axon guidance*,* cell morphogenesis involved in neuron differentiation* and *neuron projection morphogenesis* (Fig. 2d). A more detailed analyses of the genes associated with *axon guidance* revealed genes encoding Ephrins, Slits, and Semaphorins (Figure S3). These ligands and receptors are responsible for the regulation of the actin cytoskeleton on the one hand and on the other hand for attraction and repulsion. In summary, the GO terms can be categorized into groups related to neurons including amongst others *neurogenesis* and *neuron differentiation*, neuron projections including *neuron projection morphogenesis*, axons including *axon guidance* and *axon development* and synapses including *positive regulation of synapse assembly*.

### Comparison of brain-associated gene expression in urine-derived podocytes and in iPSC-differentiated brain and kidney organoids

**Fig. 3:**
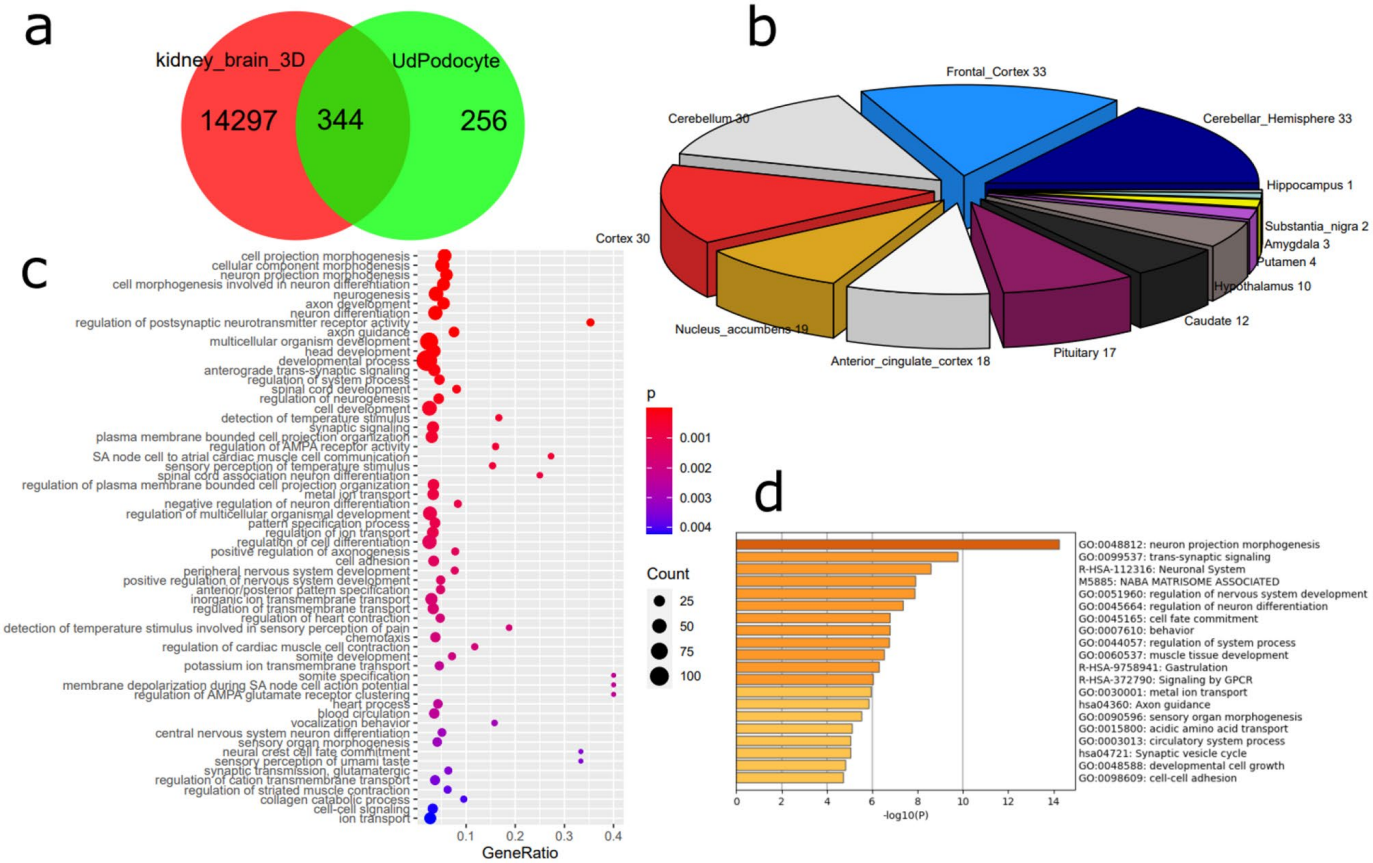
The overlap between urine-derived Podocytes (UdPodocytes) with brain and kidney organoids shows over-representation of the frontal cortex brain region and biological categories such as cellular morphogenesis, neuron differentiation, synaptic signaling and axon guidance. (**a**) Genes expressed in kidney and brain organoids were compared with the 600 genes identified as exclusively expressed in UdPodocytes resulted in an intersection of 344 genes. (**b**) The 344 genes map to multiple brain regions with most hits in frontal cortex. (**c**) Results of the GO analysis using the 344 genes expressed in common revealed biological terms from categories such as *cellular morphogenesis*,* neuron differentiation*,* synaptic signaling* and *axon guidance*. (**d**) Mapping genes to the GO: Molecular Function (GO-MF) top category shows that most of the 344 genes are associated with binding and transcription factor activity and submitting the 294 genes associated with the top 20 GO-MFs to Metascape analysis besides similar biological processes as in (**c**) reveals involvement of the extra-cellular matrisome

We further compared the genes associated with neuron-related biological processes in urine-derived podocytes detected above with transcriptome data from brain and kidney organoids. Figure 3a shows a venn diagram comparing the genes expressed in kidney and brain organoids with the 600 genes identified to be exclusively expressed in UdPodocytes. The resulting intersection set of 344 genes were mapped to brain regions via associations retrieved from the GTEX database (Fig. 3b) showing distribution over multiple brain regions with most hits and most significant over-representation in the frontal cortex (*p* = 0.0006, hypergeometric test). GO analysis via the R package GOStats [27] was applied to detect over-represented GO terms in the 344 genes (Fig. 3c). This revealed categories such as *cellular morphogenesis*,* neuron differentiation*,* synaptic signaling* and axo*n guidance*. In a follow-up REVIGO analysis the “Biological Process”-associated GO terms “*cell projection morphogenesis*” and “*regulation of postsynaptic neurotransmitter receptor activity*” were central hubs (Figure S4). Moreover, Fig. 3d and Table S2g depict results of a Metascape analysis based on the 20 GO terms of the category “Molecular Function” with most matches of the 344 genes. (approximately 70% of the 344 genes are associated with binding activity while 25% are associated with catalytic, 13% with hydrolase and 10% with transporter activity) yielding similar results as Fig. 3c. Over-representation analysis of the 344 genes with the EnrichR tool [28] revealed SUZ12, NFKB1 and PAX2 as most significantly over-represented transcription factors (Table S2c-e) and also transcription factors including PAX6, PAX7, SOX2 and GATA4 associated with TF-associated GO Molecular Functions *DNA-binding*,* transcription regulator activity*, etc. (Table S2f) which were partially validated via kidney expression in the Protein Atlas [36] (Table S2h). These validated proteins could be connected in a protein interaction network of Biogrid interactions using EGFR and XPO1 as major hubs (Figure S5).

### The protein interaction network of UdPodocytes genes associated with brain

**Fig. 4:**
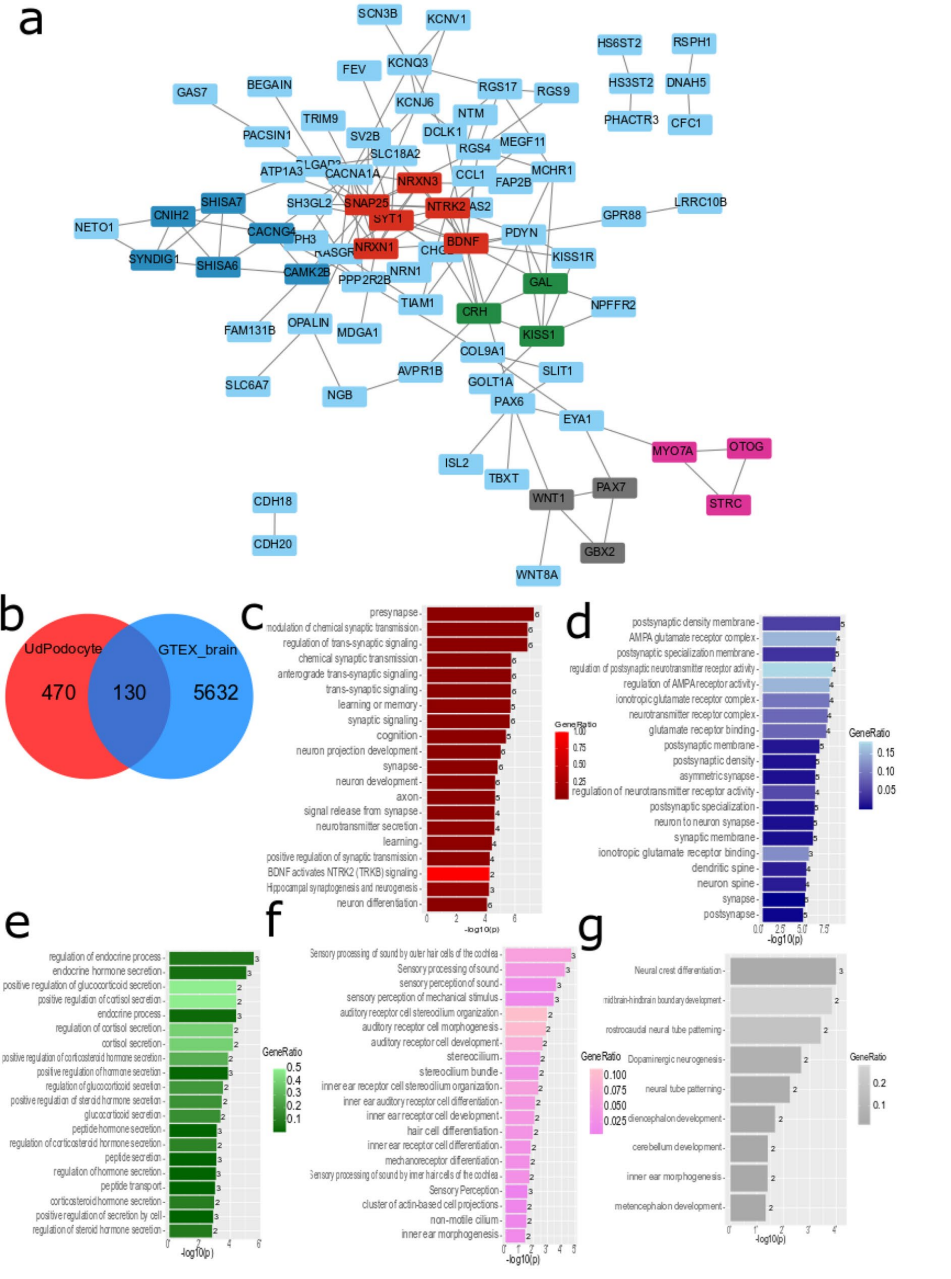
The protein interaction network of UdPodocytes genes associated with brain in GTEX contains modules for pre-synapse, post-synapse, endocrine processes, cochlea and neural crest differentiation. (**a**) STRING protein interaction network of 130 genes resulting from the venn diagram comparison of genes exclusively expressed in UdPodocytes and genes associated with brain in the GTEX database (**b**). Analysis of the STRING network in Cytoscape and MCODE yielded several subnetworks of which the most significant five MCODE1-5 are highlighted. Bar charts in panels (**c-g**) show biological terms found enriched in the subnetworks MCODE1-5 with the corresponding color. (**c**) In subnetwork MCODE1 *presynapse* is the most significantly enriched biological term. (**d**) In subnetwork MCODE2 *postsynaptic density membrane* is the most significantly enriched biological term. (**e**) In subnetwork MCODE3 *regulation of endocrine process* is the most significantly enriched biological term. (**d**) In subnetwork MCODE4 *Sensory processing of sound by outer hair cells of the cochlea* is the most significantly enriched biological term. (**e**) In subnetwork MCODE5 *Neural crest differentiation* is the most significantly enriched biological term

Figure 4a shows the protein interaction network of the 130 genes from the intersection set of the venn diagram comparison of genes exclusively expressed in UdPodocytes and genes associated with brain in the GTEX database (Fig. 4b). In a REVIGO analysis of the “Biological Process”-associated GO terms found over-represented in the 130 gene-set, “*neurogenesis*” and “*regulation of trans-synaptic signaling*” were central hubs (Figure S6). Furthermore, we submitted the 130 genes to the STRING online database, added no further interacting proteins but removed unconnected proteins. The STRING network was then loaded into Cytoscape and analysed with the MCODE plugin to identify clusters/subnetworks. In Fig. 4a the most significant five subnetworks MCODE1-5 are highlighted. Bar charts in Fig. 4c-g show biological terms found enriched in the subnetworks MCODE1-5 with the corresponding color. Subnetwork MCODE1 (Fig. 4c, red) contains the proteins NTRK2, SNAP25, SYT1, BDNF, NRXN1 and NRXN3 and is most significantly enriched with the GO *presynapse*. Subnetwork MCODE2 (Fig. 4d, blue) contains the proteins SYNDIG1, CNIH2, CACNG4, SHISA7, SHISA6 and CAMK2B and is most significantly enriched with the GO *postsynaptic density membrane*. Subnetwork MCODE3 (Fig. 4e, green) contains the proteins CRH, GAL and KISS1 and is most significantly enriched with the GO *regulation of endocrine process*. Subnetwork MCODE4 (Fig. 4f, violet) contains the proteins MYO7A, OTOG and STRC and is most significantly enriched with the GO *Sensory processing of sound by outer hair cells of the cochlea*. Subnetwork MCODE5 (Fig. 4g, grey) contains the proteins GBX2, PAX7 and WNT1 and is most significantly enriched with the GO *Neural crest differentiation*. mRNA expression of *KCNQ3*,* PAX6* and *TUBB3* which are amongst the 130 selected genes were confirmed in four distinct podocyte cultures and the immortalized podocyte cell line (Figure S7, primers used Table S6) and protein expression was confirmed in the Human Protein Atlas (proteinatlas.org) [36] (Figure S8). The expression of RPL0 was measured as a housekeeping gene.

### Expression of common brain associated proteins

In our previous manuscripts, we showed the capacity of urine-derived renal progenitors (UdRPCs) to differentiate into podocytes [14, 34, 17, 37] as well as tubular epithelial cells [38]. Supplemental Figure S9 + S10 depicts an immunofluorescence staining confirming the differentiation capacity of five UdRPC preparations used in this study. Podocyte differentiation was validated using two established markers, the cytoskeletal protein α-actinin-4 (ACTN4) and the slit diaphragm protein Nephrin (NPHS1) (Figure S9 A + B).

To demonstrate their bi-potential capacity, UdRPCs were differentiated in tubular epithelial cells. Immunofluorescence-based detection revealed that podocytes did not express tubular markers such as AQP1, CLCNKB and Na^+^-K^+^-ATPase as seen in the tubular differentiated cells. Secondary antibody alone was negative, confirming specificity (Figure S10).

**Fig. 5:**
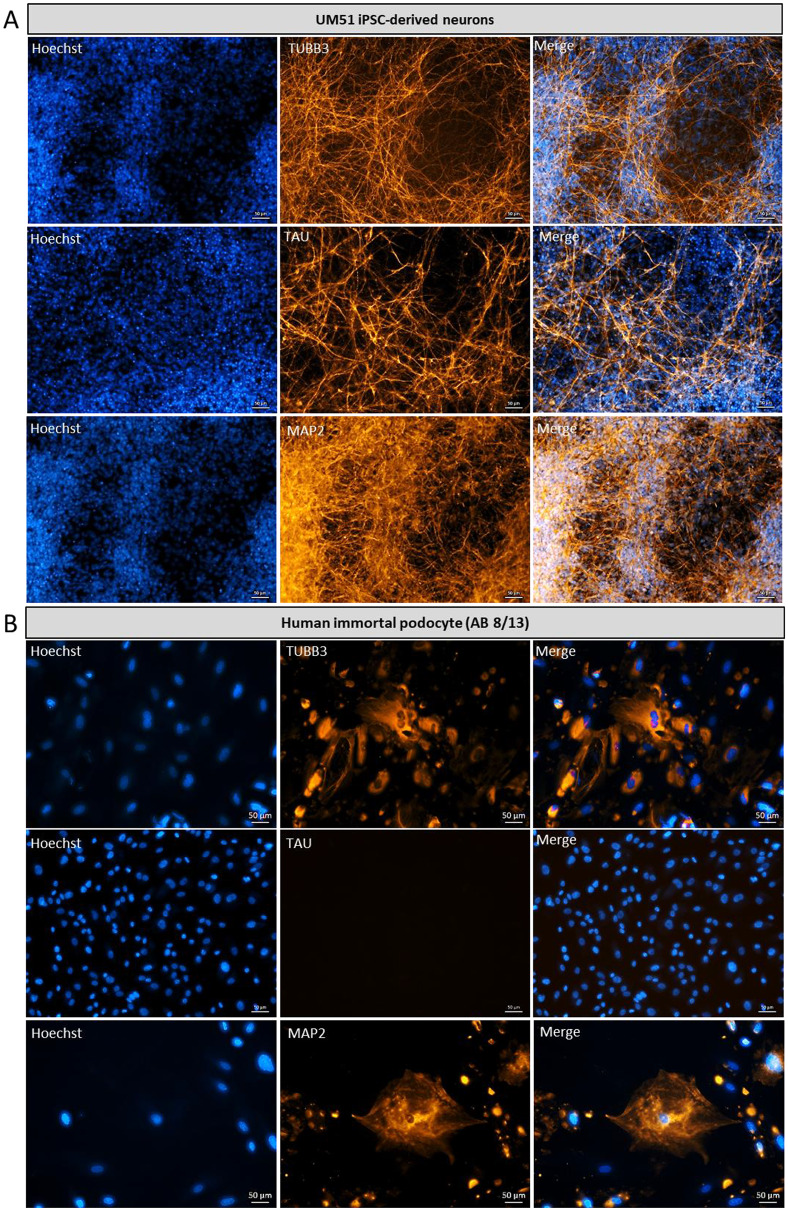

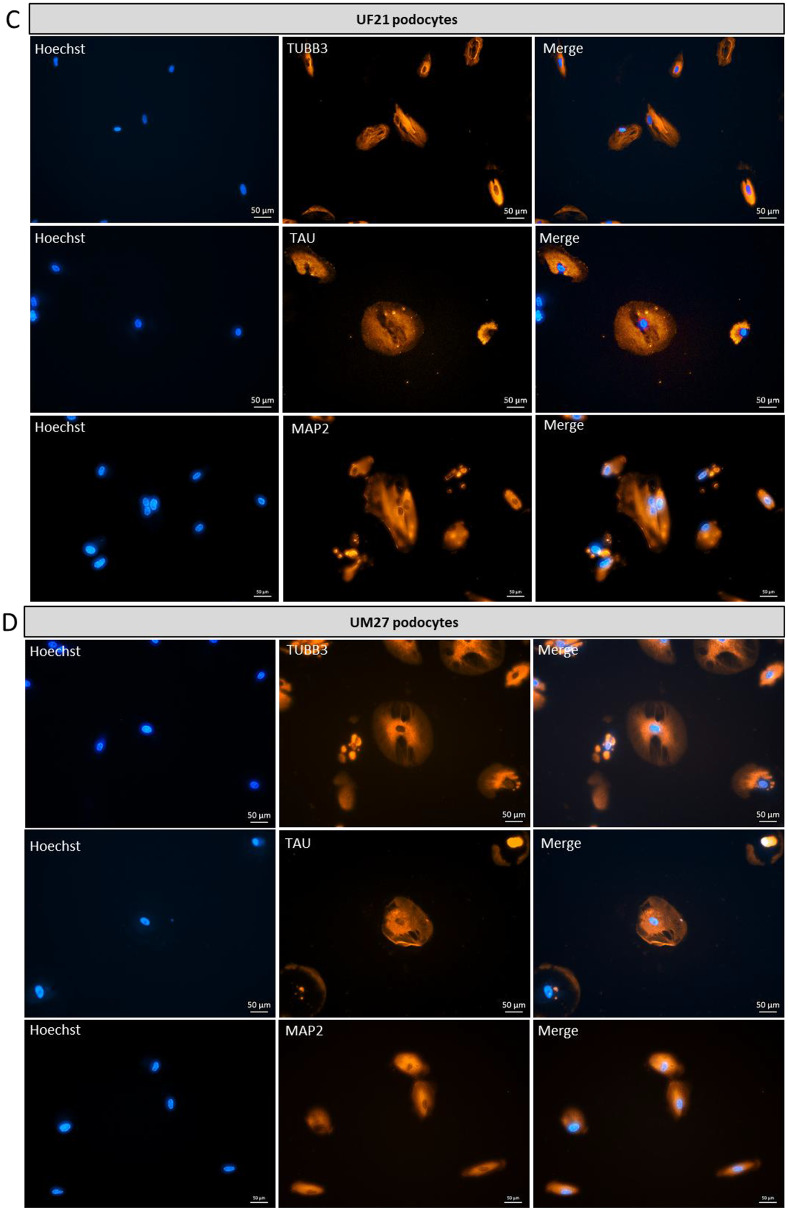

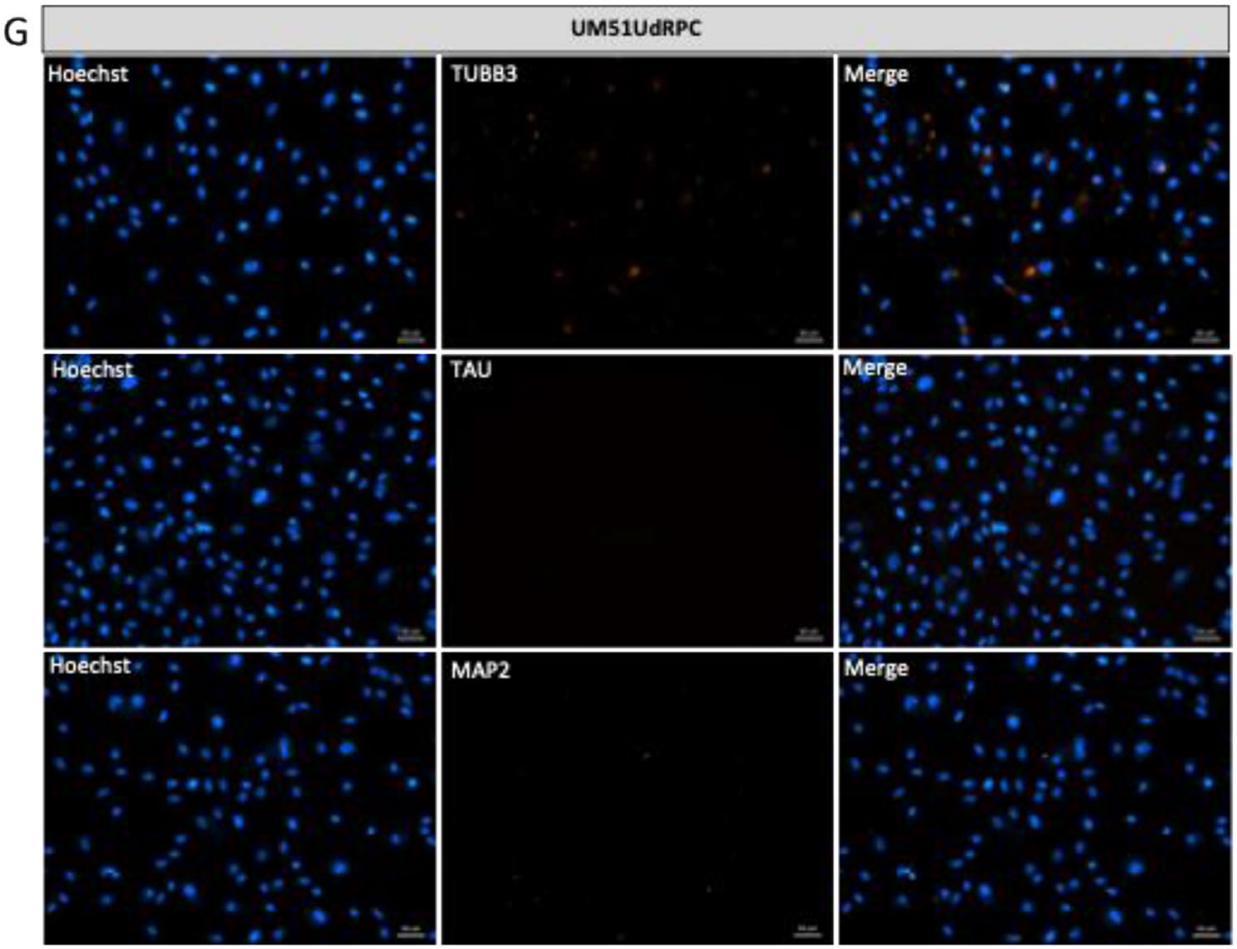
Expression of common brain-associated markers TUBB3, MAP2 and TAU in human podocytes. Urine-derived nephron progenitor cells (UdRPC) and the immortalized podocyte cell line (AB 8/13) were differentiated into podocytes by culturing at 60–70% density in Advanced RPMI supplemented with 30 µM retinoic acid (RA). In parallel urine-derived iPSC were differentiated into neurons. (**A**) Representative immunofluorescence staining images of TUBB3, MAP2 and TAU expressed in iPSC-derived neuronal cultures. Scale bar 50 μm. (**B-F**) As common brain-associated proteins, the urine-derived podocytes and the immortalized podocyte cell line (AB 8/13) express TUBB3, MAP2 and TAU. Scale bar 50 μm. (**G**) Representative negative control UM51 UdRPCs immunofluorescence staining showed, that they did not express the neuronal marker TUBB3, MAP2 and TAU in comparison to the differentiated podocytes. Scale bar 50 μm

**Fig. 6:**
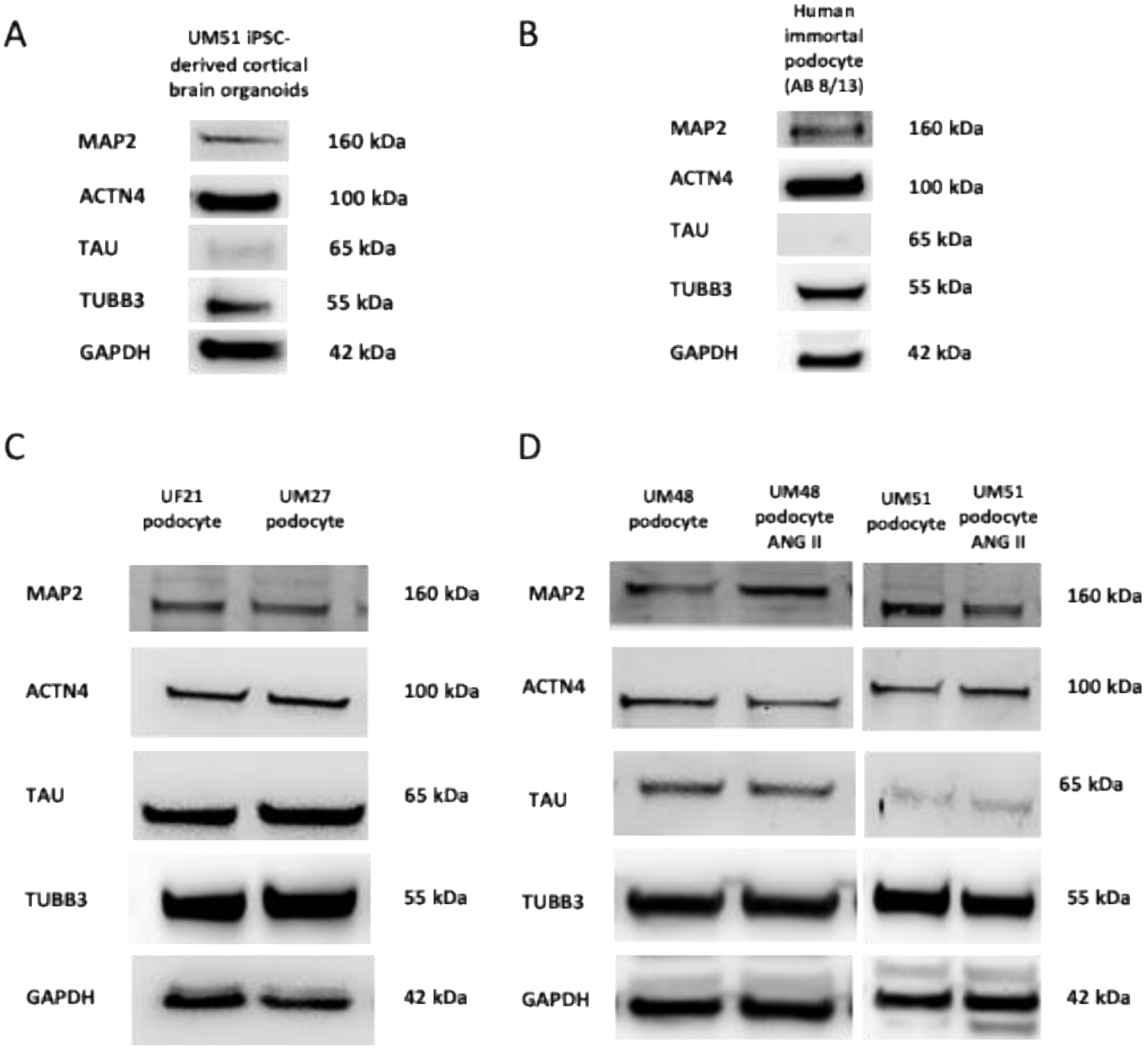
Western Blot analyses of brain-associated proteins expressed in human podocytes and iPSC-derived cortical brain organoids. (**A**) Representative western blot analysis of the neuronal markers MAP2, TUBB3 and TAU and the podocyte marker-ACTN4 confirmed in iPSC-derived cortical brain organoids. (**B**) The human immortal podocyte (AB 8/13) reference cell line expressed all detected proteins besides the neuronal marker TAU, which was not detectable. (**C + D**) Urine-derived podocytes expressed brain-associated proteins such as MAP2, TAU, TUBB3 and the podocyte specific marker ACTN4

To investigate the expression of common brain-associated proteins, urine-derived podocytes, a reference the human immortal podocytes (AB 8/13) and iPSC-derived neurons were subjected to immunofluorescence staining for the neuronal marker βIII-tubulin (TUBB3), microtubule-associated TAU (MAPT) and Microtubule Associated Protein 2 (MAP2) (Fig. 5A-F). Undifferentiated UdRPCs served as a negative control (Fig. 5G). Staining for specific neuronal markers revealed distinct differences in labelling patterns. Neuronal cultures displayed a well-defined, interconnected neuronal network, whereas podocytes exhibited predominantly cytoplasmic staining without network formation. These results were corroborated by Western blot analysis, confirming the expression of neuronal proteins (Fig. 6A–D).

For normalization, expression glyceraldehyde-3-phosphate dehydrogenase (GAPDH) was used for all western blots. Full uncropped Western blot images are presented in Supplementary Figure S11.

### Responsiveness of genes expressed in podocytes and brain to the renin-angiotensin-system mediator- ANG II

**Fig. 7:**
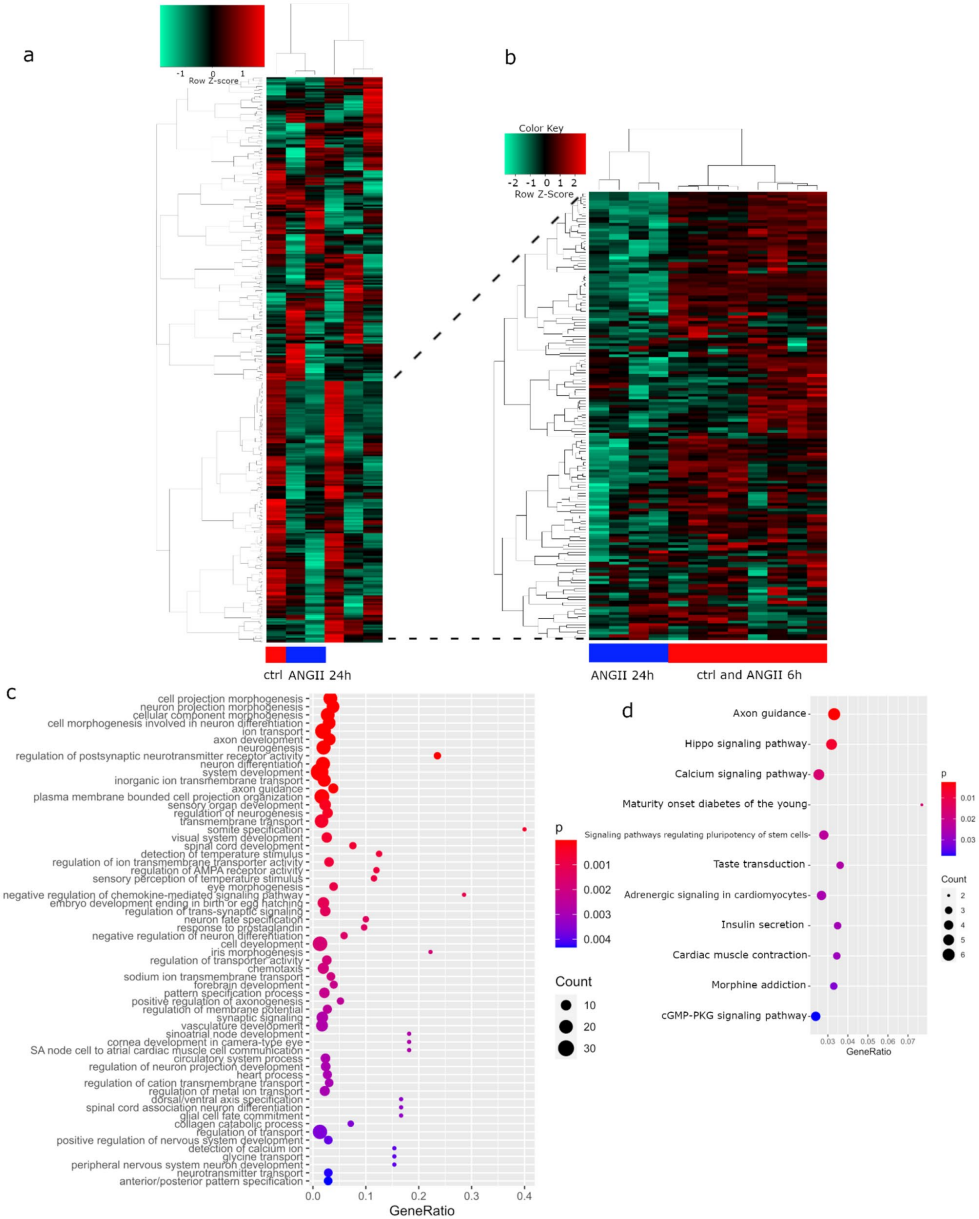
The 344 genes overlapping between UdPodocytes and kidney and brain organoids are responsive to ANGII stimulation. (**a**) cluster analysis and heatmap of the 344 genes overlapping between UdPodocytes and kidney and brain organoids in UdPodocytes untreated (ctrl, red color bar), treated 24 h with ANGII (blue color bar) and other treatments including the AT1R antagonist Losartan (no color bar). This cluster analysis dissected one cluster which was down-regulated upon 24 h of ANGII treatment and on which we focused in the follow-up analysis. (**b**) cluster analysis and heatmap in UdPodocytes from two individuals using the genes from the ANGII-down-regulated cluster in UdPodocytes untreated (ctrl, red color bar), treated 6 h with ANGII (effect not yet apparent, also red color bar) and treated 24 h with ANGII (blue color bar). (**c**) The 60 most significantly over-represented gene ontologies of type Biological Process include terms related to *projection morphogenesis*,* axon development* and *AMPA receptors*. (**d**) KEGG pathways significantly over-represented (*p* < 0.05) include *axon guidance*,* Hippo signaling*,* Calcium signaling and cGMP-PKG signaling*

Figure 7a shows a cluster analysis and heatmap of the 344 genes overlapping between UdPodocytes and kidney and brain organoids in UdPodocytes untreated (ctrl, red color bar), treated 24 h with ANGII (blue color bar) and other treatments including the Angiotensin-II-type I receptor (AT1R) antagonist Losartan (no color bar). Following our previous publications showing down-regulation of key molecules of the actin cytoskeleton by ANG II [14] and beneficial effects of the ATR1 blocker - Losartan [34] we were interested in down-regulated gene clusters upon 24 h of ANGII treatment. We then used these genes for a follow-up cluster analysis and heatmap (Fig. 7b) in UdPodocytes from two individuals untreated (ctrl, red color bar), treated 6 h with ANGII (effect not yet apparent, also red color bar) and treated 24 h with ANGII (blue color bar). Furthermore, genes from the cluster down-regulated by ANG II were subjected to GO analysis (Fig. 7c, Suppl. Table S4) and revealed terms related to *projection morphogenesis*,* axon development* and *AMPA receptors* among the most significantly over-represented Biological Processes. Over-representation analysis results of KEGG pathways (Fig. 7d) included significant (*p* < 0.05) *axon guidance*,* Hippo signaling*,* Calcium signaling* and *cGMP-PKG signaling.*

### Comparison of synaptic pathways between brain and podocytes

**Fig. 8:**
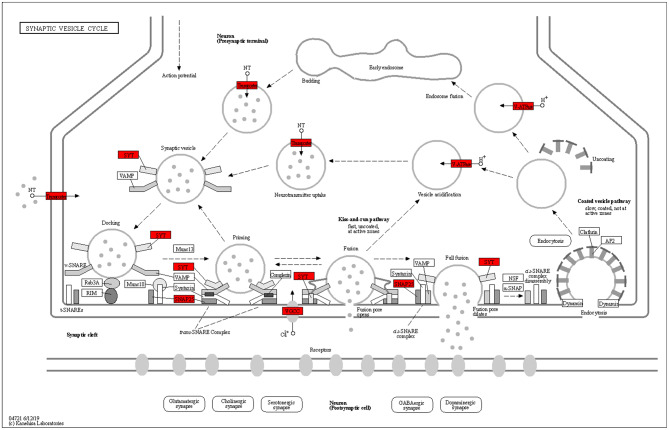
Most genes of the synaptic vesicle cycle are expressed in podocytes and in brain. The 344 genes expressed in UdPodocytes and in kidney as well as in brain organoids were subjected to EnrichR analysis and the KEGG pathway hsa04721 *Synaptic vesicle cycle* was found as one of the most enriched pathways (*p* = 3.89 10^− 4^, adjusted_p = 0.037). Genes in crucial locations of the pathway are involved as illustrated by the boxes with red shading

Figure 8 is an illustration of the 344 genes expressed in common between UdPodocytes, kidney and brain organoids which were found enriched (*p* = 3.89 10^− 4^, adjusted_p = 0.037) in the KEGG pathway hsa04721 *Synaptic vesicle cycle* via EnrichR enrichment analysis. The involved genes were mapped to the pathway chart and marked with red shading. The pathway chart in Fig. 8 shows that most genes of the synaptic vesicle cycle are also expressed in podocytes therefore implying overlapping mechanisms in neurotransmitter uptake, Calcium signalling and vesicle processing.

**Fig. 9:**
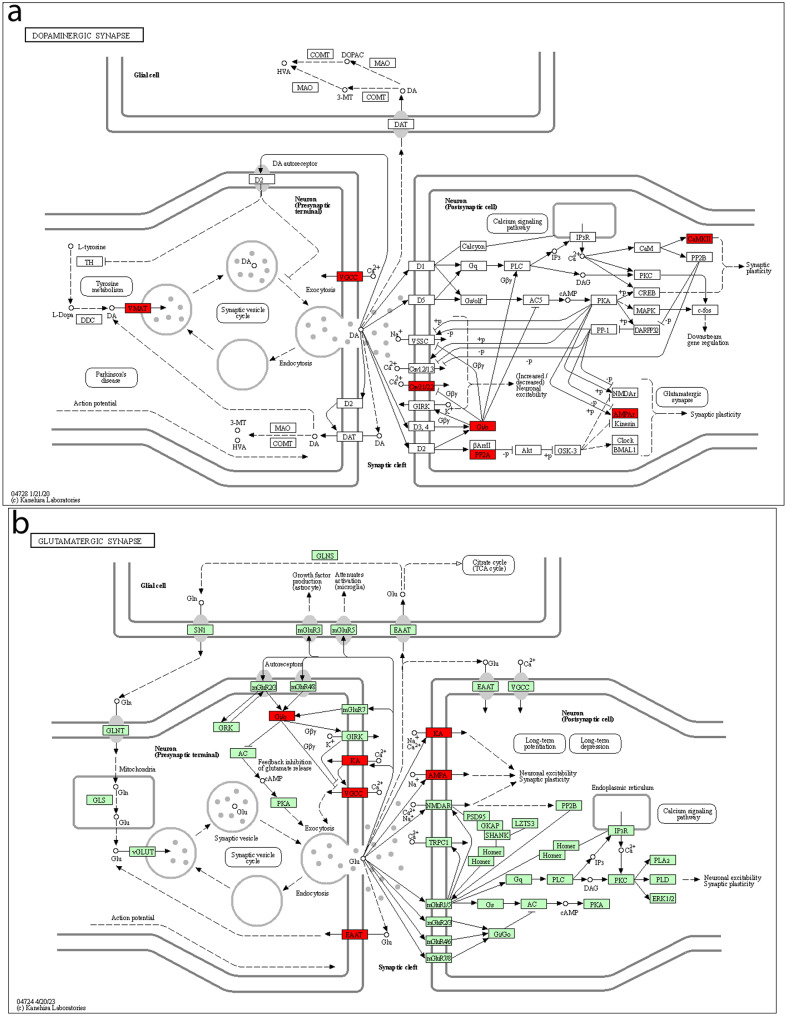
Genes of the pre- and post-synaptic parts of the Dopaminergic and Glutamatergic synapse are expressed in podocytes and brain. The 344 genes expressed in UdPodocytes and in kidney as well as in brain organoids were subjected to EnrichR analysis and the KEGG pathways (**a**) hsa04728 *Dopaminergic synapse* (*p* = 0.027, adjusted_p = 0.485) and (**b**) hsa04724 *Glutamatergic synapse* (*p* = 0.047, adjusted_p = 0.485) were found amongst the most enriched pathways. Genes in crucial locations of the pathways, e.g. Dopamine uptake into vesicles, synaptic plasticity and calcium signaling, are involved in the Dopaminergic synapse (**a**) and glutamate receptors of AMPA and kainate types triggering neuronal excitability and synaptic plasticity in the Glutamatergic synapse (**b**), as illustrated by the boxes with red shading

In Fig. 9a, genes from the KEGG pathway hsa04728 *Dopaminergic synapse* expressed in common in UdPodocytes, kidney and brain organoids are shown. The EnrichR enrichment analysis identified this pathway as the most significant specific synapse-related pathway (*p* = 0.027, adjusted_p = 0.485). The pathway map shows additionally numerous genes of post-synaptic cells are expressed in podocytes and kidney organoids. CACNA1C (indicated in the map as Cav 2.1/2.2) involved in the calcium channels has been reported to be a major player in polycystic kidney disease [39]. In general, ion channels mediate the homeostasis of electrolytes and mutations in the genes coding them may lead to renal disease [40, 12]. Furthermore, the KEGG pathway- hsa04724 *Glutamatergic synapse* (*p* = 0.047, adjusted_p = 0.485) was found over-represented (Fig. 9b) and thus may imply similar mechanisms in podocytes and diverse types of synapses.

## Discussion

In this study we compared gene expression in podocytes and brain and unveiled considerable overlap. Starting with genes expressed exclusively in UdPodocytes [14, 34] in comparison to SIX2-posive UdRPCs [17] we identified a plethora of genes over-represented in brain-associated GOs such as *synapses*,* synaptic signaling*,* neuron projections/axons* and *myelination*. Our in vitro model revealed expression of Nephrin (NPHS1) and intercellular projections upon differentiation of UdRPCs into UdPodocytes (Fig. 1). In this study, we observed intercellular projections developing sporadically, however, the conditions under which they occur and their exact frequency will be an important issue to be investigated in follow-up studies. The major function of NPHS1 as a cell adhesion molecule that functions in the glomerular filtration barrier in the kidney has been widely described [41]. Moreover, expression of NPHS1 has been identified in brain [35] where Putaala et al. conclude that it has a developmental role and is mainly expressed in radial glia cells [35]. Li et al. report Nephrin expression close to synaptic proteins in primary neuronal cells and co-immunoprecipitation with synaptic molecules including glutamate receptors and PSD95 and hypothesize that podocytes and brain cells use similar organization and signaling mechanisms [42]. One crucial player in the organization of foot processes and synapses is ACTN4, which bundles F-actin in the cytoskeleton and cross-links it to developing processes, in neurons and podocytes acting together in a network with glutamate receptors [43, 42]. These control the plasticity of dendritic spines in neurons in response to signaling events [43]. A corresponding mechanism of podocyte glutamatergic signaling participating in the integrity of the glomerular filtration barrier was reported by Giardino et al. who identified foot process effacement in a knock-out mouse model of Rab3a which regulates exocytosis of glutamate [5]. In our human UdPodocyte model, we found ACTN4 expression connecting the cytoskeleton of podocytes.

Another Actin-associated protein SYNPO, which is evident in the present transcriptome data, regulates the cytoskeleton in mature podocytes, where it is essential for the functioning of podocyte foot processes. Previous studies have shown that SYNPO also plays a role in the formation of spine apparatuses in spines of telencephalic neurons, which is involved in synaptic plasticity [8, 9]. Dendritic spines are small thorn-like protrusions found on the dendrites of most of the excitatory neurons in the brain. They are the point of contact between neurons, forming the post-synaptic side of the excitatory synapses, and orchestrate the molecular machinery that allows the transmission of signals from the afferent neurons [44–47]. The stability of the spines is associated with SYNPO [48–51]. Studies have shown that deletion of SYNPO in mice results in deficits in spatial learning [52, 53]. Furthermore, podocytes lacking SYNPO acquire cytoskeletal alterations, which results in damage to the podocytes and disturbance of the foot processes and the slit diaphragm [54, 8].

As previously described, podocyte-associated markers such as SYNPO and NPHS1 are also expressed in neuronal cells, underscoring the well-recognized parallels between podocytes and neurons. In the present study, we focused on classical brain-associated markers that are essential for axon formation and neuronal network organization, such as MAP2, TAU and TUBB3, and investigated their expression in podocytes.

Podocytes are characterized by highly specialized cellular extensions, the so-called foot processes, which share structural and functional similarities with neuronal projections, particularly with regard to signal transmission and cytoskeletal dynamics. Our results demonstrate that podocytes indeed express the neuronal markers MAP2, TAU and TUBB3. Notably, however, their subcellular localization differs from that observed in neurons. While neuronal cells predominantly exhibit cytoskeletal-associated staining patterns for these proteins, podocytes display a primarily cytoplasmic localization.

Previous studies have shown that all three markers share a common functional property in neurons: when expressed in the cytoplasm, they bind to and stabilize microtubules [55–57]. Podocytes likewise possess a complex microtubule network that is essential for maintaining cytoskeletal integrity, preserving cellular architecture, regulating foot process formation, and facilitating intracellular transport [58–60]. In this context, our findings suggest that podocytes may utilize these brain-associated proteins in a similar manner to support microtubule stability and cytoskeletal organization. Importantly, inhibition or loss of these proteins appears to impair cytoskeletal integrity, which may contribute to cellular damage. Given that microtubule dysfunction is implicated in both neurodegenerative disorders and podocyte injury, the shared expression and potential functional overlap of MAP2, TAU, and TUBB3 may represent a common mechanistic pathway. Such alterations could ultimately contribute to podocyte damage, progression of kidney disease, and severe long-term renal consequences.

In our study we showed that expression of ACTN4, TUBB3 and TAU is down-regulated upon ANGII stimulation in podocytes [13].

With respect to these partially similar signaling mechanisms in podocytes processes and neuronal synapses, Weide and Huber ask what is pre- and post-synaptic [61] referring to the publication of Giardino et al. who report Myosin IIA at post-synapses and in foot processes [5]. Moreover, Mundel and colleagues report Synaptopodin (SYNPO) at podocyte foot processes and dendritic spines [9] and Kobayashi and colleagues [6] find the typical postsynaptic density protein Densin localised in the cytoplasm beneath the slit membrane.

UdPodocytes constitute a promising model for studying cytoskeletal dynamics in healthy and diseased podocyte.

Genes such as *SERPINI1*, *RSPH1*, *KCNQ3* and *PAX*6 are also expressed in both brain and podocytes (Table S2). PAX6 is a crucial transcription factor associated with brain development and function. It has been shown to regulate genes associated with neural stem and progenitor cells. Studies have shown molecular interactions, such as the regulation of cell-cell adhesion or ion transport [62, 63].

PAX2 and PAX8 are crucial regulators of multiple steps of renal system development [64]. However, to date studies associating PAX6 function in nephrogenesis is lacking. SERPINI1 is a Serine protease inhibitor that inhibits plasminogen activators and plasmin but not thrombin [65–69]. It is involved in the formation or re-organization of synaptic connections as well as for synaptic plasticity in the adult nervous system and protects neurons from cell damage by tissue-type plasminogen activator and probably plays the same role in kidney.

The gene *KCNQ3*, encodes a potassium channel protein that functions in the regulation of neuronal excitability and is considered to be a key characteristic of activity-dependent synaptic plasticity of neurons [70]. One of the fundamental functions of renal K channels is their role in generating the cell-negative potential that provides one of the main driving forces for the passive movement of charged solutes and carrier molecules across the apical and basolateral membranes of tubule cells [71].

In-depth analyses of the 600 brain-annotated genes expressed in UdPodocytes revealed GOs such as *excitatory synapse* and *AMPA glutamate receptor complex*. Amongst this list, GRIA3 has been reported to be epigenetically down-regulated in podocytes under diabetic conditions [72]. Additionally, we found AMPA receptor over-represented among genes expressed in brain and podocytes and down-regulated upon ANGII stimulation of Upodocytes. AMPA belongs to the group of ionotropic glutamate receptors [73] and the same holds for NMDA receptors which are more intensively studied with regards to their role in regulating ion channels in podocytes [74, 75]. In comparison to neurons, NMDA receptors have overlapping and distinct features in podocytes, e.g. unlike in neurons they are resistant to activation by L-glutamate and L-aspartate and show minimal inactivation upon continuous NMDA supply, but similar to neurons they can be activated by D-aspartate and L-homocysteic acid [74]. Staruschenko et al. propose that NMDA receptors in podocytes are more adapted to slowly flowing metabolites in contrast to fast synaptic signaling processes. Our finding of AMPA receptor expression in podocytes together with Li et al. ’s report of down-regulated AMPA-associated GRIA3 in podocytes under diabetic conditions [72] suggests that further studies investigating the role of AMPA receptors in podocyte physiology will be promising. A body of publications describe ANGII signaling in the brain and beyond that a brain renin-angiotensin system which despite profound evidence [76–79] is also a subject of debate [79]. Persistent activation of ANGII signaling can lead to neurodegeneration and Angiotensin receptor blockers (ARBs) possess therapeutic potential [79]. Besides its role in regulating blood pressure, ANGII has also been reported to act as a neurotransmitter [80] which can interact with glutamate, possibly acting on glutamate receptors and also mediating increase of blood pressure, upon glutamate administration into the paraventricular nucleus (PVN).

We identified Calcium signaling as associated with the ANGII down-regulated geneset. Greka and Mundel demonstrated that ANGII triggers - Calcium (Ca2+) flow into podocytes via transient receptor potential canonical 6 (TRPC6) and TRPC5 channels and modulate actin cytoskeleton [81], by interacting with Ca2+-activated phosphatase, Calcineurin, and Rho GTPases which are crucial determinants of podocyte function. Impairments in these channels e.g. by mutations in TRPC6 can lead to Focal Segmental Glomerusclerosis (FSGS). Besides Calcium signaling, Hippo Signaling, cGMP-PKG signaling and axon guidance pathways were also over-represented. cGMP-bound ion channels regulate sodium uptake and excretion [82]. Chen et al. [83] described YAP and TAZ, as key players in Hippo signaling which are important for the integrity of podocytes and TAZ together with the transcription factor TEAD activate crucial podocyte-associated genes such as *SYNPO*, *ZO-1* and *ZO-2* (synaptopodin, zonula occludens-1, and zonula occludens-2). Interestingly, proteins from the axon guidance pathway were found to be associated with risk of end-stage kidney disease in diabetes [84]. The genes in our ANGII-down-regulated signature are all upstream of the actin-cytoskeleton-regulation complex within the axon guidance pathway thus pointing at down-regulation of actin cytoskeleton by ANGII treatment, in line with our previous publication showing ANGII-dependent downregulation of *NPHS1* and *SYNPO* and subsequent disruption of the podocyte cytoskeletal architecture [14]. Moreover, Shao et al. [85] revealed that HNF1B regulates axon guidance-associated genes in the developing mouse kidney and suggest that axon guidance ligand receptor pairs of neighboring cells, some also from the ureteric bud, may orchestrate kidney development via auto- and paracrine signaling. This may be underlined by Combes et al. 2019 [86] who found axon-guidance associated GDNF-RET ligand receptor crosstalk in single-cell analysis of the developing mouse kidney. Analysing the 600 UdPodocyte-specific genes in the axon guidance pathway revealed numerous ligand receptor pairs which regulate both actin cytoskeleton and are responsible for attraction and repulsion of projections. Tapia et al. [87] described the axon-guidance-associated protein-Semaphorin 3A (SEMA3A) as a repulsive gene leading to foot process effacement and proteinuria upon exogenous induction of SEMA3A. Additionally, Lu and Zhu [88] describe involvement of several Semaphorins in diabetic nephropathy and partially aberrant foot processes in Sema3G-deficient mice. Hashimoto et al. [89] described co-localization of the axon guidance receptor Ephrin-B1 with Nephrin at the slit diaphragm between neighboring foot processes during development and is down-regulated in nephropathy. Furthermore, Wu et al. [90] describe a major role of the axon guidance factors Slit2 and Robo2 in podocyte attachment to the glomerular basement membrane and the structural integrity of foot processes. Taken together, it is tempting to speculate that foot processes partly use similar signaling mechanisms as axon guidance for their interdigitation and impairment of these signaling processes and lead to pathologically altered glomerular permeability.

In the follow-up analysis we identified abundant overlap of 344 genes expressed in common in UdPodocytes and in iPSC-derived brain and kidney organoids. The 344 genes expressed in common map to multiple brain regions with most hits in frontal cortex. As transcription factors enriched in the promoter regions of the 344 genes we found SUZ12 most significant (*p* = 8.42 × 10-22, EnrichR ENCODE and CHEA datasets), NFKB1 (*p* = 2.29 × 10-3, EnrichR TRANSFAC and JASPAR) and PAX2 (*p* = 1.59 × 10-3, EnrichR TTRUST). Mapping genes to the GO Molecular Function top category showed that about 70% of the 344 genes are associated with binding activity while about 25% are associated with catalytic activity.

Of note, we identified Brain-derived neurotrophic factor (BDNF) as central protein in one module associated with “pre-synapse” within our protein interaction network of brain-associated UdPodocyte genes (Fig. 4a, c). BDNF plays major brain-associated roles in synaptic plasticity, learning, memory, behavior, depression and neurodegenerative disorders such as Alzheimer’s disease [91], Interestingly, Endlich and colleagues proposed BDNF as marker for CKD in human urine cells. They found a strong correlation of BDNF expression with the podocyte marker Nephrin, the kidney injury marker KIM-1 and the urinary albumin‐to‐creatinine ratio in CKD patients [92]. Putatively connected to BDNF’s role in podocyte development, the authors showed de-differentiation of podocytes upon BDNF inhibition. Furthermore, Saito and colleagues reported another protein from this module, Neurexin-1 (NRXN1) known as presynaptic adhesion molecule associated with synaptic differentiation, expressed at the podocyte slit diaphragm, co-localized with Nephrin and CD2AP, and proposed it as marker of podocyte injury [93]. SNAP25, a further protein of this module, as well as BDNF were shown to be correlated with the Parkinson’s Disease (PD) -associated protein Parkin (PARK2), putatively via ubiquination of p21 and JNK, and inhibition of PARK2 led to down-regulation of BDNF and SNAP25 and impaired neural stem cell differentiation [94]. A study revealing an association between PD and impaired glomerular filtration [95] may be seen in this context.

We conclude that our study has revealed similarities between the transcriptomes of brain and podocytes which can be condensed into gene networks regulating (i) projections such as foot processes, axons and dendritic spines implicating modulation of actin cytoskeleton and (ii) signaling pathways such as Calcium-, Hippo- and cGMP- signaling happening at synaptic (e.g. glutamatergic and dopaminergic) or resembling constellations such as the slit diaphragm between interdigitating foot processes. Interestingly, axon guidance was found as most significant pathway in response to ANG II stimulation of podocytes which might point at similar mechanisms guiding axons and foot processes in kidney development and disease. This was in fact underlined by reports on ligand-receptor crosstalk of axon guidance molecules (GDNF-RET) for the developing kidney and association of axon guidance molecules with risk of end-stage kidney disease in diabetes [88, 96].

Interestingly, many of the brain-associated biological processes are currently not annotated for kidney. This points to the comparative lack of advancement in the annotation of kidney associated processes.

We therefore propose our urine-derived podocyte platform as a non-invasive and easily accessible in vitro model enabling the investigation of the functions of these brain-associated genes and biological processes in healthy and diseased kidneys.

## Electronic Supplementary Material

Below is the link to the electronic supplementary material.

Supplementary Material 1: Supplementary Table 1 (Table S1.xlsx): significantly over-represented GOs in the 600 genes subset only expressed in UdPodocytes from the Venn diagram of UdPodocytes vs. UdRPCs filtered for neuronal terms.

Supplementary Material 2: Supplementary Table 2 (Table S2.xlsx): (a) subsets from the Venn diagram comparing the 600 genes exclusively expressed in UdPodocytes with genes expressed in kidney and brain organoids. (b) Significantly over-represented GOs in the 344 genes subset expressed in common in UdPodocytes and brain and kidney organoids. (c) enriched transcription factors (TFs) found via the EnrichR analysis of the 344 genes using the ENCODE_and_ChEA dataset. (d) enriched TFs found via the EnrichR analysis of the 344 genes using the TRANSFAC_and_JASPAR dataset. (e) enriched TFs found via the EnrichR analysis of the 344 genes using the TRRUST dataset. (f) 20 most abundant GO Molecular Function (MF) terms amongst the 344 genes. (g) Metascape enrichment analysis results of genes in (f). (h) TFs from (f) validated for expression in kidney via the Proteinatlas.

Supplementary Material 3: Supplementary Table 3 (Table S3.xlsx): (a) subsets from the Venn diagram comparing the 600 genes exclusively expressed in UdPodocytes with genes expressed in brain in the GTEX database. (b) Significantly over-represented GOs in the 130 genes subset expressed in common in UdPodocytes and GTEX brain.

Supplementary Material 4: Supplementary Table 4 (Table S4.xlsx): (a) Significantly over-represented GOs in the genes down-regulated upon ANGII treatment amongst the 344 genes expressed in common in UdPodocytes and brain and kidney organoids. (b) GO terms in (a) filtered by neuronal and brain-related key words listed at the bottom of the sheet.

Supplementary Material 5: Supplementary Table 5 (Table S5.xlsx): Antibodies used in this study.

Supplementary Material 6: Supplementary Table 6 (Table S6.docx): Primer sequences used in this study.

Supplementary Material 7: Figure S1 (figure S1.pdf): Light microscope pictures of human podocytes within axon-like structures. Panels A-H show human urine-derived renal progenitor cells differentiated into podocytes highlighted with black arrows their axon-like structures in their podocyte development. Scale bars: 100 μm.

Supplementary Material 8: Figure S2 (figS2_flowchart.pdf): Flow chart of the comparison between brain and podocytes.

Supplementary Material 9: Figure S3 (fig S3_neuro_GOs600_hsa04360_axon_guidance.png): Genes related to the GO term “axon guidance”, the most significantly over-represented biological process in the 600 genes exclusively expressed in urine-derived Podocytes (UdPodocytes), marked within the KEGG pathway chart of axon guidance (hsa04360). Several guidance factors, such as ephrins, Slits, and semaphorins are expressed in the UdPodocytes. These ligands and receptors are responsible for the regulation of the actin cytoskeleton on the one hand and on the other hand for attraction of the projection while genes responsible for repulsion are missing. Genes exclusively expressed in the UdPodocytes are marked in red.

Supplementary Material 10: Figure S4 (figS4.pdf): Overlap between biological processes in urine-derived Podocytes (UdPodocytes) with brain and kidney organoids (344 genes) relates to cell projection morphogenesis and regulation of postsynaptic neurotransmitter receptor activity. (a) The gene ontology network was generated with the tools REVIGO and Cytoscape and summarizes GO-BP (Gene ontologies - Biological Process) terms found over-represented with a p-value < 0.01 in the 344 genes overlapping between UdPodocytes and brain and kidney organoids. Cell projection morphogenesis and regulation of postsynaptic neurotransmitter receptor activity -related terms emerged as representative for their clusters. GOs are represented by the network nodes with light red associated with the highest significance of over-representation of a GO term. The edges refer to similarities between the GO terms. (b) Treemap of the REVIGO tool corresponding to (a). The Treemap summarizes biological process overlapping between UdPodocytes and brain and kidney organoids. Representatives of the Treemap clusters include *cell adhesion*,* detection of temperature stimulus*,* metal ion transport*,* collagen catabolic process*,* developmental process*,* cell projection morphogenesis*,* actin-mediated cell contraction* and *regulation of postsynaptic neurotransmitter receptor activity*.

Supplementary Material 11: Figure S5 (figS5_net_TFs_reduced_opt.pdf): Protein interaction network of Proteinatlas-validated Transcription Factors (TFs) amongst the 344 genes subset expressed in common in UdPodocytes and brain and kidney organoids. The TFs validated for expression in the kidney via the Proteinatlas could be connected in a protein interaction network of Biogrid interactions using EGFR and XPO1 as major hubs. Green nodes are the original TFs and red nodes were added via Biogrid interactions.

Supplementary Material 12: Figure S6 (figS6.pdf): Overlap between biological processes in urine-derived Podocytes (UdPodocytes) with genes associated with brain in Genotype-Tissue Expression GTEX (130 genes) relates to neurogenesis and regulation of trans-synaptic signaling. (a) The gene ontology network was generated with the tools REVIGO and Cytoscape and summarizes GO-BP (Gene ontologies - Biological Process) terms found over-represented with a p-value < 0.01 in the 130 genes overlapping between UdPodocytes and genes associated with brain in GTEX. Neurogenesis and regulation of trans-synaptic signaling -related terms emerged as representative for their clusters. GOs are represented by the network nodes with light red associated with the highest significance of over-representation of a GO term. The edges refer to similarities between the GO terms. (b) Treemap of the REVIGO tool corresponding to (a). The Treemap summarizes biological process overlapping between UdPodocytes and genes associated with brain in GTEX. Representatives of the Treemap clusters include *potassium ion transport*,* neuron cell-cell adhesion*,* neurogenesis* and *regulation of trans-synaptic signaling*.

Supplementary Material 13: Figure S7 (Figure_S7_RTPCR.pdf): RT-PCR measurements of genes expressed in brain and podocytes. (A) RT-PCR measurements of genes *RPLO*,* PAX6*,* TUBB3* and *KCNQ3* expressed in brain and podocytes, 1: kidney biopsy, 2: UF21 podocyte, 3: UM27 podocyte, 4: UM48 podocyte, 5: UM51 podocyte, 6: fetal brain, 7: H_2_O. Ribosomal protein lateral stalk subunit P0 (RPL0) was used as a housekeeping gene. (B) PCR measurements of the genes *RPLO*,* PAX6*,* TUBB3* and *KCNQ3* expressed in the human immortal podocyte line (AB 8/13). The loading scheme was as follows: (1) human immortal podocyte line (AB 8/13) and (2) H_2_O. For normalization, RPL0 was used.

Supplementary Material 14: Figure S8: Protein expression from the Protein Atlas for the relevant proteins SYN1, MAPT, MAP2, BDNF, TUBB, RSPH1, SH3GL2, NPHS1, SYNPO, ATF7IP2, BACH2, EAF2, FOXD3, FOXS1, GATA4, MEIS1, SFMBT2, PSMC3IP, TFAP2B, ZNF343, ZNF367, ZNF529, ZNF665, ZNF669, ZNF682, ZNF772 and SOX2 (image credit: Human Protein Atlas, figS8_Proteinatlas_podobrain.pdf).

Supplementary Material 15: Figure S9 (figureS9.pdf): SIX2-positive UdRPCs differentiated into human podocytes. Immunofluorescence-based detection revealed that all four urine-derived podocytes and a reference immortalized podocyte cell line (AB8/13) express the podocyte-associated markers. (A) Nephrin (NPHS1). (B) α−Actinin 4 (ACTN4). Scale bars: 100 μm.

Supplementary Material 16: Figure S10 (figureS10.pdf): SIX2-positive UdRPCs are bi-potential. Immunofluorescence-based detection revealed that podocytes express Nephrin (NPHS1) and not the tubular markers- AQP1, CLCNKB and Na+-K+-ATPase as seen in the tubular differentiated cells. Secondary antibody alone was negative. Scale bars: 100 μm.

Supplementary Material 17: Figure S11: Complete images of Western Blot analyses of brain-associated proteins expressed in human podocytes (figS11_WB_uncropped.pdf).

## Data Availability

The datasets analyzed during the current study are available in the National Center for Biotechnology Information (NCBI) Gene expression Omnibus (GEO) repository, accessions GSE279611 (https://www.ncbi.nlm.nih.gov/gds/?term=GSE279611), GSE171240 (https://www.ncbi.nlm.nih.gov/gds/?term=GSE171240) and GSE186823 (https://www.ncbi.nlm.nih.gov/gds/?term=GSE186823).
