# Supplementary material for: Evidence for conserved expression of genes annotated as associated with brain-related biological processes in human podocytes and brain
Source: BMC Nephrol. 2026 Mar 4;27:230. doi: 10.1186/s12882-026-04877-2 (PMC13067571; doi:10.1186/s12882-026-04877-2)
Supplement: Supplementary file 7 — Supplementary Material 7: Figure S1 (figure S1.pdf): Light microscope pictures of human podocytes within axon-like structures. Panels A-H show human urine-derived renal progenitor cells differentiated into podocytes highlighted with black arrows their axon-like structures in their podocyte development. Scale bars: 100 μm. [file 12882_2026_4877_MOESM7_ESM.pdf]

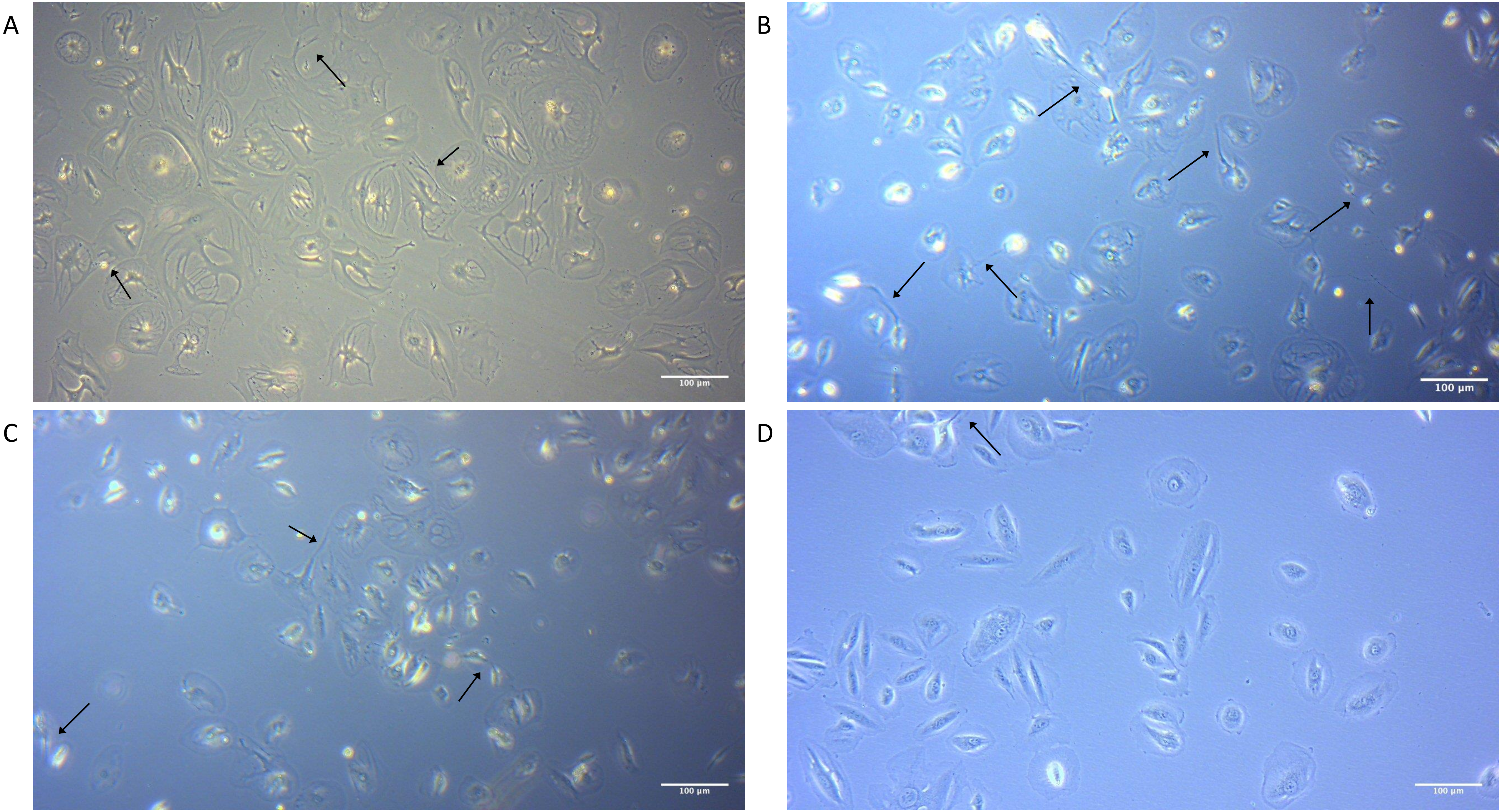
A
B
C
D
100 µm
100 µm
100 µm
100 µm

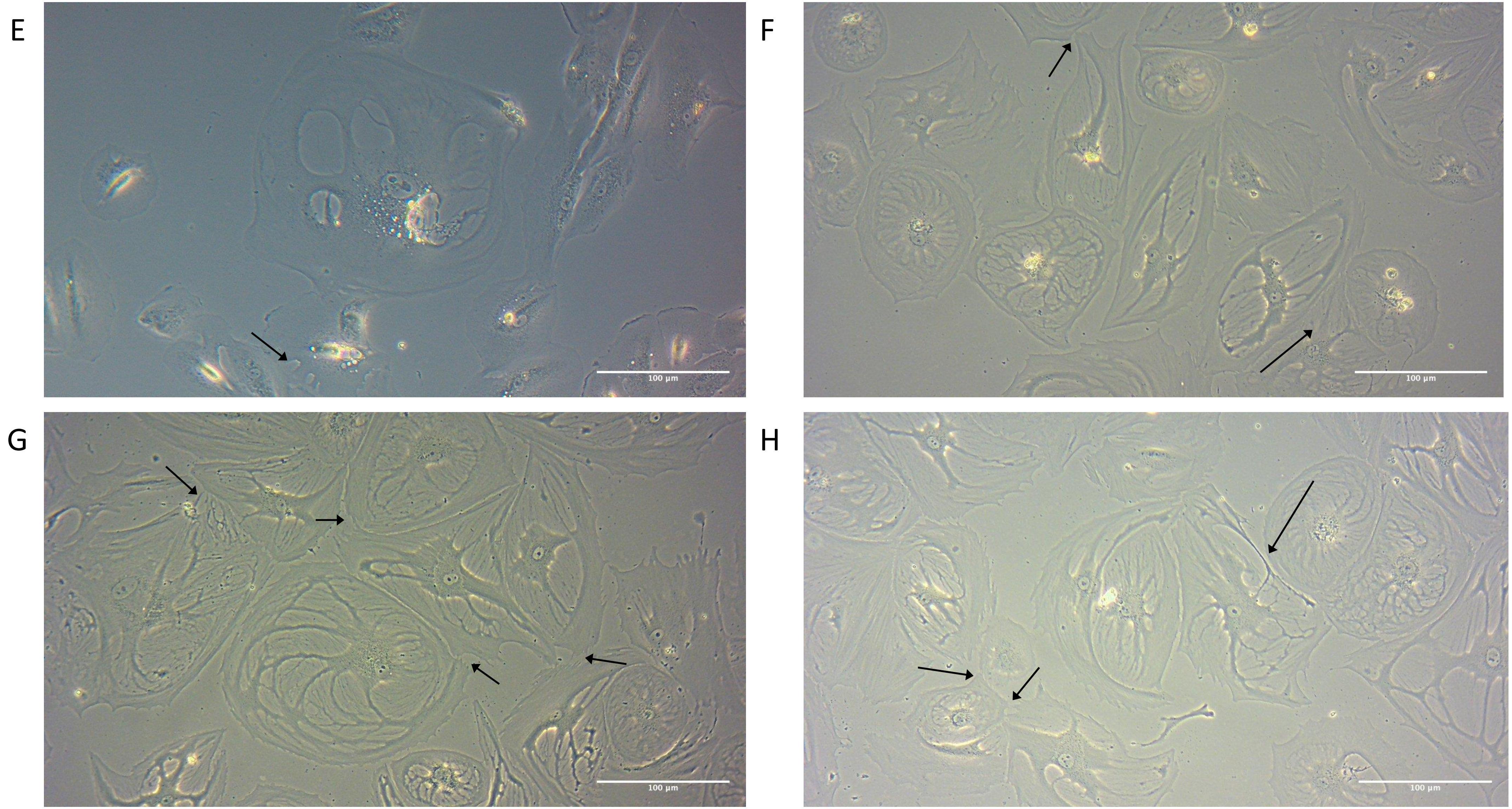


**Figure S1:** Light microscope pictures of human podocytes within axon-like structures.
Panel A-H shows human urine-derived renal progenitor cells differentiated into podocytes highlighted with black arrows their axon-like structures in their podocyte development. Scale bars: 100 µm.
