# Supplementary figures and images for: Evidence for conserved expression of genes annotated as associated with brain-related biological processes in human podocytes and brain
Source: BMC Nephrol. 2026 Mar 4;27:230. doi: 10.1186/s12882-026-04877-2 (PMC13067571; doi:10.1186/s12882-026-04877-2)

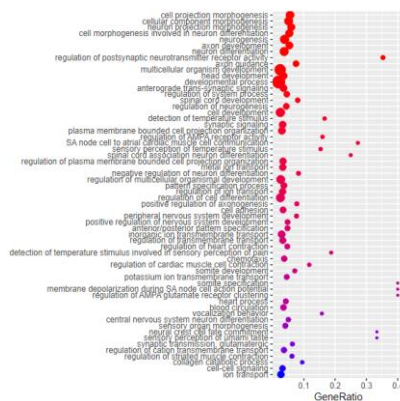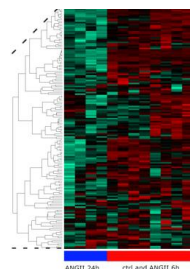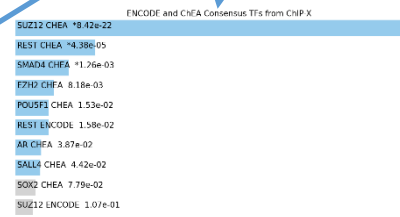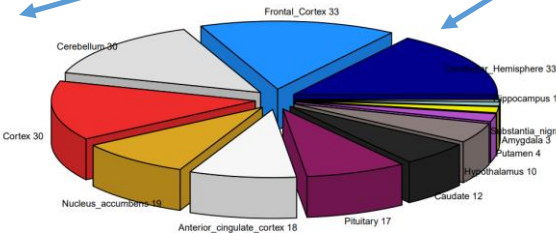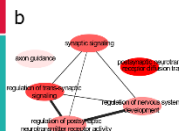

Supplement: Supplementary file 8 — Supplementary Material 8: Figure S2 (figS2_flowchart.pdf): Flow chart of the comparison between brain and podocytes. [file 12882_2026_4877_MOESM8_ESM.pdf]

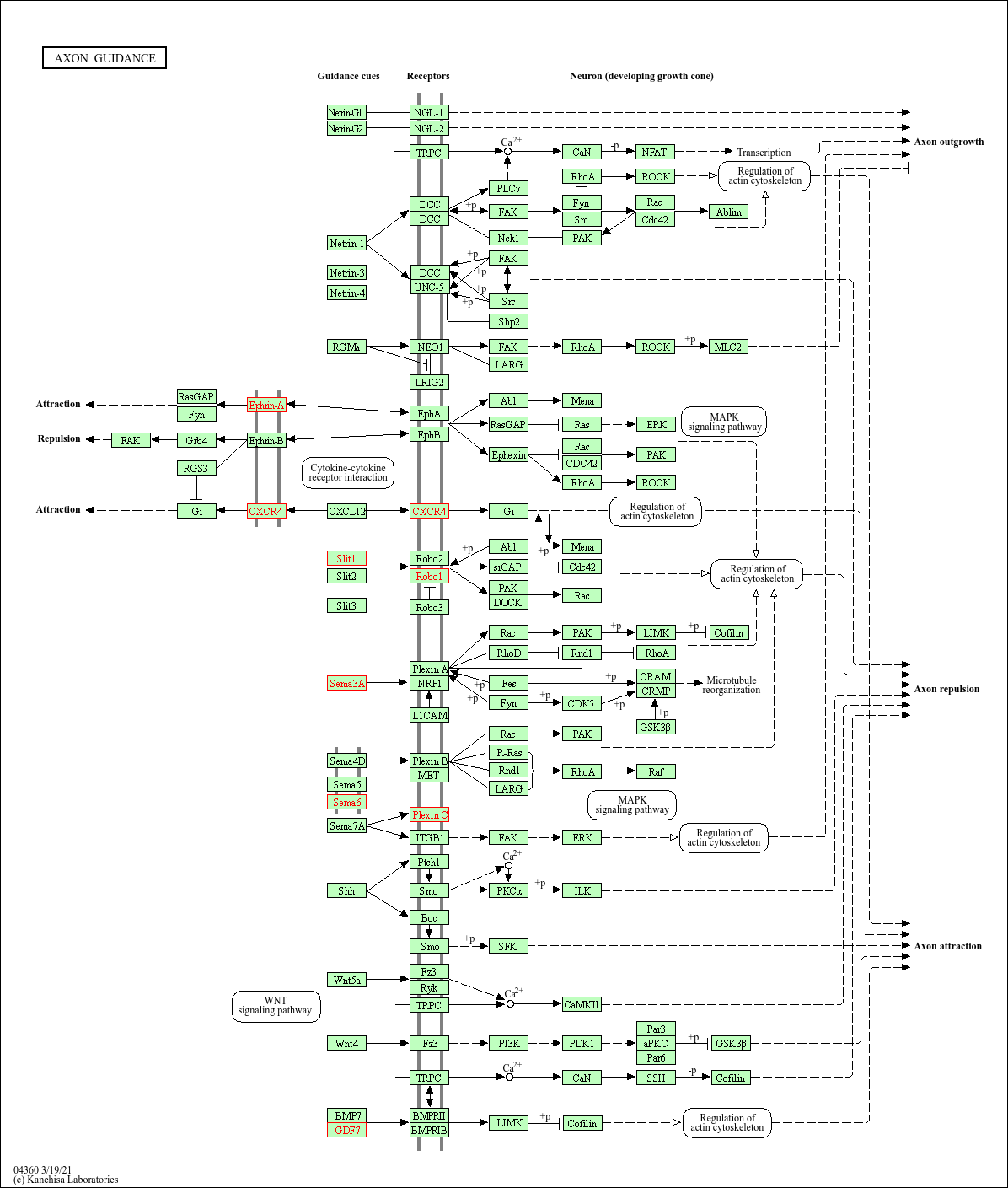

Supplement: Supplementary file 9 — Supplementary Material 9: Figure S3 (fig S3_neuro_GOs600_hsa04360_axon_guidance.png): Genes related to the GO term “axon guidance”, the most significantly over-represented biological process in the 600 genes exclusively expressed in urine-derived Podocytes (UdPodocytes), marked within the KEGG pathway chart of axon guidance (hsa04360). Several guidance factors, such as ephrins, Slits, and semaphorins are expressed in the UdPodocytes. These ligands and receptors are responsible for the regulation of the actin cytoskeleton on the one hand and on the other hand for attraction of the projection while genes responsible for repulsion are missing. Genes exclusively expressed in the UdPodocytes are marked in red. [file 12882_2026_4877_MOESM9_ESM.png]

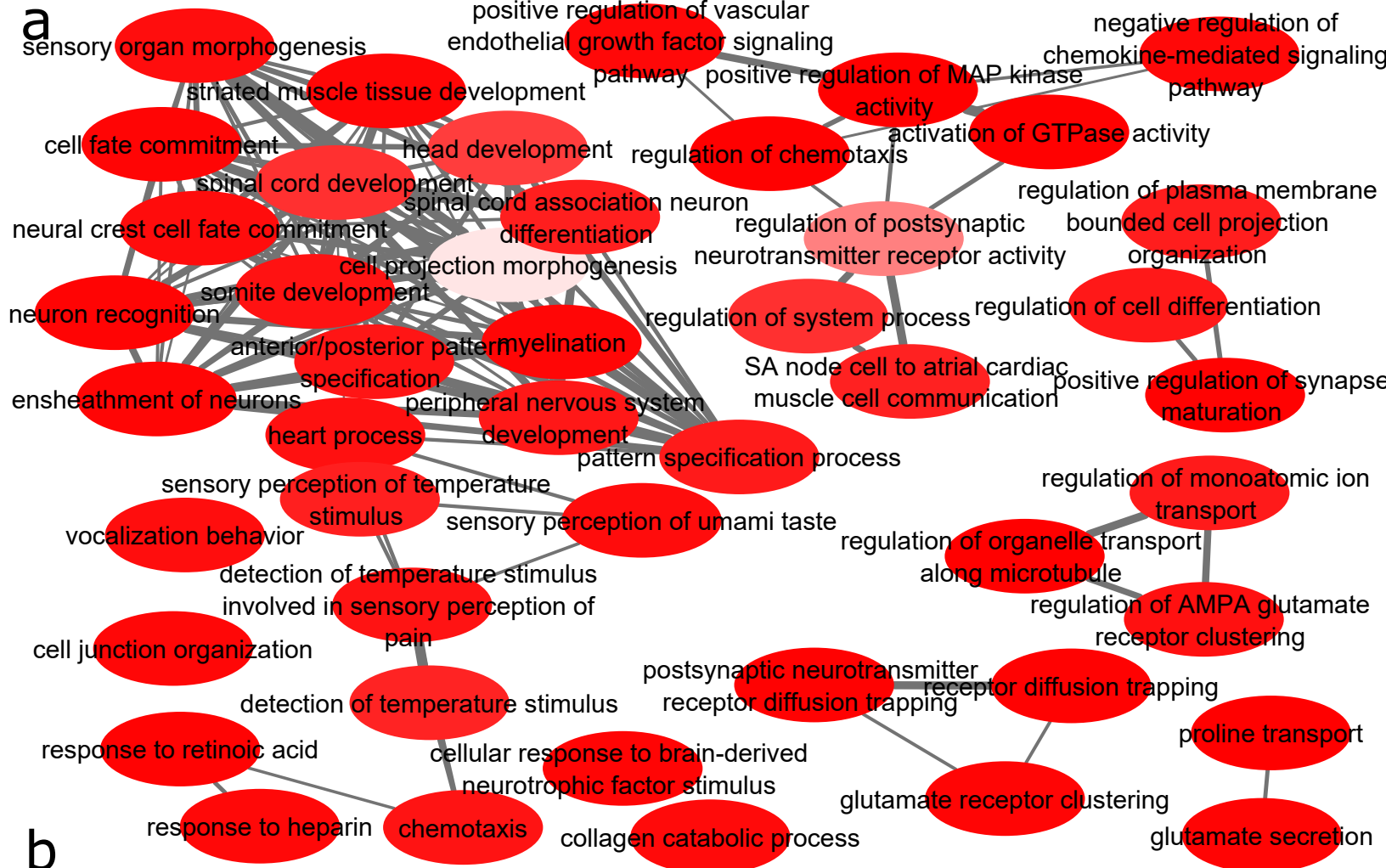

Supplement: Supplementary file 10 — Supplementary Material 10: Figure S4 (figS4.pdf): Overlap between biological processes in urine-derived Podocytes (UdPodocytes) with brain and kidney organoids (344 genes) relates to cell projection morphogenesis and regulation of postsynaptic neurotransmitter receptor activity. (a) The gene ontology network was generated with the tools REVIGO and Cytoscape and summarizes GO-BP (Gene ontologies - Biological Process) terms found over-represented with a p-value < 0.01 in the 344 genes overlapping between UdPodocytes and brain and kidney organoids. Cell projection morphogenesis and regulation of postsynaptic neurotransmitter receptor activity -related terms emerged as representative for their clusters. GOs are represented by the network nodes with light red associated with the highest significance of over-representation of a GO term. The edges refer to similarities between the GO terms. (b) Treemap of the REVIGO tool corresponding to (a). The Treemap summarizes biological process overlapping between UdPodocytes and brain and kidney organoids. Representatives of the Treemap clusters include cell adhesion, detection of temperature stimulus, metal ion transport, collagen catabolic process, developmental process, cell projection morphogenesis, actin-mediated cell contraction and regulation of postsynaptic neurotransmitter receptor activity. [file 12882_2026_4877_MOESM10_ESM.pdf]

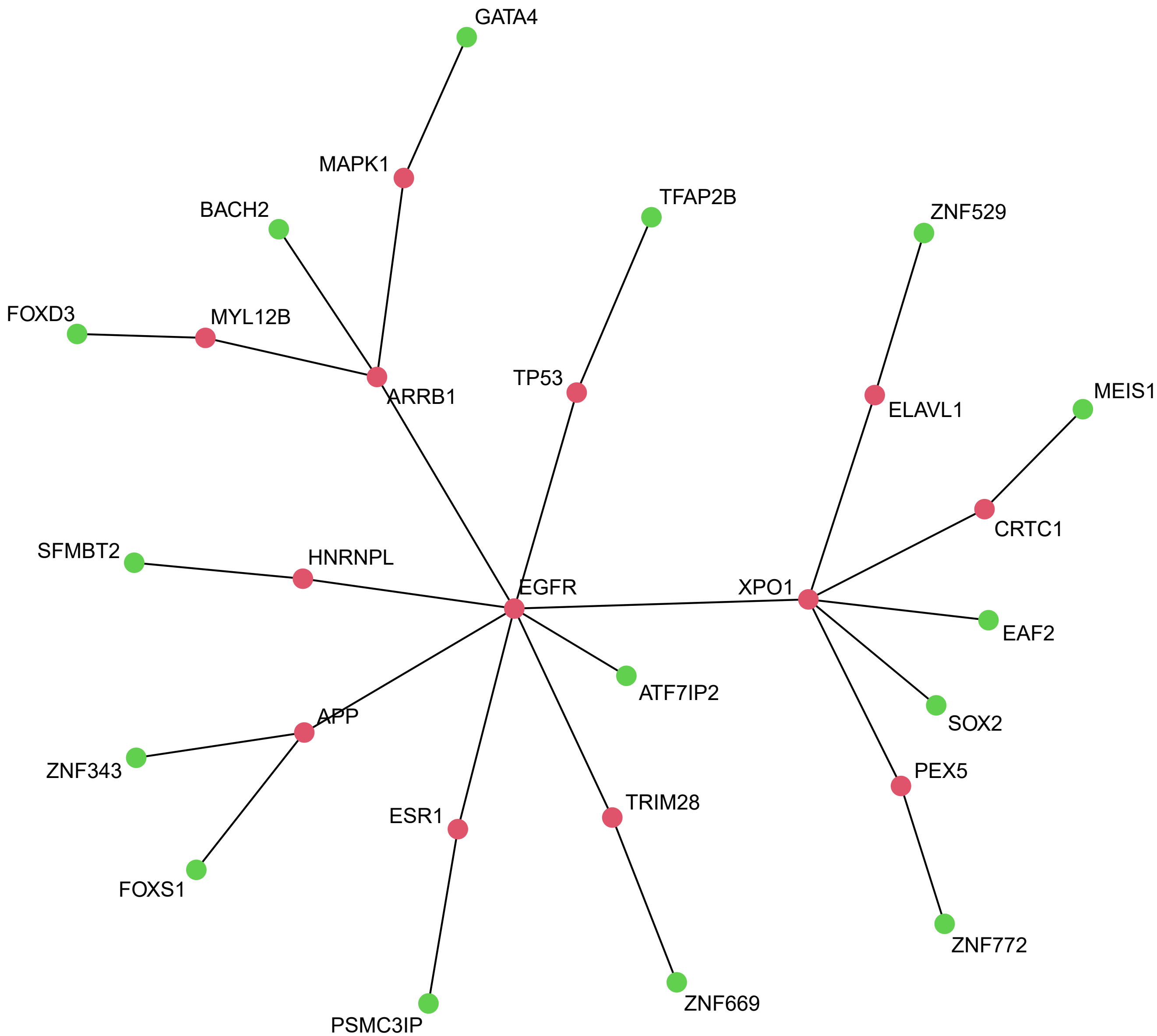

Supplement: Supplementary file 11 — Supplementary Material 11: Figure S5 (figS5_net_TFs_reduced_opt.pdf): Protein interaction network of Proteinatlas-validated Transcription Factors (TFs) amongst the 344 genes subset expressed in common in UdPodocytes and brain and kidney organoids. The TFs validated for expression in the kidney via the Proteinatlas could be connected in a protein interaction network of Biogrid interactions using EGFR and XPO1 as major hubs. Green nodes are the original TFs and red nodes were added via Biogrid interactions. [file 12882_2026_4877_MOESM11_ESM.pdf]

a

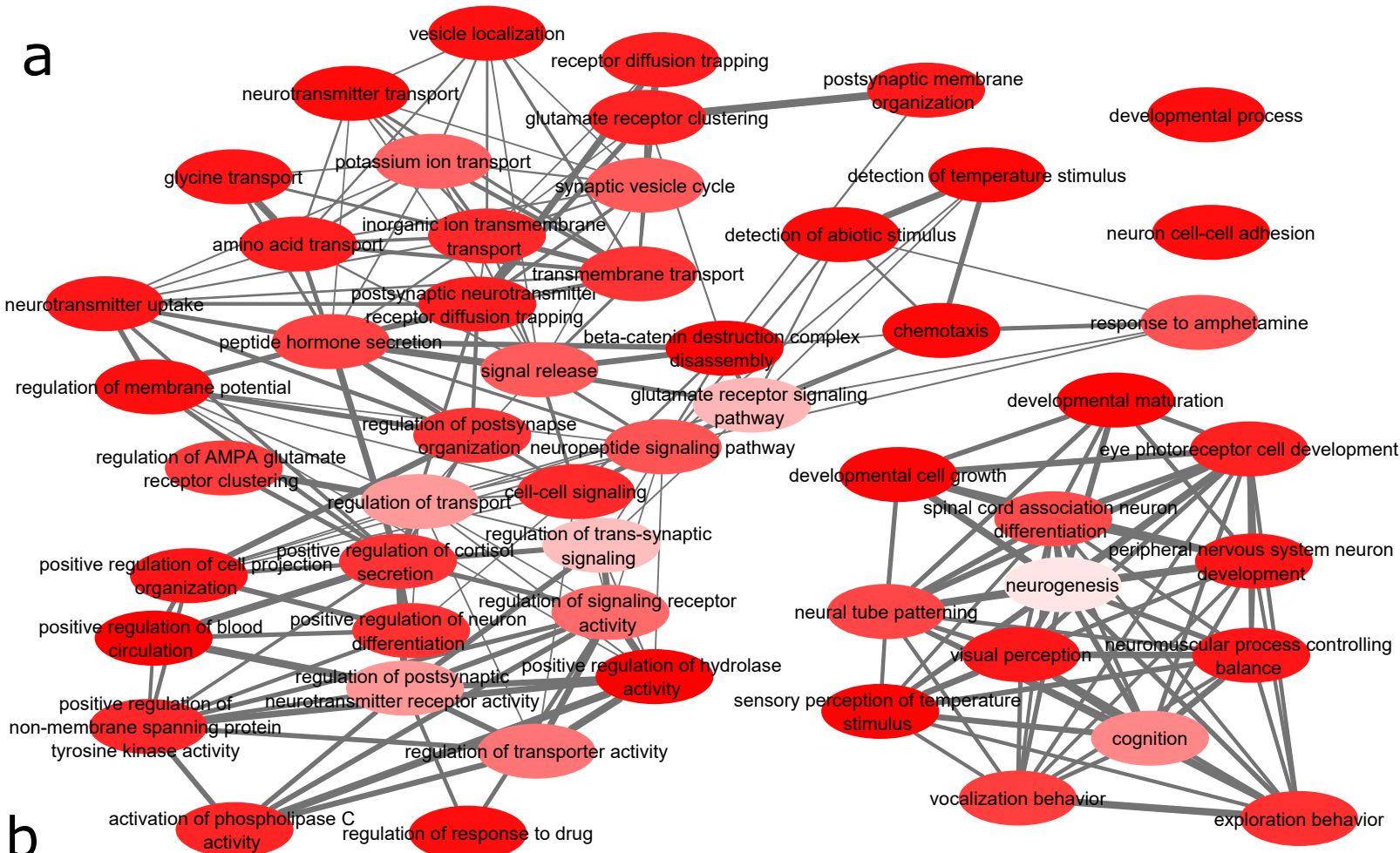

b

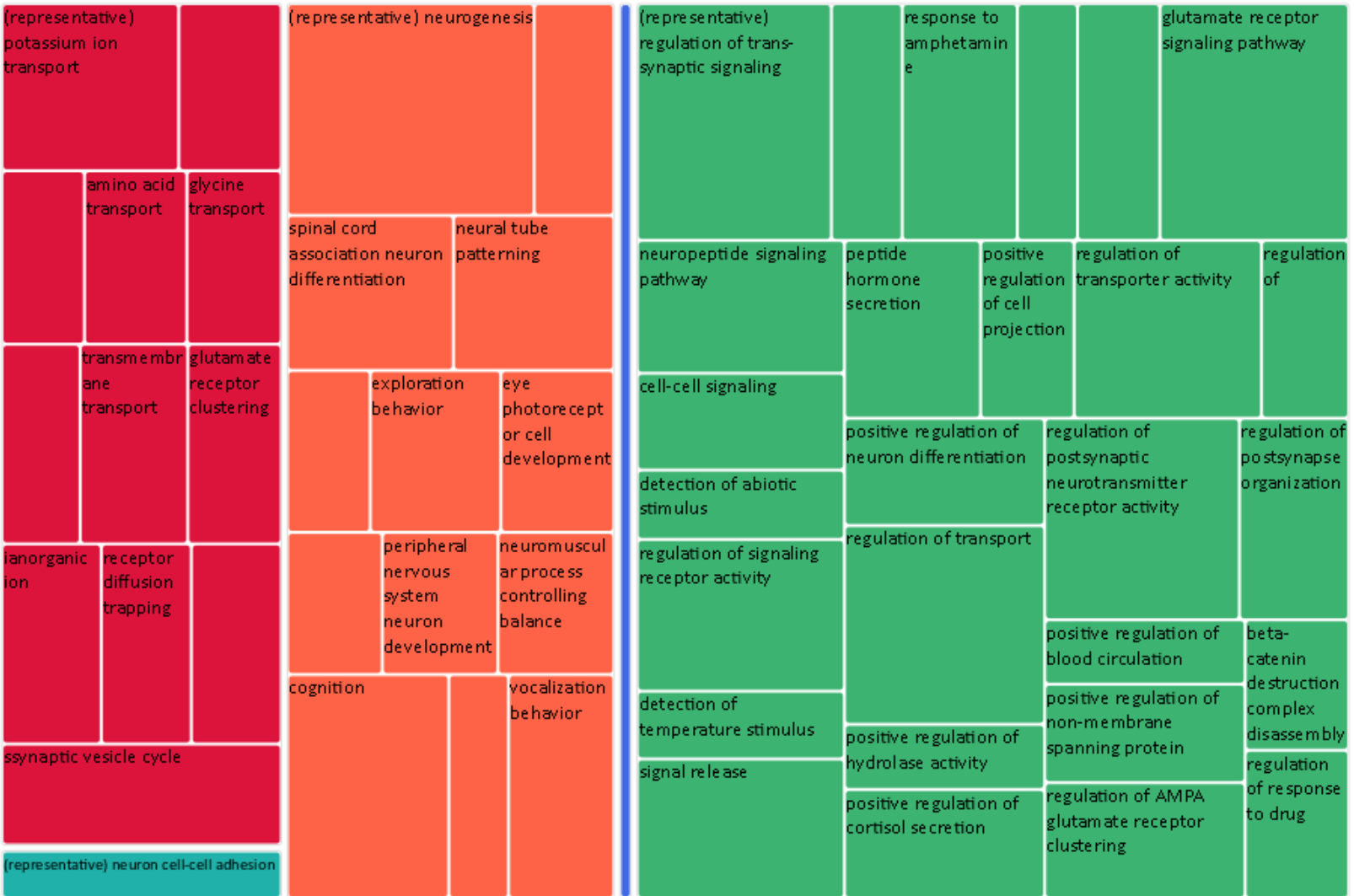

Supplement: Supplementary file 12 — Supplementary Material 12: Figure S6 (figS6.pdf): Overlap between biological processes in urine-derived Podocytes (UdPodocytes) with genes associated with brain in Genotype-Tissue Expression GTEX (130 genes) relates to neurogenesis and regulation of trans-synaptic signaling. (a) The gene ontology network was generated with the tools REVIGO and Cytoscape and summarizes GO-BP (Gene ontologies - Biological Process) terms found over-represented with a p-value < 0.01 in the 130 genes overlapping between UdPodocytes and genes associated with brain in GTEX. Neurogenesis and regulation of trans-synaptic signaling -related terms emerged as representative for their clusters. GOs are represented by the network nodes with light red associated with the highest significance of over-representation of a GO term. The edges refer to similarities between the GO terms. (b) Treemap of the REVIGO tool corresponding to (a). The Treemap summarizes biological process overlapping between UdPodocytes and genes associated with brain in GTEX. Representatives of the Treemap clusters include potassium ion transport, neuron cell-cell adhesion, neurogenesis and regulation of trans-synaptic signaling. [file 12882_2026_4877_MOESM12_ESM.pdf]
