# Supplementary material for: Evidence for conserved expression of genes annotated as associated with brain-related biological processes in human podocytes and brain
Source: BMC Nephrol. 2026 Mar 4;27:230. doi: 10.1186/s12882-026-04877-2 (PMC13067571; doi:10.1186/s12882-026-04877-2)
Supplement: Supplementary file 13 — Supplementary Material 13: Figure S7 (Figure_S7_RTPCR.pdf): RT-PCR measurements of genes expressed in brain and podocytes. (A) RT-PCR measurements of genes RPLO, PAX6, TUBB3 and KCNQ3 expressed in brain and podocytes, 1: kidney biopsy, 2: UF21 podocyte, 3: UM27 podocyte, 4: UM48 podocyte, 5: UM51 podocyte, 6: fetal brain, 7: H2O. Ribosomal protein lateral stalk subunit P0 (RPL0) was used as a housekeeping gene. (B) PCR measurements of the genes RPLO, PAX6, TUBB3 and KCNQ3 expressed in the human immortal podocyte line (AB 8/13). The loading scheme was as follows: (1) human immortal podocyte line (AB 8/13) and (2) H2O. For normalization, RPL0 was used. [file 12882_2026_4877_MOESM13_ESM.pdf]

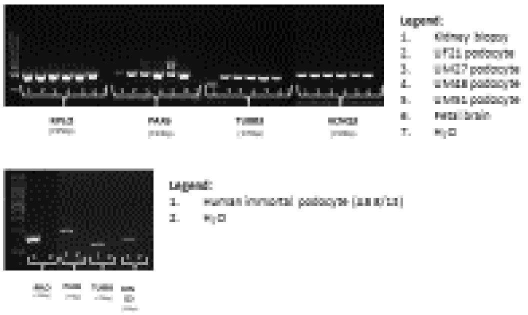

Figure S7: RT-PCR measurements of genes expressed in brain and podocytes.

(A) RT-PCR measurements of genes *RPLO, PAX6, TUBB3* and *KCNQ3* expressed in brain and podocytes, 1: kidney biopsy, 2: UF21 podocyte, 3: UM27 podocyte, 4: UM48 podocyte, 5: UM51 podocyte, 6: fetal brain, 7: H_2O. Ribosomal protein lateral stalk subunit P0 (RPL0) was used as a housekeeping gene. (**B**) PCR measurements of the genes *RPLO, PAX6, TUBB3* and *KCNQ3* expressed in the human immortal podocyte line (AB 8/13). The loading scheme was as follows: 1. human immortal podocyte line (AB 8/13) and 2. H_2O. For normalization, RPL0 was used.
