# Supplementary material for: Evidence for conserved expression of genes annotated as associated with brain-related biological processes in human podocytes and brain
Source: BMC Nephrol. 2026 Mar 4;27:230. doi: 10.1186/s12882-026-04877-2 (PMC13067571; doi:10.1186/s12882-026-04877-2)
Supplement: Supplementary file 14 — Supplementary Material 14: Figure S8: Protein expression from the Protein Atlas for the relevant proteins SYN1, MAPT, MAP2, BDNF, TUBB, RSPH1, SH3GL2, NPHS1, SYNPO, ATF7IP2, BACH2, EAF2, FOXD3, FOXS1, GATA4, MEIS1, SFMBT2, PSMC3IP, TFAP2B, ZNF343, ZNF367, ZNF529, ZNF665, ZNF669, ZNF682, ZNF772 and SOX2 (image credit: Human Protein Atlas, figS8_Proteinatlas_podobrain.pdf). [file 12882_2026_4877_MOESM14_ESM.pdf]

Proteinatlas

Brain related genes

Synapsin1

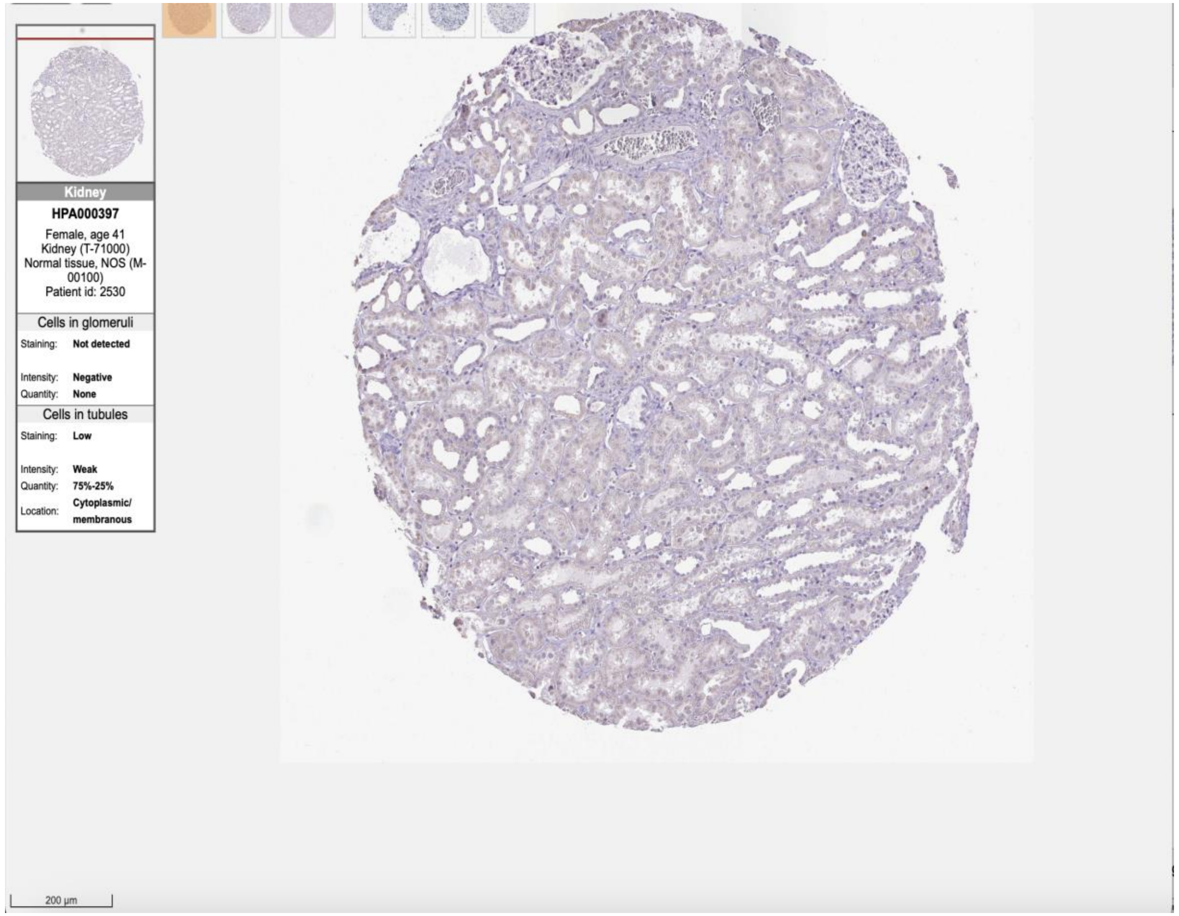

Kidney
HPA000397
Female, age 41
Kidney (T-71000)
Normal tissue, NOS (M-00100)
Patient id: 2530
Cells in glomeruli
Staining: Not detected
Intensity: Negative
Quantity: None
Cells in tubules
Staining: Low
Intensity: Weak
Quantity: 75%-25%
Location: Cytoplasmic/ membranous
200 µm

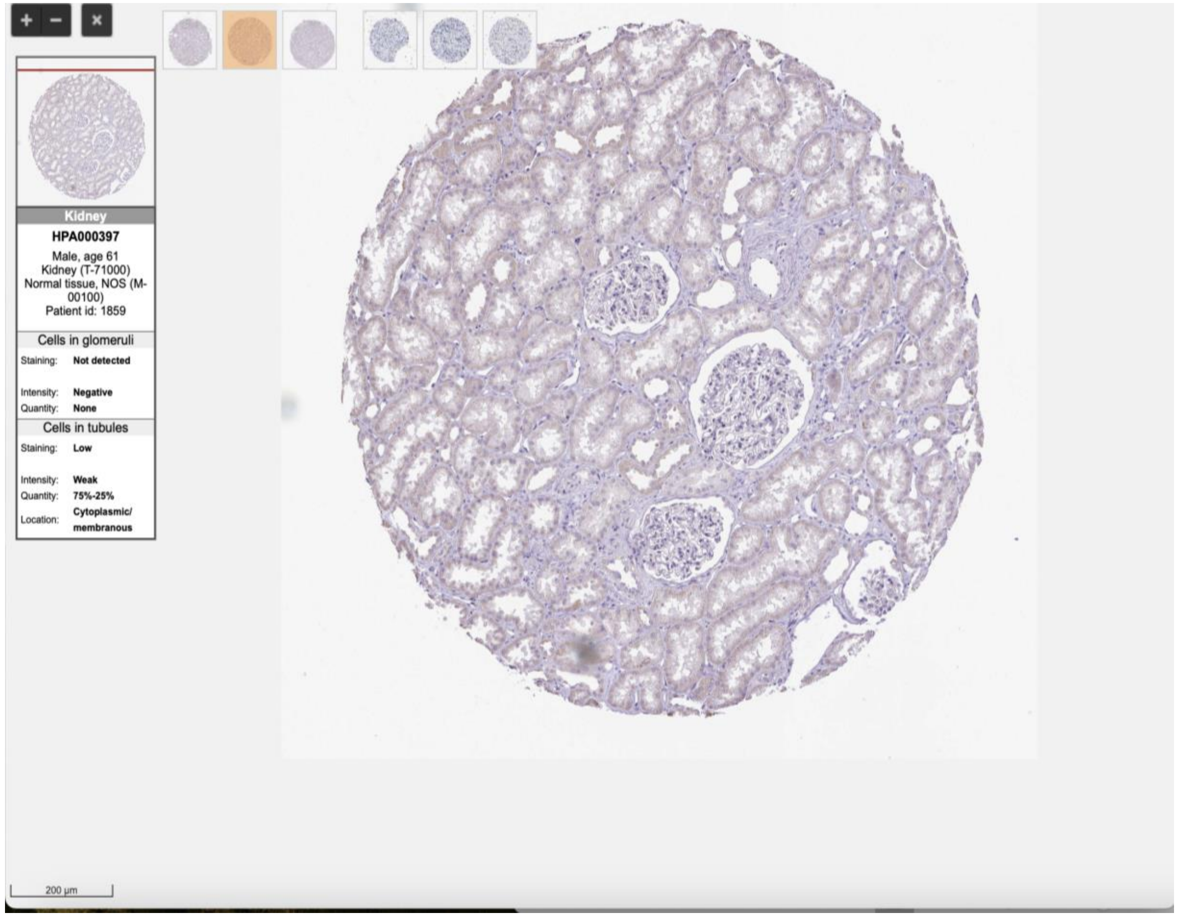

Kidney
HPA000397
Male, age 61
Kidney (T-71000)
Normal tissue, NOS (M-00100)
Patient id: 1859
Cells in glomeruli
Staining: Not detected
Intensity: Negative
Quantity: None
Cells in tubules
Staining: Low
Intensity: Weak
Quantity: 75%-25%
Location: Cytoplasmic/ membranous
200 µm

MAPT

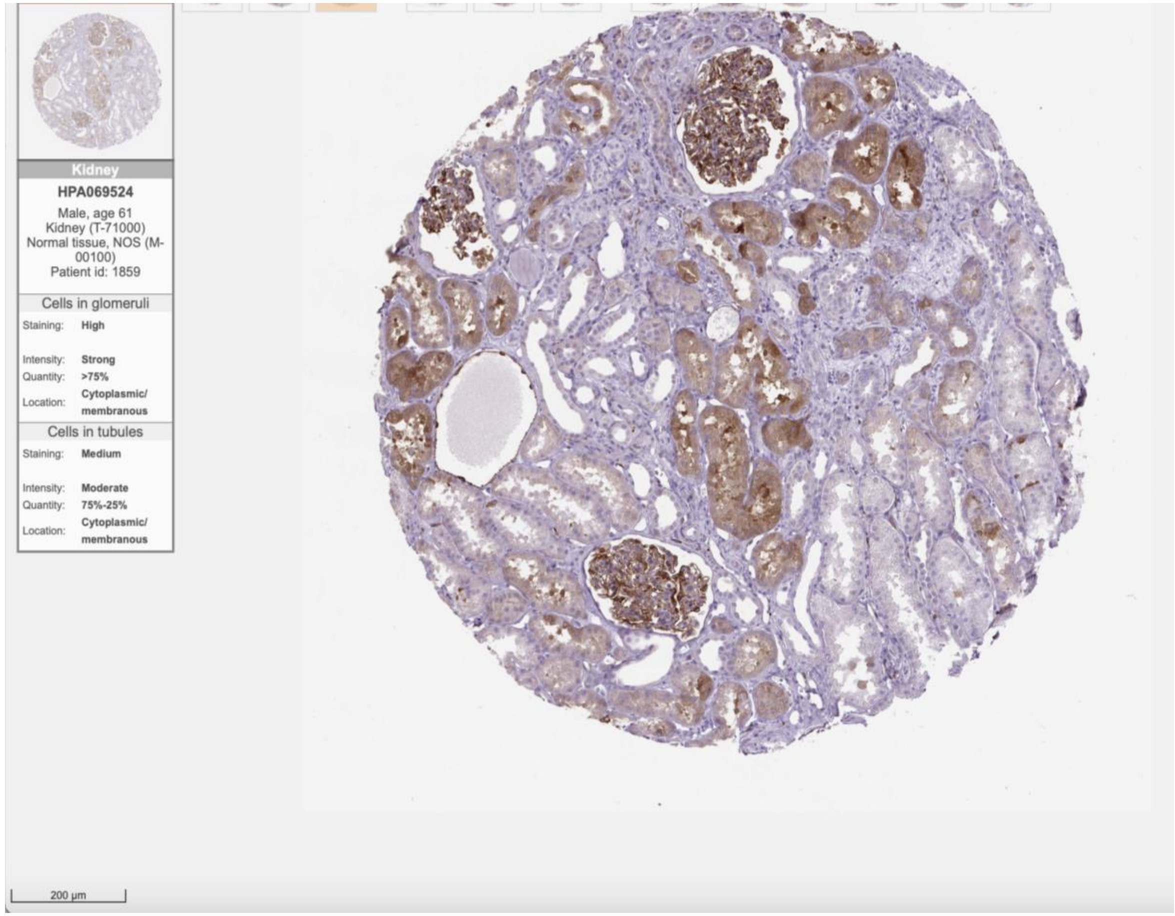
Kidney
HPA069524
Male, age 61
Kidney (T-71000)
Normal tissue, NOS (M-00100)
Patient id: 1859
Cells in glomeruli
Staining: High
Intensity: Strong
Quantity: >75%
Location: Cytoplasmic/ membranous
Cells in tubules
Staining: Medium
Intensity: Moderate
Quantity: 75%-25%
Location: Cytoplasmic/ membranous
200 µm

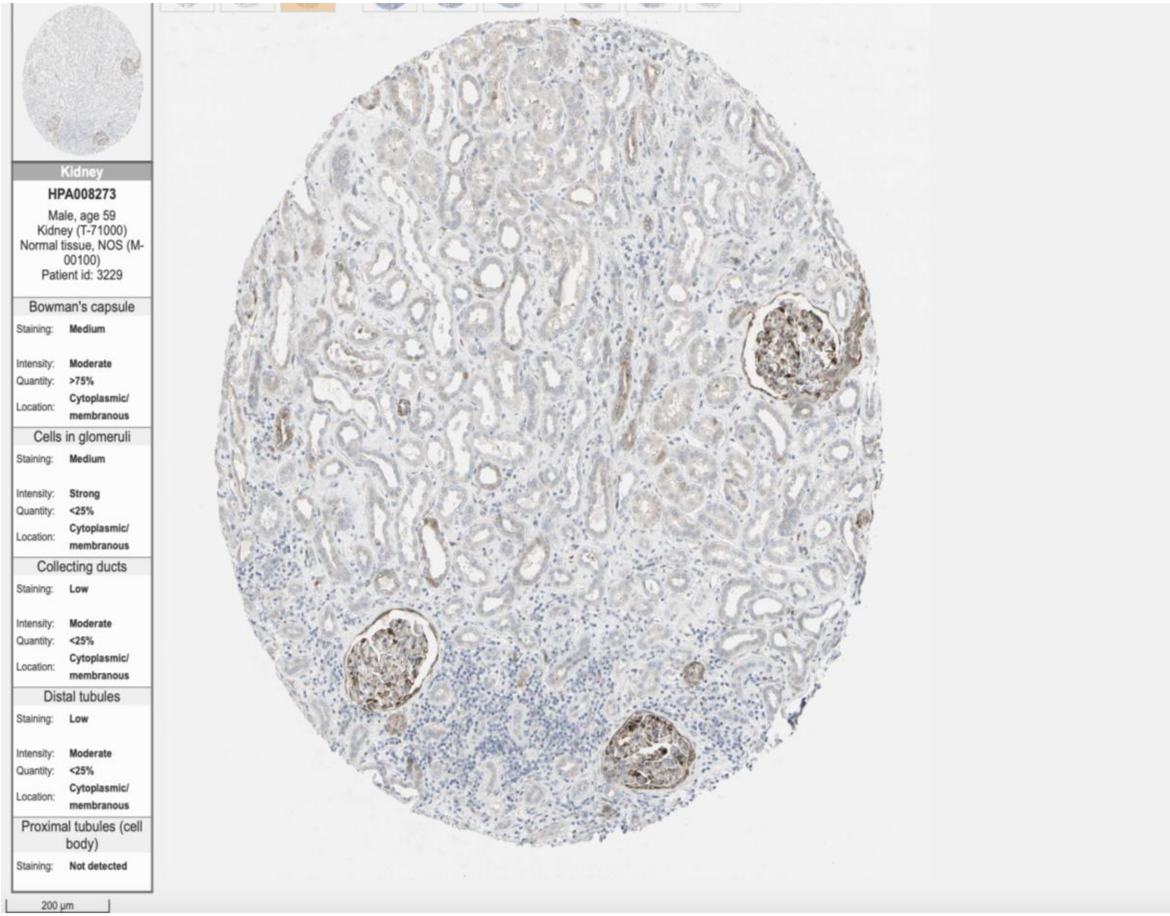
Kidney
HPA008273
Male, age 59
Kidney (T-71000)
Normal tissue, NOS (M-00100)
Patient id: 3229
Bowman's capsule
Staining: Medium
Intensity: Moderate
Quantity: >75%
Location: Cytoplasmic/ membranous
Cells in glomeruli
Staining: Medium
Intensity: Strong
Quantity: <25%
Location: Cytoplasmic/ membranous
Collecting ducts
Staining: Low
Intensity: Moderate
Quantity: <25%
Location: Cytoplasmic/ membranous
Distal tubules
Staining: Low
Intensity: Moderate
Quantity: <25%
Location: Cytoplasmic/ membranous
Proximal tubules (cell body)
Staining: Not detected
200 µm

MAP2

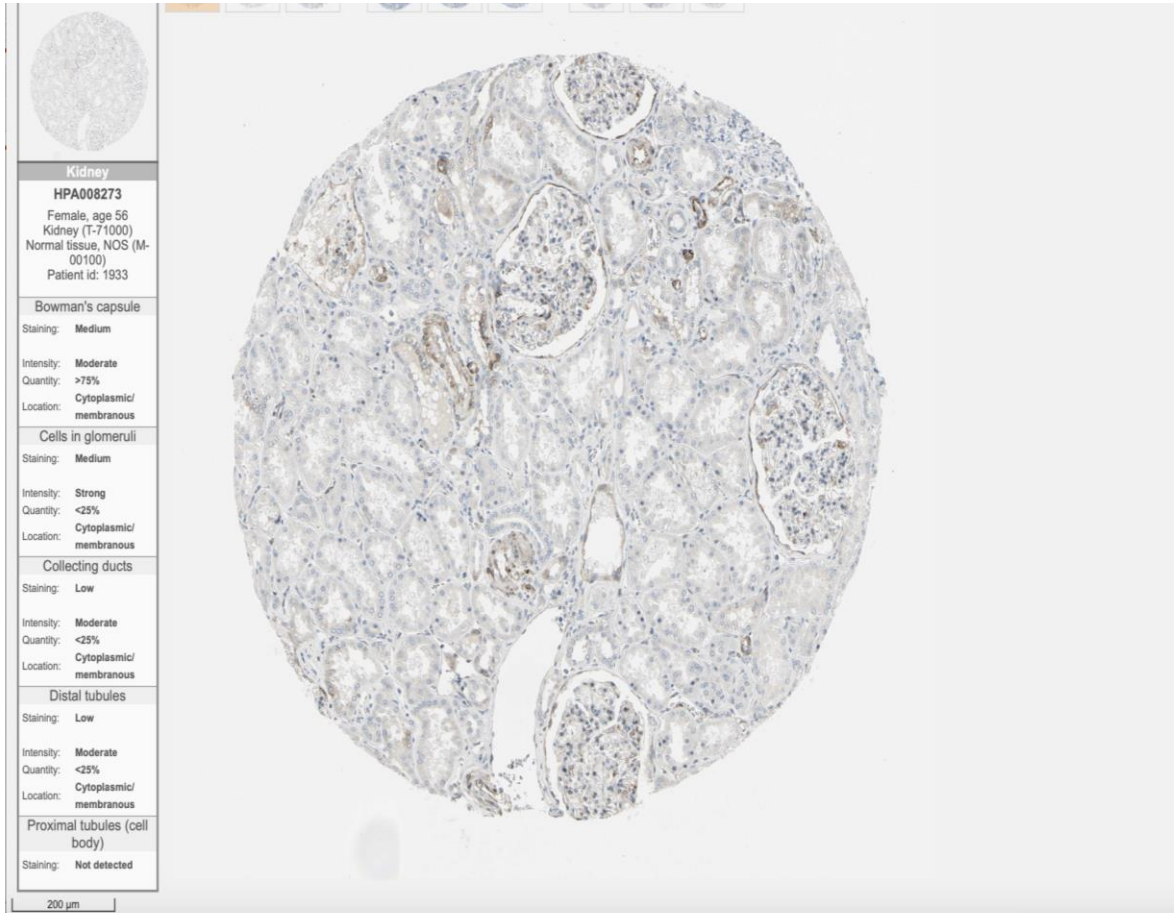
Kidney
HPA008273
Female, age 56
Kidney (T-71000)
Normal tissue, NOS (M-00100)
Patient id: 1933
Bowman's capsule
Staining: Medium
Intensity: Moderate
Quantity: >75%
Location: Cytoplasmic/membranous
Cells in glomeruli
Staining: Medium
Intensity: Strong
Quantity: <25%
Location: Cytoplasmic/membranous
Collecting ducts
Staining: Low
Intensity: Moderate
Quantity: <25%
Location: Cytoplasmic/membranous
Distal tubules
Staining: Low
Intensity: Moderate
Quantity: <25%
Location: Cytoplasmic/membranous
Proximal tubules (cell body)
Staining: Not detected
200 µm

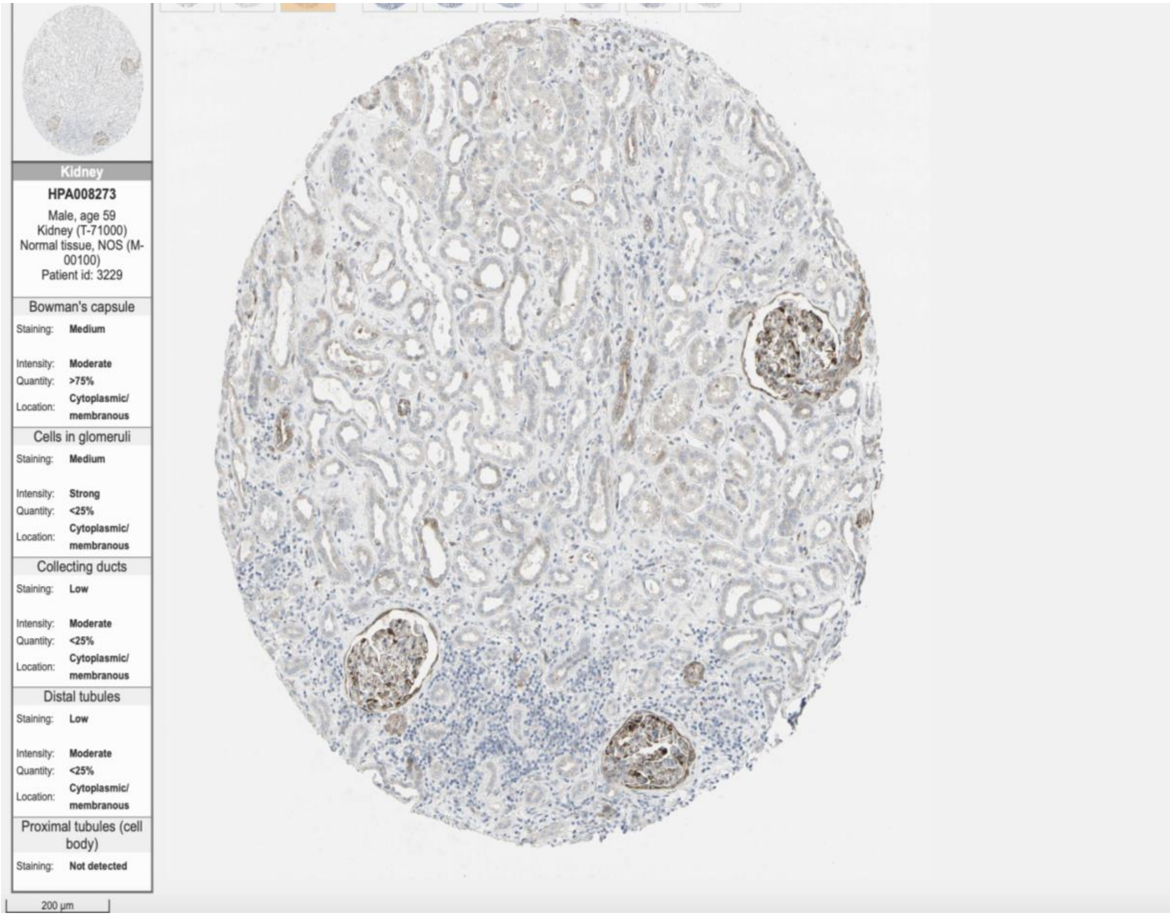
Kidney
HPA008273
Male, age 59
Kidney (T-71000)
Normal tissue, NOS (M-00100)
Patient id: 3229
Bowman's capsule
Staining: Medium
Intensity: Moderate
Quantity: >75%
Location: Cytoplasmic/membranous
Cells in glomeruli
Staining: Medium
Intensity: Strong
Quantity: <25%
Location: Cytoplasmic/membranous
Collecting ducts
Staining: Low
Intensity: Moderate
Quantity: <25%
Location: Cytoplasmic/membranous
Distal tubules
Staining: Low
Intensity: Moderate
Quantity: <25%
Location: Cytoplasmic/membranous
Proximal tubules (cell body)
Staining: Not detected
200 µm

BDNF

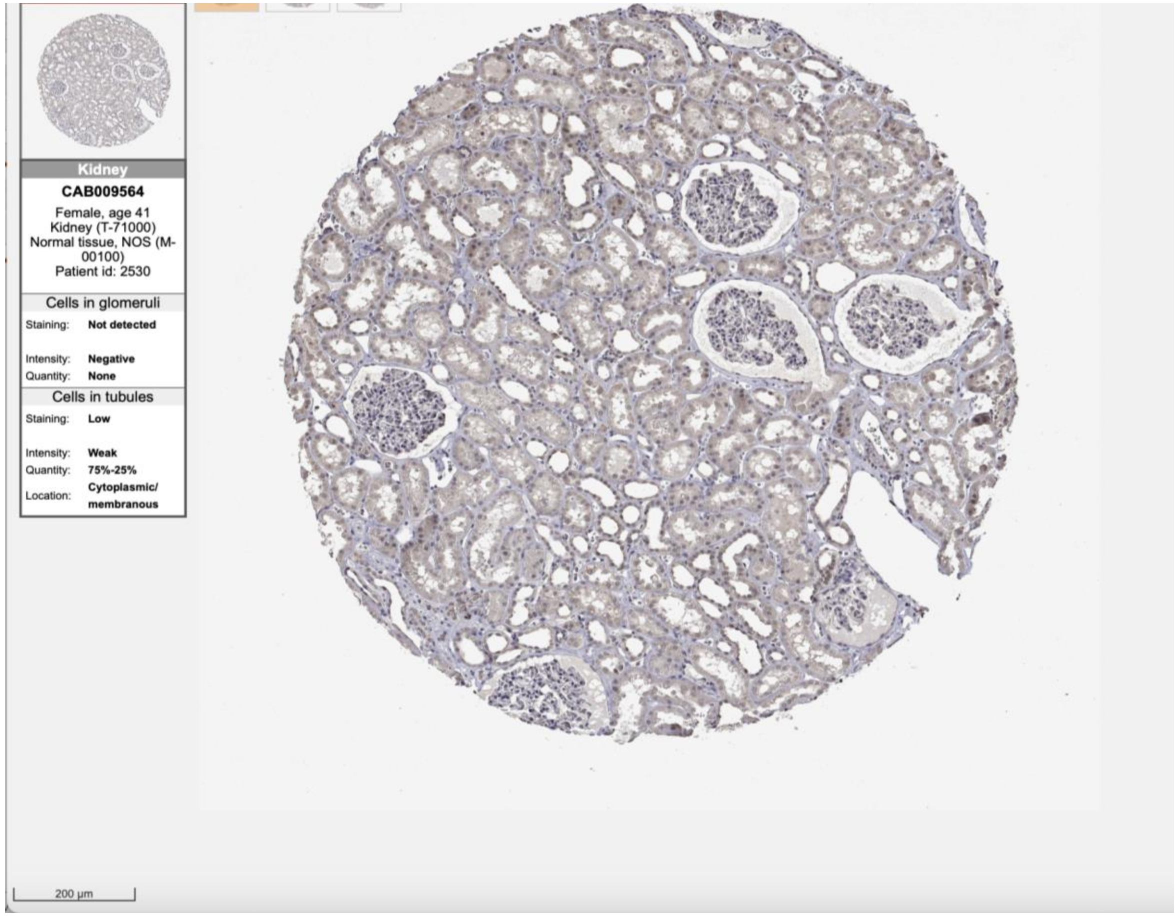
Kidney
CAB009564
Female, age 41
Kidney (T-71000)
Normal tissue, NOS (M-00100)
Patient id: 2530
Cells in glomeruli
Staining: Not detected
Intensity: Negative
Quantity: None
Cells in tubules
Staining: Low
Intensity: Weak
Quantity: 75%-25%
Location: Cytoplasmic/ membranous
200 µm

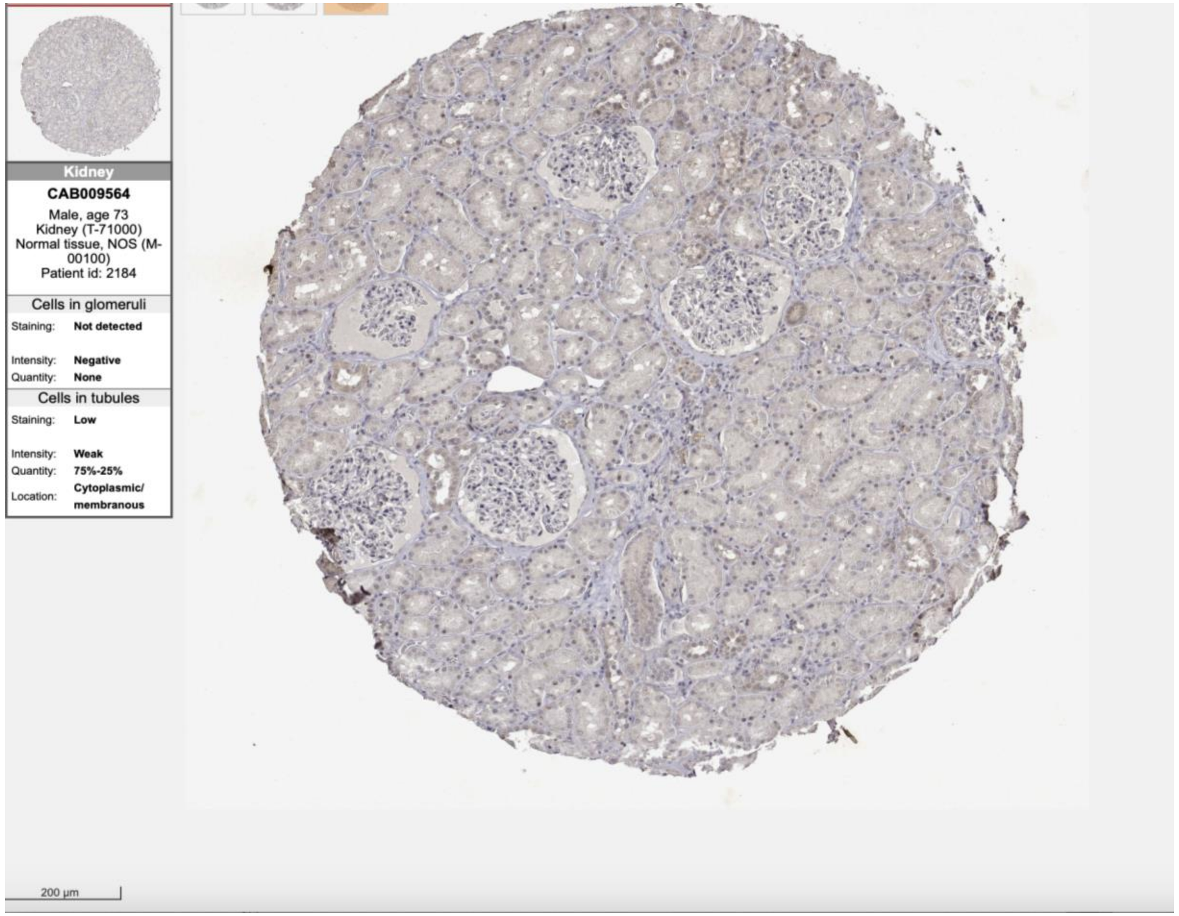
Kidney
CAB009564
Male, age 73
Kidney (T-71000)
Normal tissue, NOS (M-00100)
Patient id: 2184
Cells in glomeruli
Staining: Not detected
Intensity: Negative
Quantity: None
Cells in tubules
Staining: Low
Intensity: Weak
Quantity: 75%-25%
Location: Cytoplasmic/ membranous
200 µm

TUBB

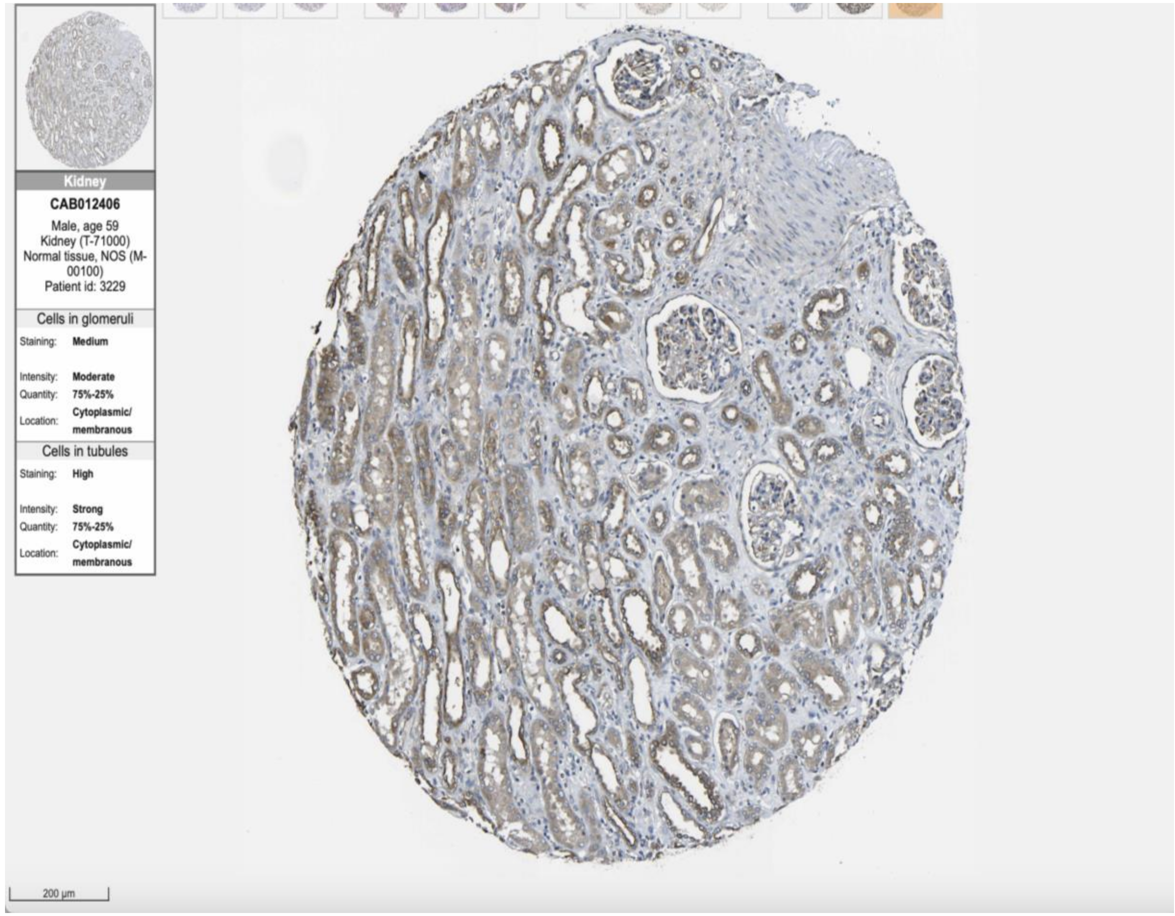
Kidney
CAB012406
Male, age 59
Kidney (T-71000)
Normal tissue, NOS (M-00100)
Patient id: 3229
Cells in glomeruli
Staining: Medium
Intensity: Moderate
Quantity: 75%-25%
Location: Cytoplasmic/ membranous
Cells in tubules
Staining: High
Intensity: Strong
Quantity: 75%-25%
Location: Cytoplasmic/ membranous
200 µm

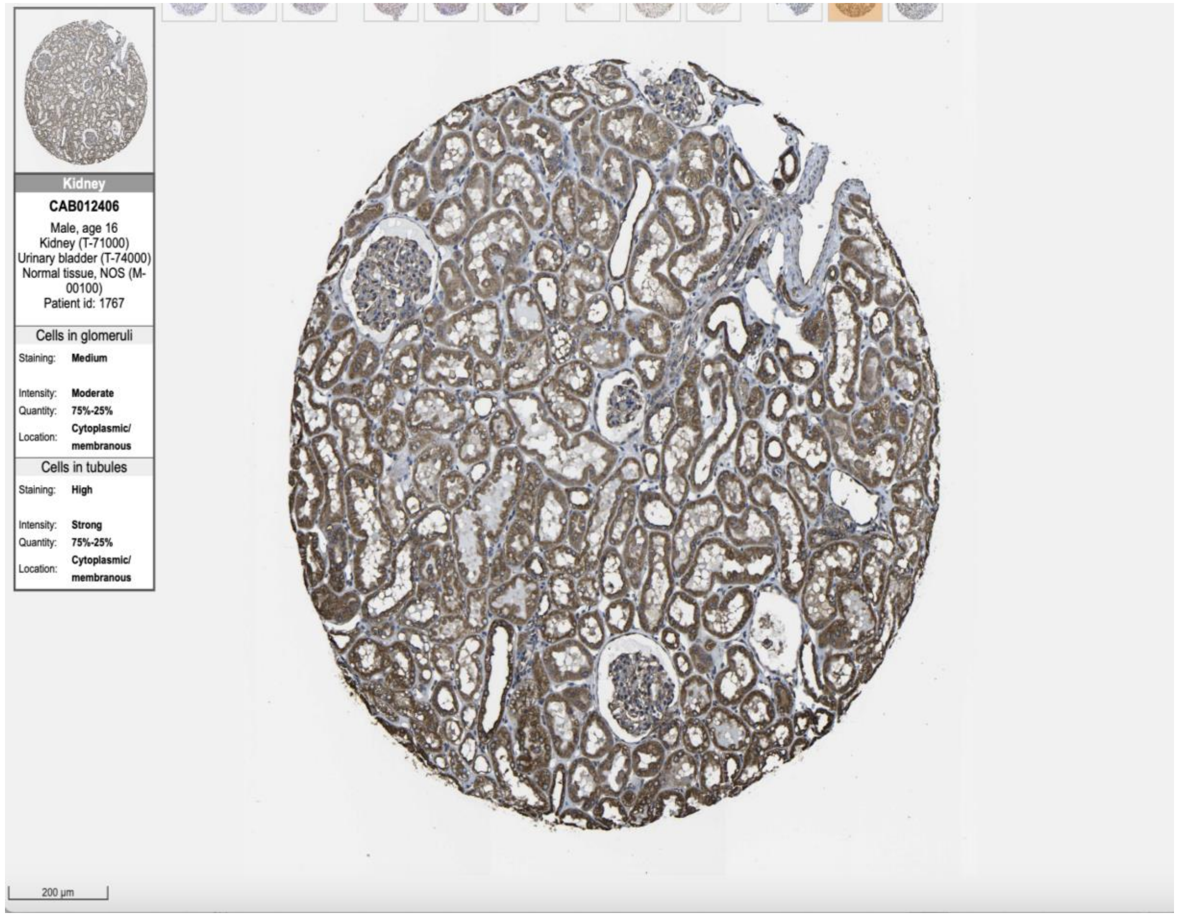
Kidney
CAB012406
Male, age 16
Kidney (T-71000)
Urinary bladder (T-74000)
Normal tissue, NOS (M-00100)
Patient id: 1767
Cells in glomeruli
Staining: Medium
Intensity: Moderate
Quantity: 75%-25%
Location: Cytoplasmic/ membranous
Cells in tubules
Staining: High
Intensity: Strong
Quantity: 75%-25%
Location: Cytoplasmic/ membranous
200 µm

RSPH1

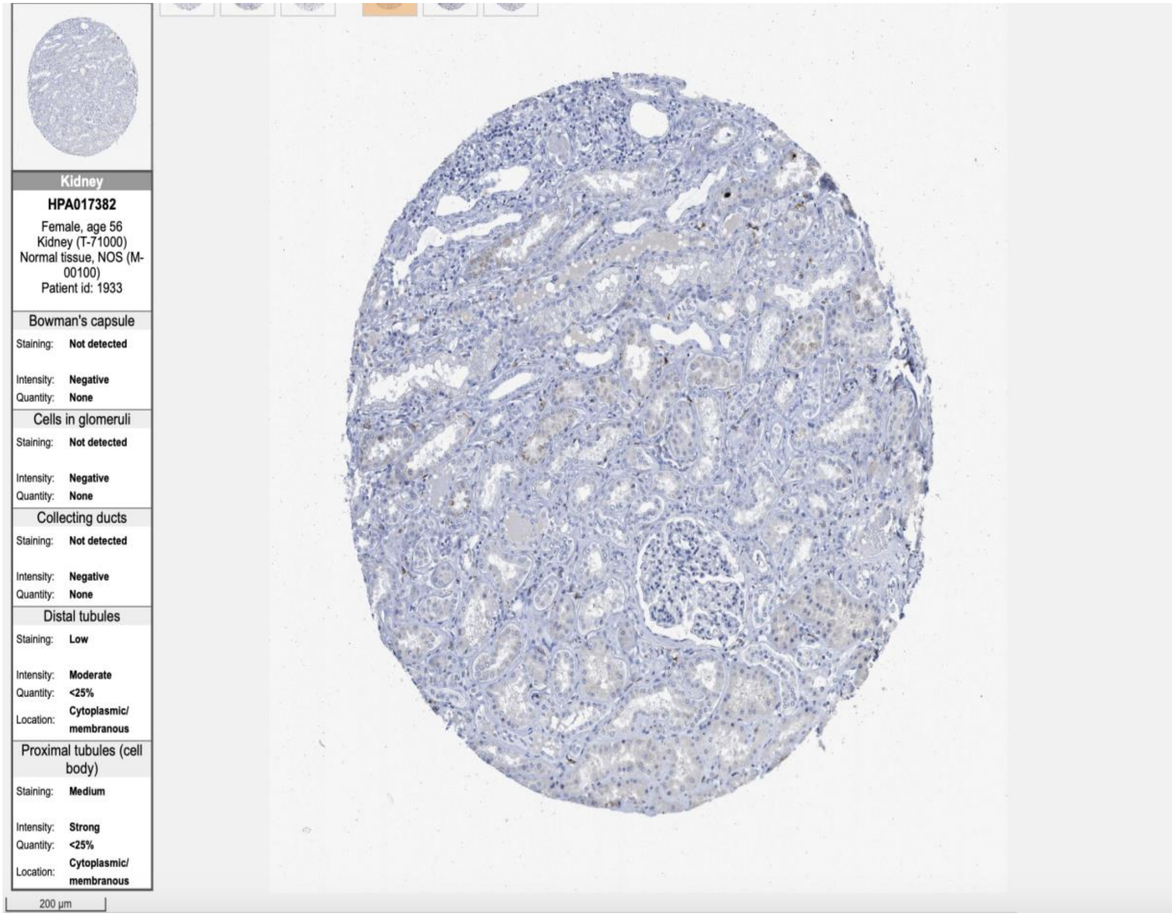
Kidney
HPA017382
Female, age 56
Kidney (T-71000)
Normal tissue, NOS (M-00100)
Patient id: 1933
Bowman's capsule
Staining: Not detected
Intensity: Negative
Quantity: None
Cells in glomeruli
Staining: Not detected
Intensity: Negative
Quantity: None
Collecting ducts
Staining: Not detected
Intensity: Negative
Quantity: None
Distal tubules
Staining: Low
Intensity: Moderate
Quantity: <25%
Location: Cytoplasmic/ membranous
Proximal tubules (cell body)
Staining: Medium
Intensity: Strong
Quantity: <25%
Location: Cytoplasmic/ membranous
200 µm

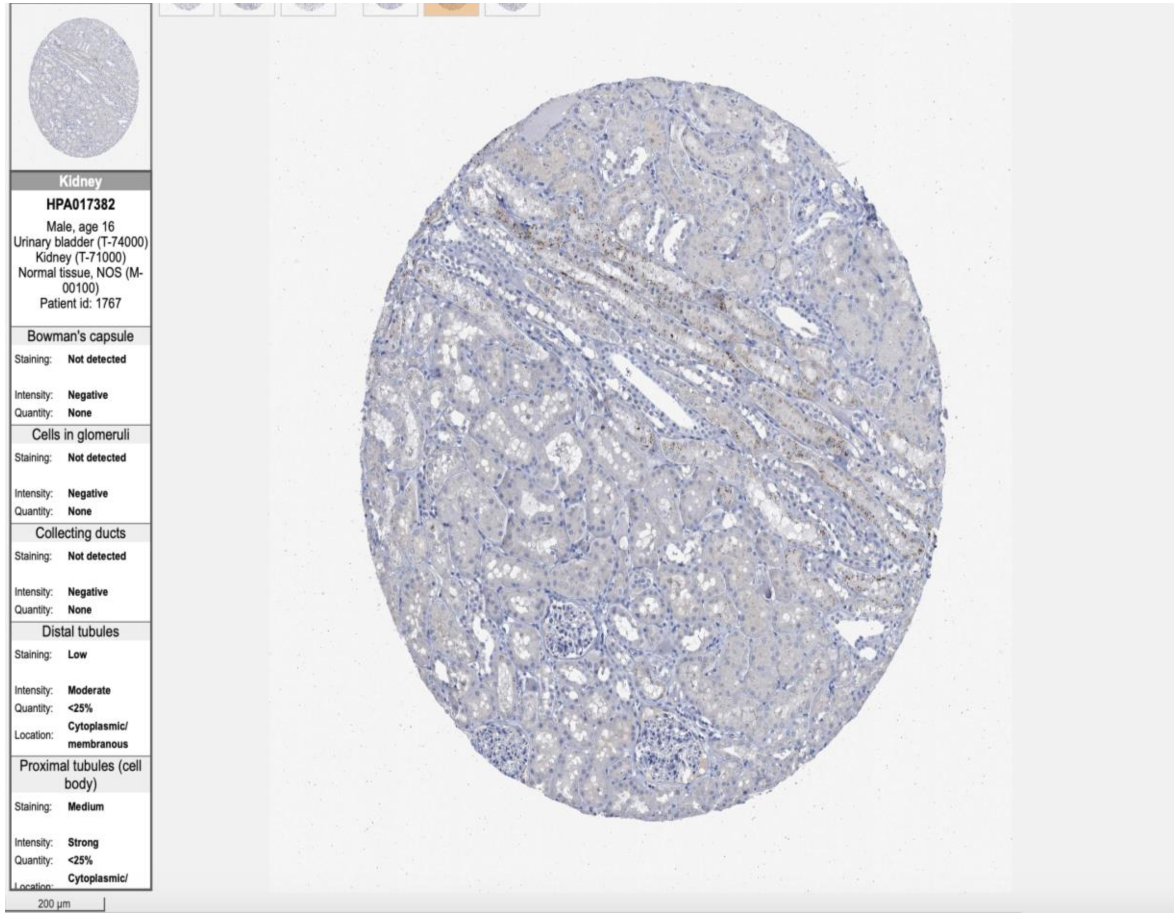
Kidney
HPA017382
Male, age 16
Urinary bladder (T-74000)
Kidney (T-71000)
Normal tissue, NOS (M-00100)
Patient id: 1767
Bowman's capsule
Staining: Not detected
Intensity: Negative
Quantity: None
Cells in glomeruli
Staining: Not detected
Intensity: Negative
Quantity: None
Collecting ducts
Staining: Not detected
Intensity: Negative
Quantity: None
Distal tubules
Staining: Low
Intensity: Moderate
Quantity: <25%
Location: Cytoplasmic/ membranous
Proximal tubules (cell body)
Staining: Medium
Intensity: Strong
Quantity: <25%
Cytoplasmic/
200 µm

SH3GL2

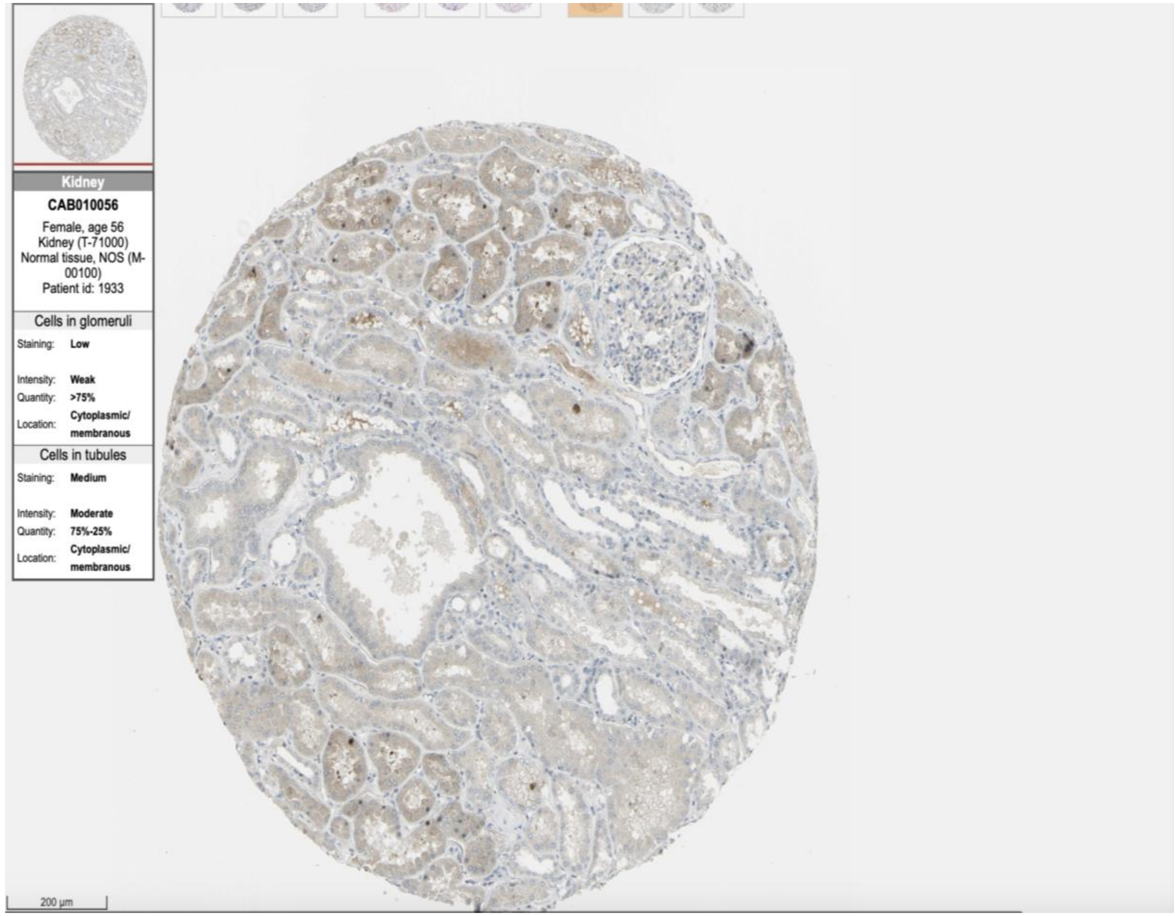
Kidney
CAB010056
Female, age 56
Kidney (T-71000)
Normal tissue, NOS (M-00100)
Patient id: 1933
Cells in glomeruli
Staining: Low
Intensity: Weak
Quantity: >75%
Location: Cytoplasmic/membranous
Cells in tubules
Staining: Medium
Intensity: Moderate
Quantity: 75%-25%
Location: Cytoplasmic/membranous
200 µm

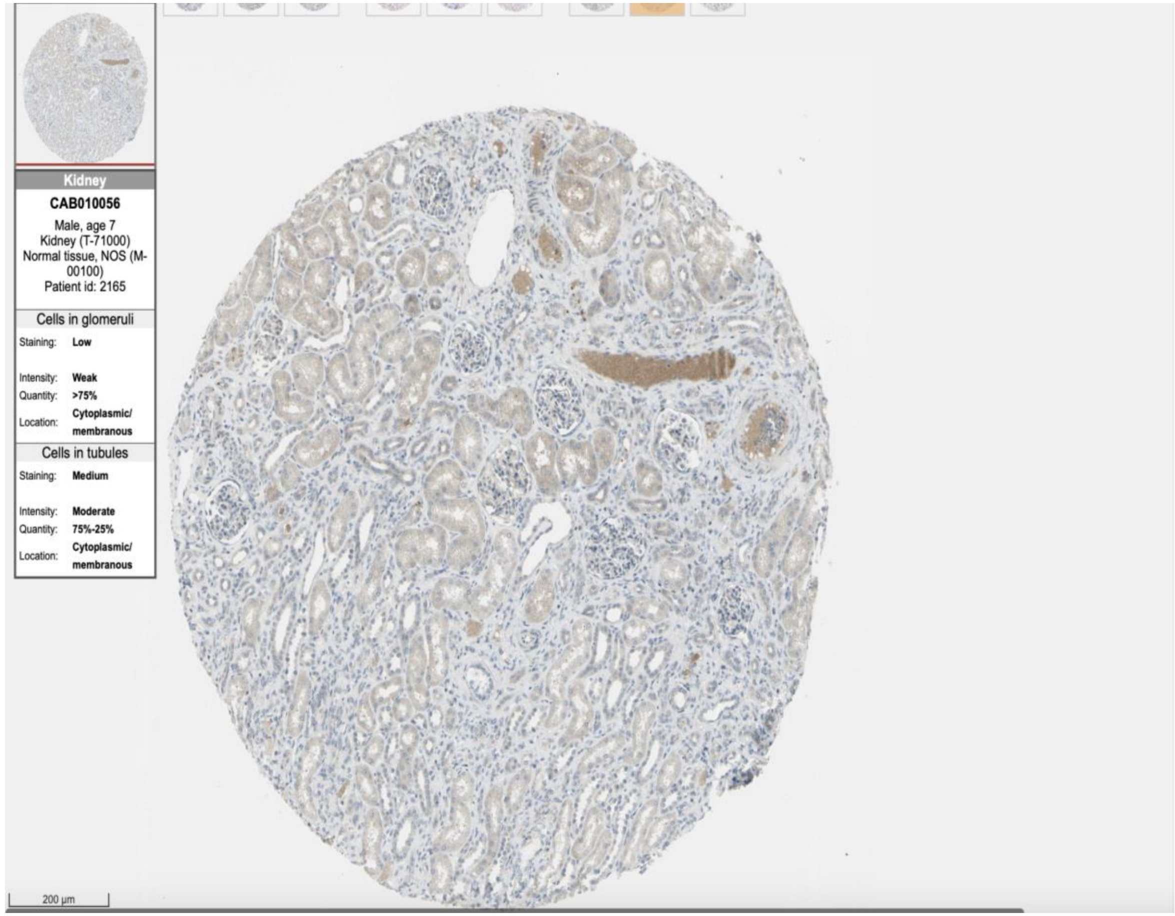
Kidney
CAB010056
Male, age 7
Kidney (T-71000)
Normal tissue, NOS (M-00100)
Patient id: 2165
Cells in glomeruli
Staining: Low
Intensity: Weak
Quantity: >75%
Location: Cytoplasmic/membranous
Cells in tubules
Staining: Medium
Intensity: Moderate
Quantity: 75%-25%
Location: Cytoplasmic/membranous
200 µm

NPHS1

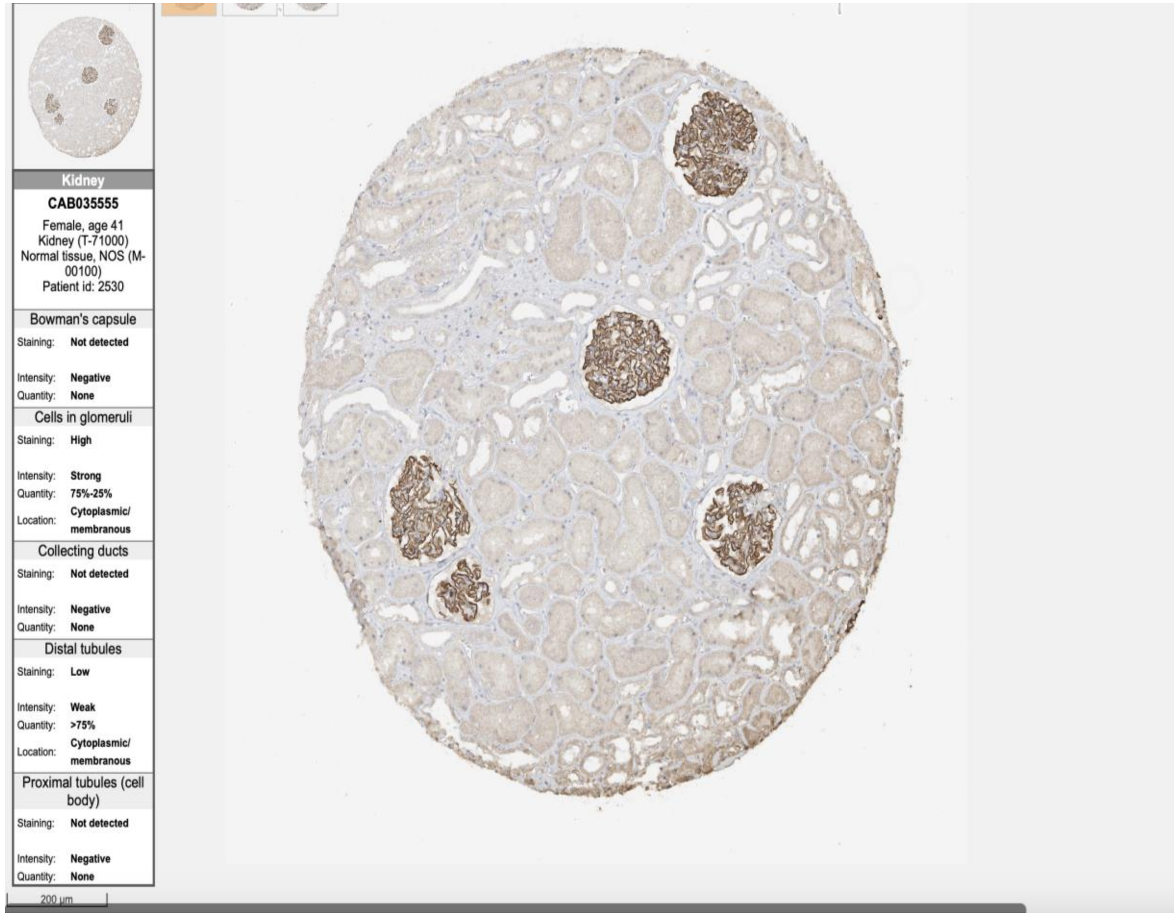
Kidney
CAB035555
Female, age 41
Kidney (T-71000)
Normal tissue, NOS (M-00100)
Patient id: 2530
Bowman's capsule
Staining: Not detected
Intensity: Negative
Quantity: None
Cells in glomeruli
Staining: High
Intensity: Strong
Quantity: 75%-25%
Location: Cytoplasmic/ membranous
Collecting ducts
Staining: Not detected
Intensity: Negative
Quantity: None
Distal tubules
Staining: Low
Intensity: Weak
Quantity: >75%
Location: Cytoplasmic/ membranous
Proximal tubules (cell body)
Staining: Not detected
Intensity: Negative
Quantity: None
200 µm

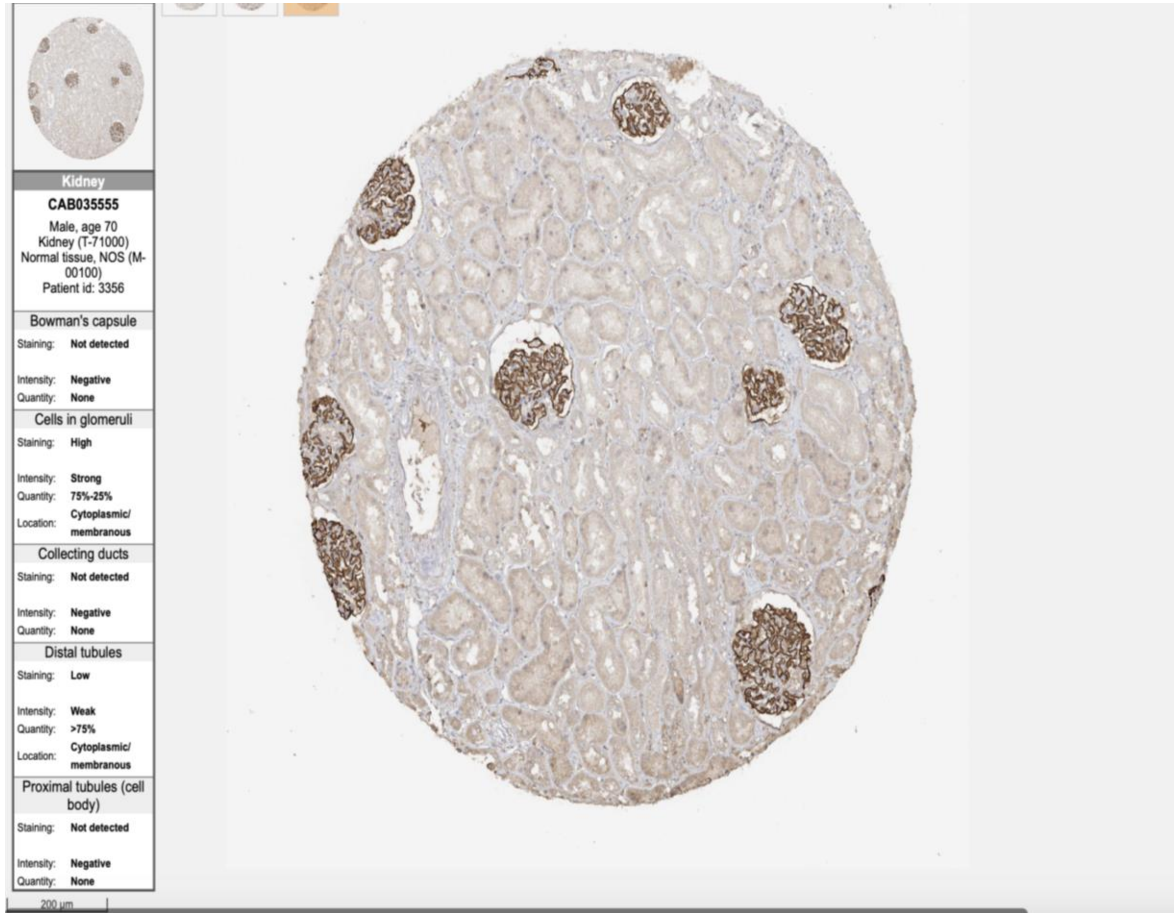
Kidney
CAB035555
Male, age 70
Kidney (T-71000)
Normal tissue, NOS (M-00100)
Patient id: 3356
Bowman's capsule
Staining: Not detected
Intensity: Negative
Quantity: None
Cells in glomeruli
Staining: High
Intensity: Strong
Quantity: 75%-25%
Location: Cytoplasmic/ membranous
Collecting ducts
Staining: Not detected
Intensity: Negative
Quantity: None
Distal tubules
Staining: Low
Intensity: Weak
Quantity: >75%
Location: Cytoplasmic/ membranous
Proximal tubules (cell body)
Staining: Not detected
Intensity: Negative
Quantity: None
200 µm

SYNPO

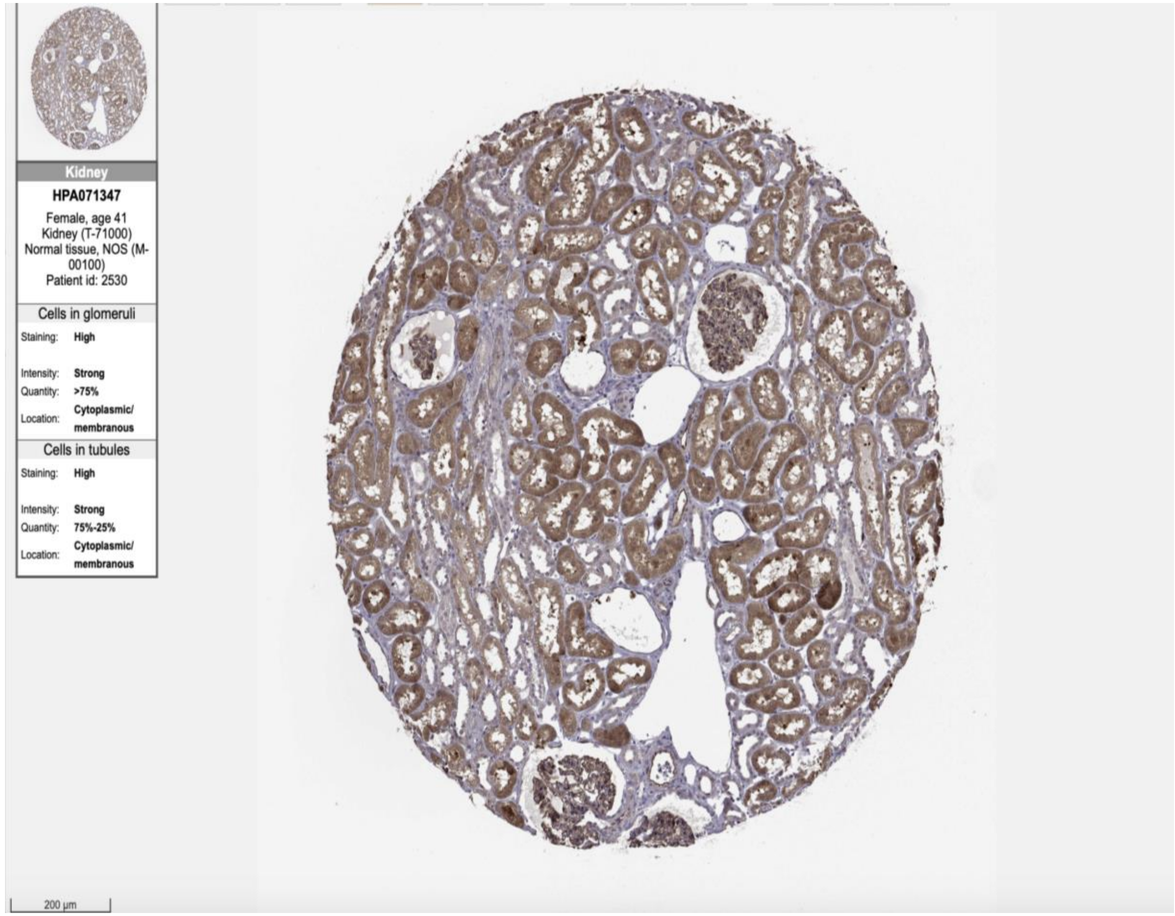
Kidney
HPA071347
Female, age 41
Kidney (T-71000)
Normal tissue, NOS (M-00100)
Patient id: 2530
Cells in glomeruli
Staining: High
Intensity: Strong
Quantity: >75%
Location: Cytoplasmic/membranous
Cells in tubules
Staining: High
Intensity: Strong
Quantity: 75%-25%
Location: Cytoplasmic/membranous
200 µm

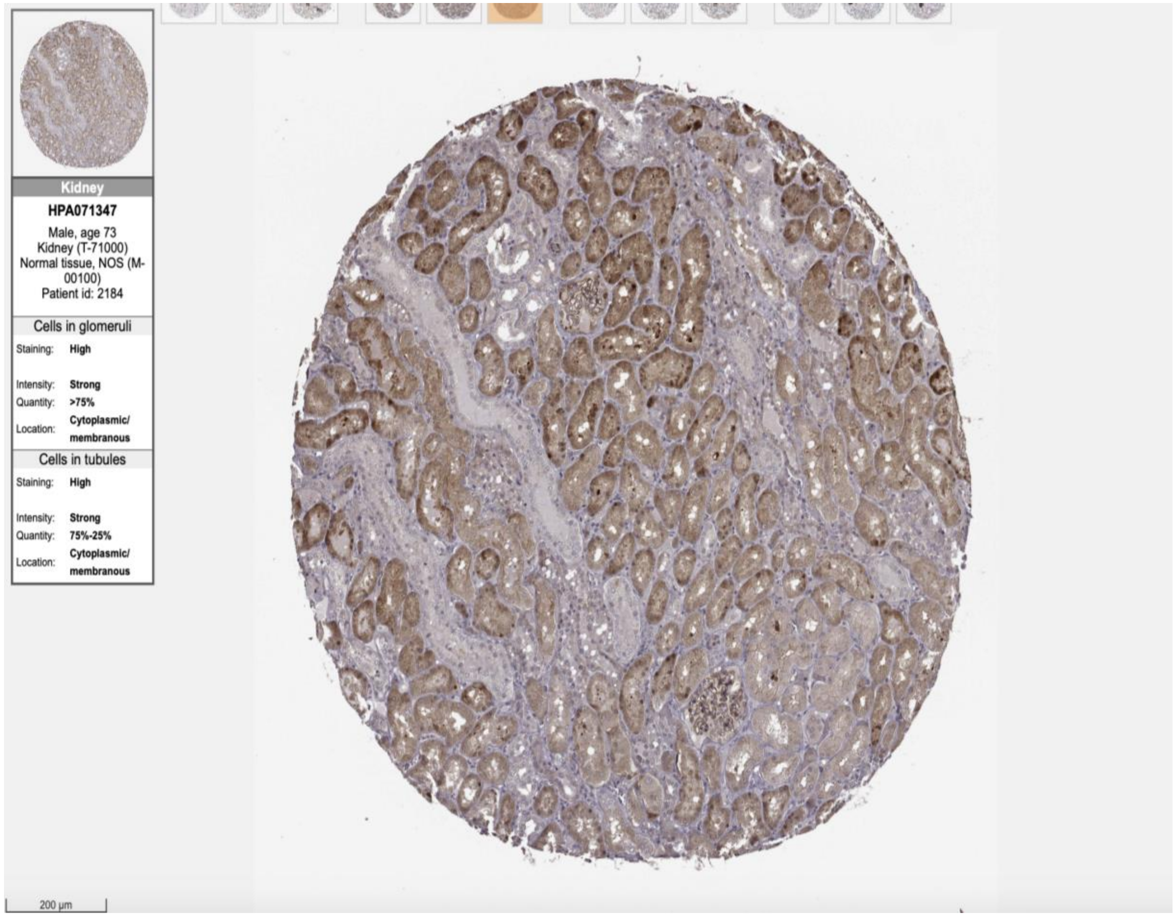
Kidney
HPA071347
Male, age 73
Kidney (T-71000)
Normal tissue, NOS (M-00100)
Patient id: 2184
Cells in glomeruli
Staining: High
Intensity: Strong
Quantity: >75%
Location: Cytoplasmic/membranous
Cells in tubules
Staining: High
Intensity: Strong
Quantity: 75%-25%
Location: Cytoplasmic/membranous
200 µm

Podocyte/Brain

transcription regulator activity (TF´s)

ATF7IP2

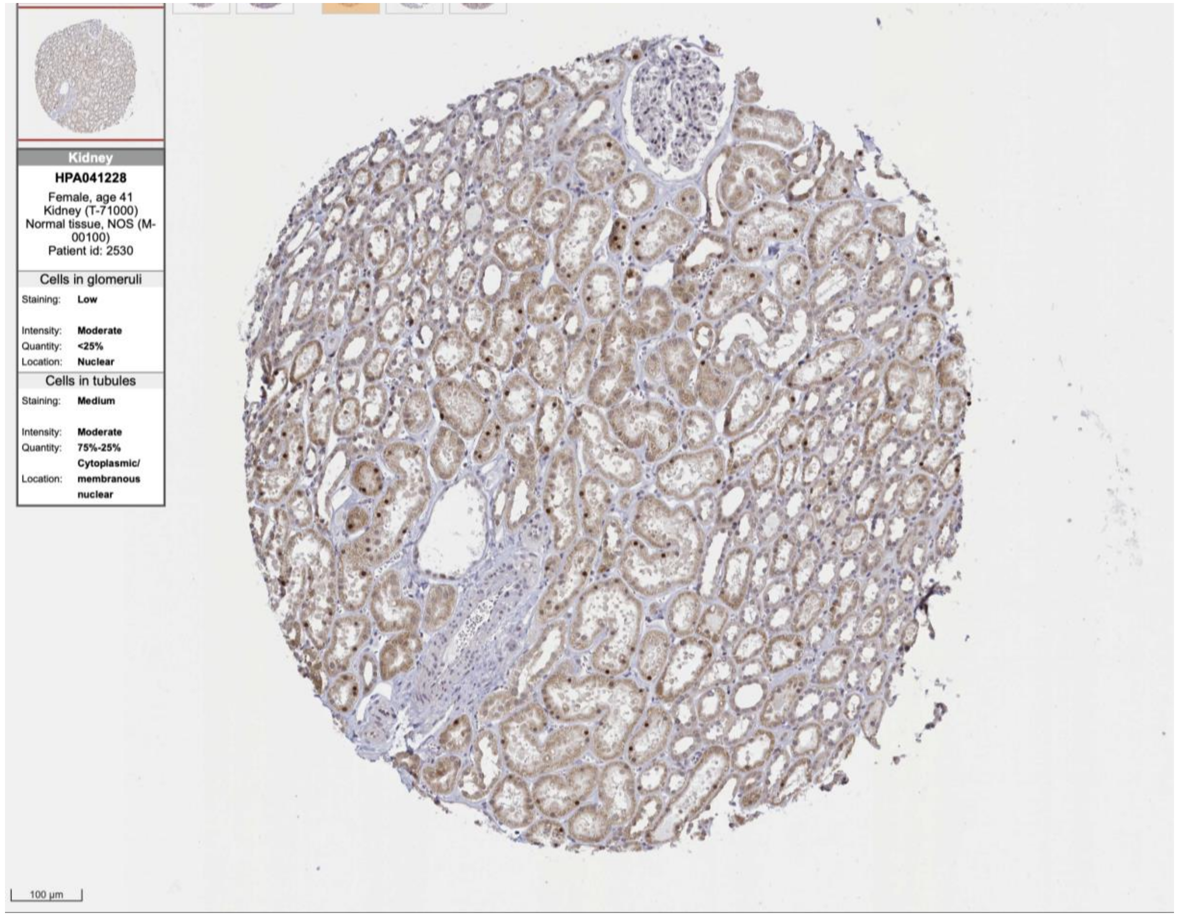

Kidney
HPA041228
Female, age 41
Kidney (T-71000)
Normal tissue, NOS (M-00100)
Patient id: 2530
Cells in glomeruli
Staining: Low
Intensity: Moderate
Quantity: <25%
Location: Nuclear
Cells in tubules
Staining: Medium
Intensity: Moderate
Quantity: 75%-25%
Location: Cytoplasmic/ membranous nuclear
100 µm

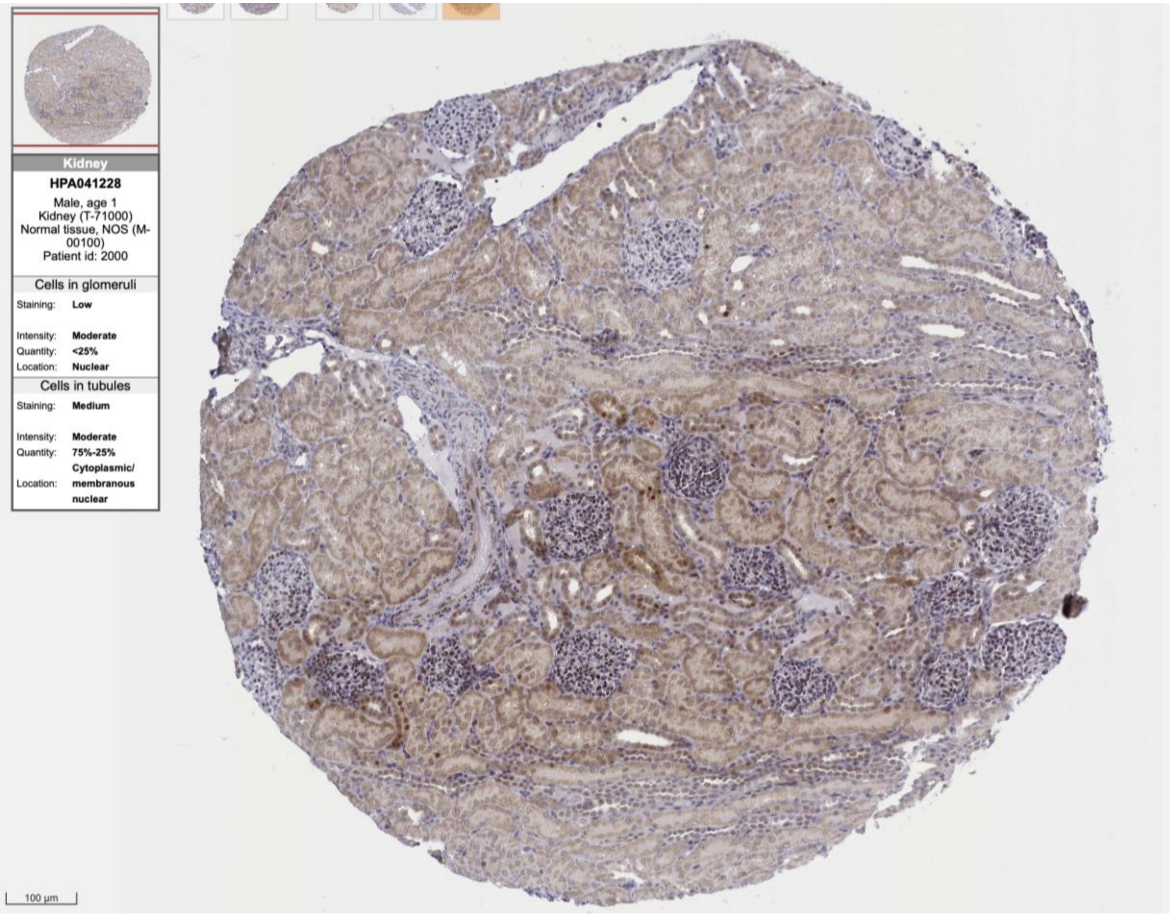

Kidney
HPA041228
Male, age 1
Kidney (T-71000)
Normal tissue, NOS (M-00100)
Patient id: 2000
Cells in glomeruli
Staining: Low
Intensity: Moderate
Quantity: <25%
Location: Nuclear
Cells in tubules
Staining: Medium
Intensity: Moderate
Quantity: 75%-25%
Location: Cytoplasmic/ membranous nuclear
100 µm

BACH2

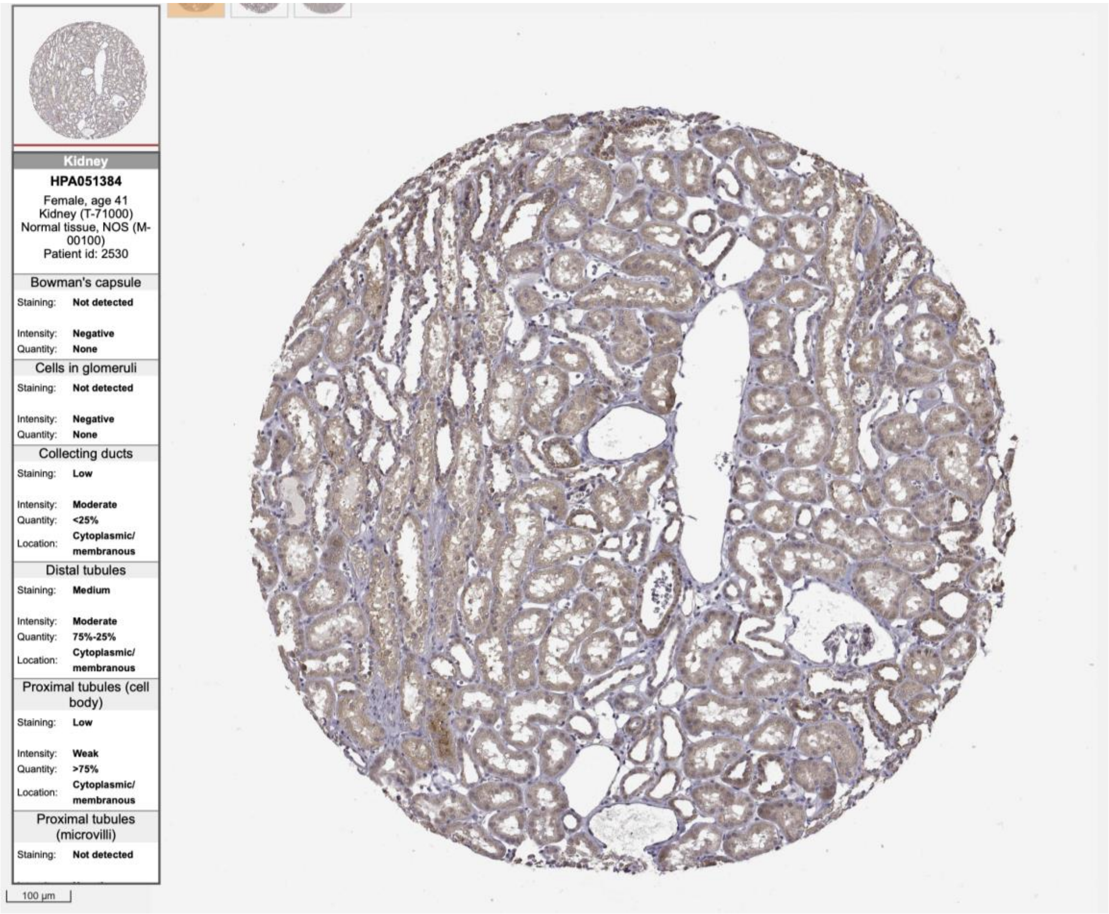
Kidney
HPA051384
Female, age 41
Kidney (T-71000)
Normal tissue, NOS (M-00100)
Patient id: 2530
Bowman's capsule
Staining: Not detected
Intensity: Negative
Quantity: None
Cells in glomeruli
Staining: Not detected
Intensity: Negative
Quantity: None
Collecting ducts
Staining: Low
Intensity: Moderate
Quantity: <25%
Location: Cytoplasmic/ membranous
Distal tubules
Staining: Medium
Intensity: Moderate
Quantity: 75%-25%
Location: Cytoplasmic/ membranous
Proximal tubules (cell body)
Staining: Low
Intensity: Weak
Quantity: >75%
Location: Cytoplasmic/ membranous
Proximal tubules (microvilli)
Staining: Not detected
100 µm

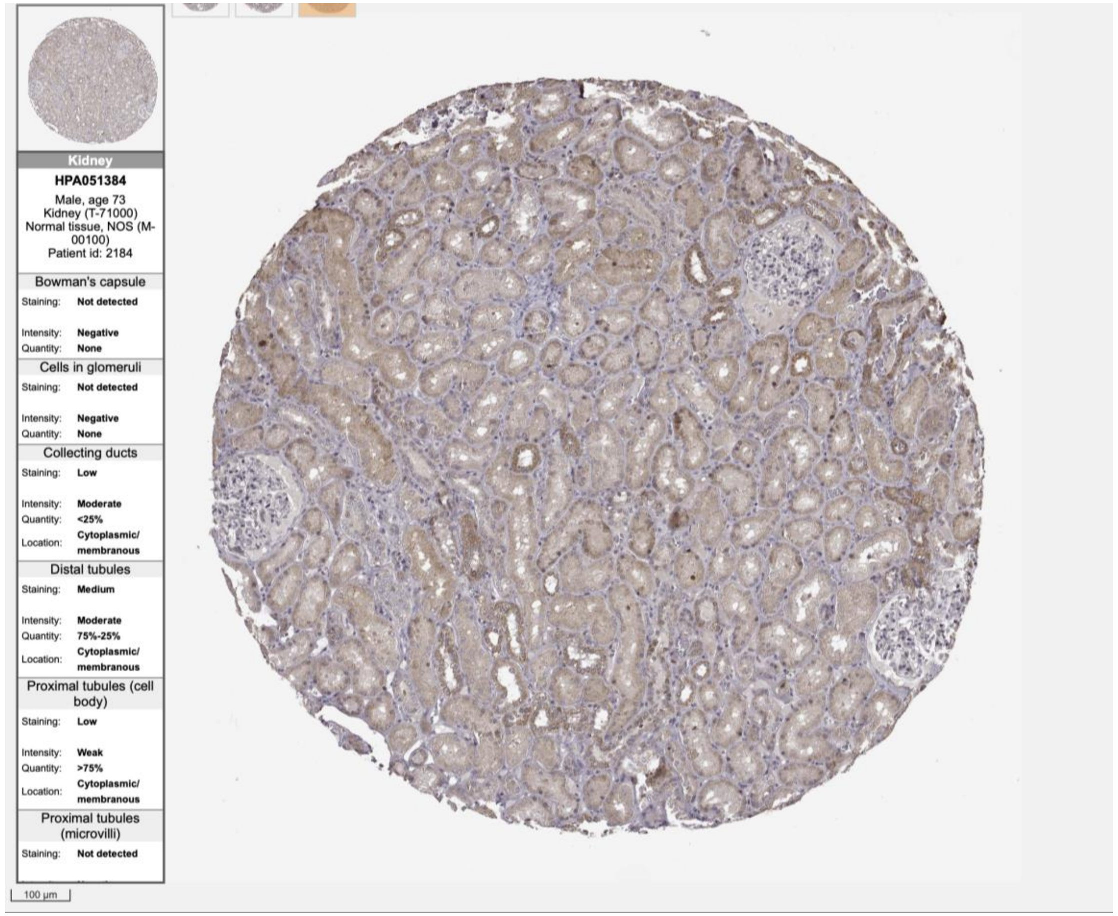
Kidney
HPA051384
Male, age 73
Kidney (T-71000)
Normal tissue, NOS (M-00100)
Patient id: 2184
Bowman's capsule
Staining: Not detected
Intensity: Negative
Quantity: None
Cells in glomeruli
Staining: Not detected
Intensity: Negative
Quantity: None
Collecting ducts
Staining: Low
Intensity: Moderate
Quantity: <25%
Location: Cytoplasmic/ membranous
Distal tubules
Staining: Medium
Intensity: Moderate
Quantity: 75%-25%
Location: Cytoplasmic/ membranous
Proximal tubules (cell body)
Staining: Low
Intensity: Weak
Quantity: >75%
Location: Cytoplasmic/ membranous
Proximal tubules (microvilli)
Staining: Not detected
100 µm

EAF2

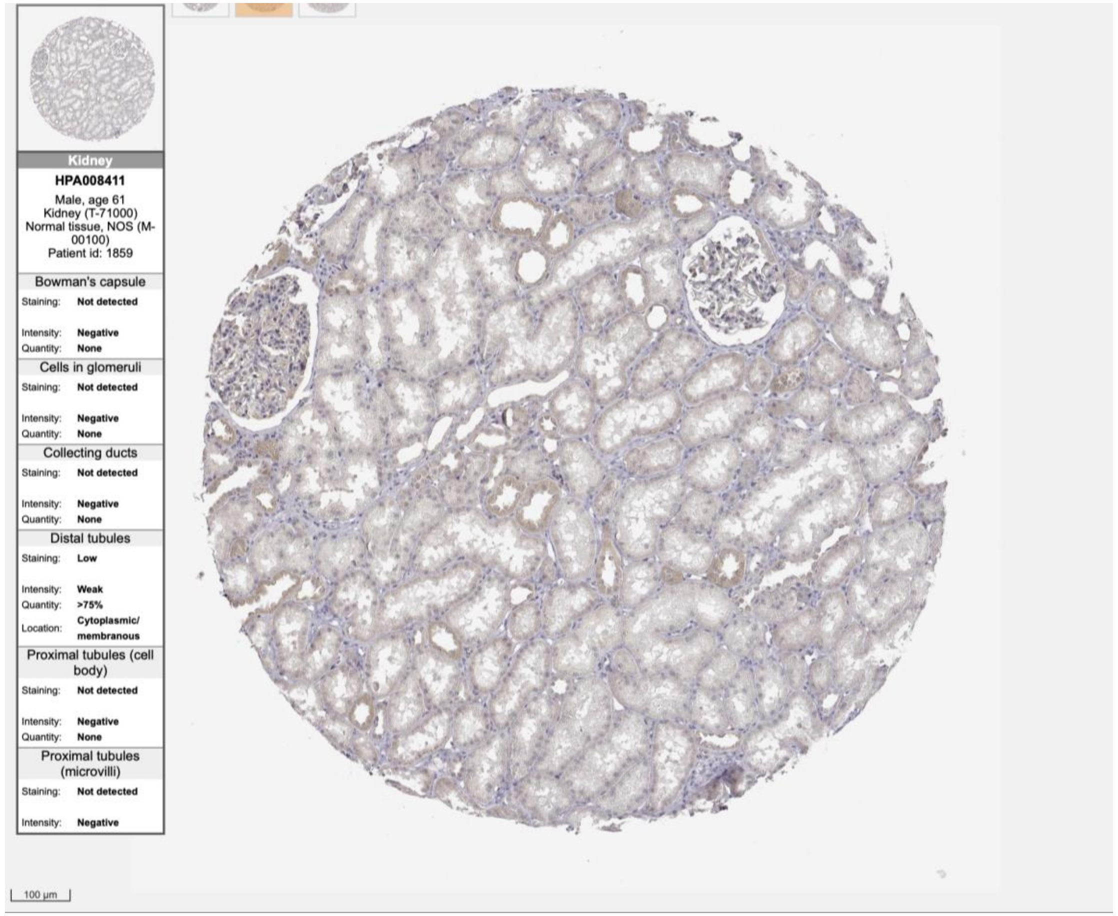
Kidney
HPA008411
Male, age 61
Kidney (T-71000)
Normal tissue, NOS (M-00100)
Patient id: 1859
Bowman's capsule
Staining: Not detected
Intensity: Negative
Quantity: None
Cells in glomeruli
Staining: Not detected
Intensity: Negative
Quantity: None
Collecting ducts
Staining: Not detected
Intensity: Negative
Quantity: None
Distal tubules
Staining: Low
Intensity: Weak
Quantity: >75%
Location: Cytoplasmic/ membranous
Proximal tubules (cell body)
Staining: Not detected
Intensity: Negative
Quantity: None
Proximal tubules (microvilli)
Staining: Not detected
Intensity: Negative
100 µm

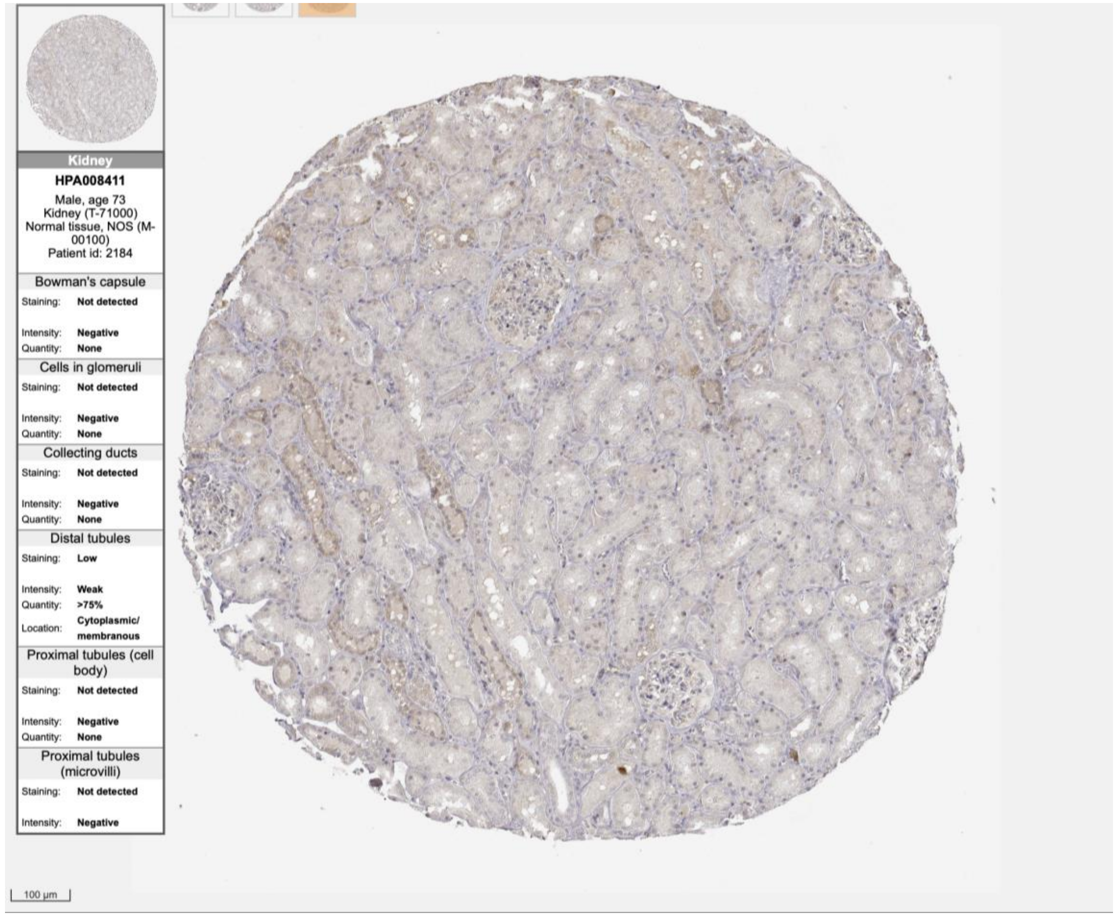
Kidney
HPA008411
Male, age 73
Kidney (T-71000)
Normal tissue, NOS (M-00100)
Patient id: 2184
Bowman's capsule
Staining: Not detected
Intensity: Negative
Quantity: None
Cells in glomeruli
Staining: Not detected
Intensity: Negative
Quantity: None
Collecting ducts
Staining: Not detected
Intensity: Negative
Quantity: None
Distal tubules
Staining: Low
Intensity: Weak
Quantity: >75%
Location: Cytoplasmic/ membranous
Proximal tubules (cell body)
Staining: Not detected
Intensity: Negative
Quantity: None
Proximal tubules (microvilli)
Staining: Not detected
Intensity: Negative
100 µm

FOXD3

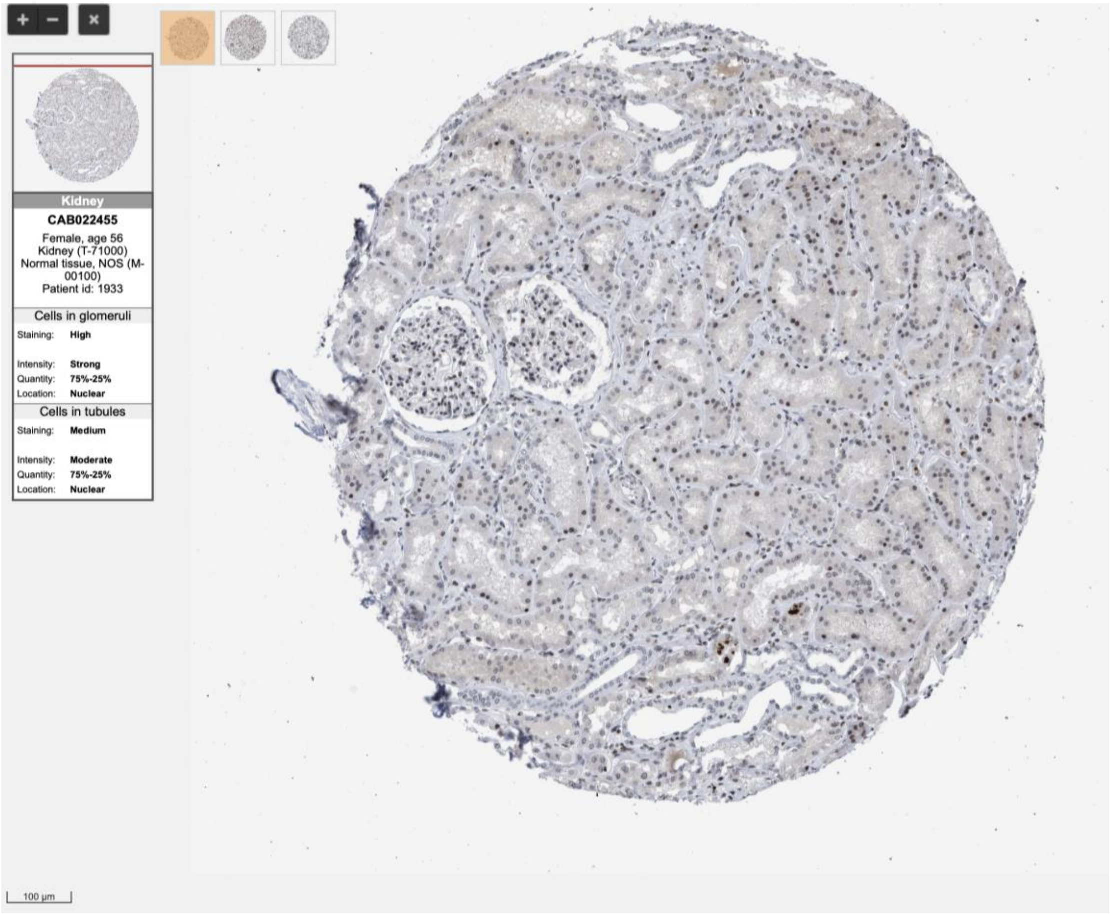

Kidney
CAB022455
Female, age 56
Kidney (T-71000)
Normal tissue, NOS (M-00100)
Patient id: 1933
Cells in glomeruli
Staining: High
Intensity: Strong
Quantity: 75%-25%
Location: Nuclear
Cells in tubules
Staining: Medium
Intensity: Moderate
Quantity: 75%-25%
Location: Nuclear
100 µm

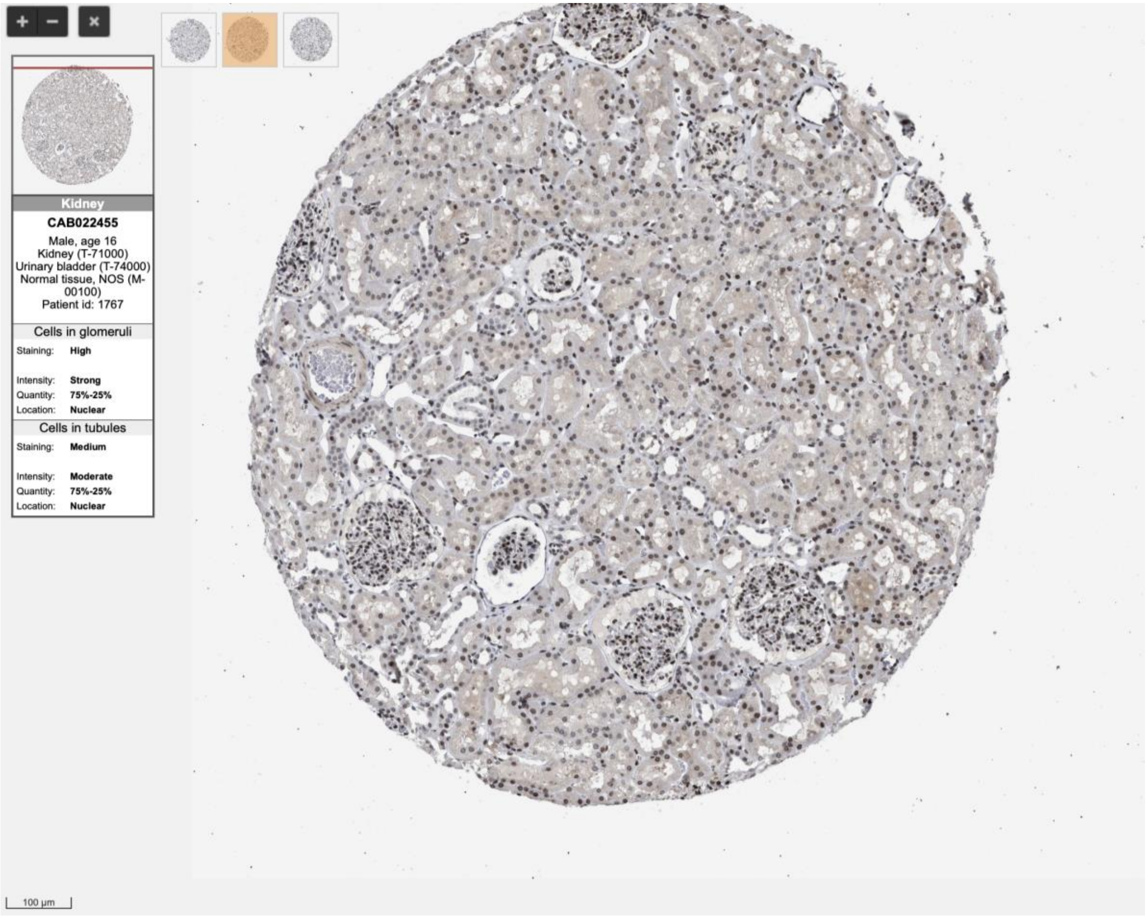

Kidney
CAB022455
Male, age 16
Kidney (T-71000)
Urinary bladder (T-74000)
Normal tissue, NOS (M-00100)
Patient id: 1767
Cells in glomeruli
Staining: High
Intensity: Strong
Quantity: 75%-25%
Location: Nuclear
Cells in tubules
Staining: Medium
Intensity: Moderate
Quantity: 75%-25%
Location: Nuclear
100 µm

FOXS1

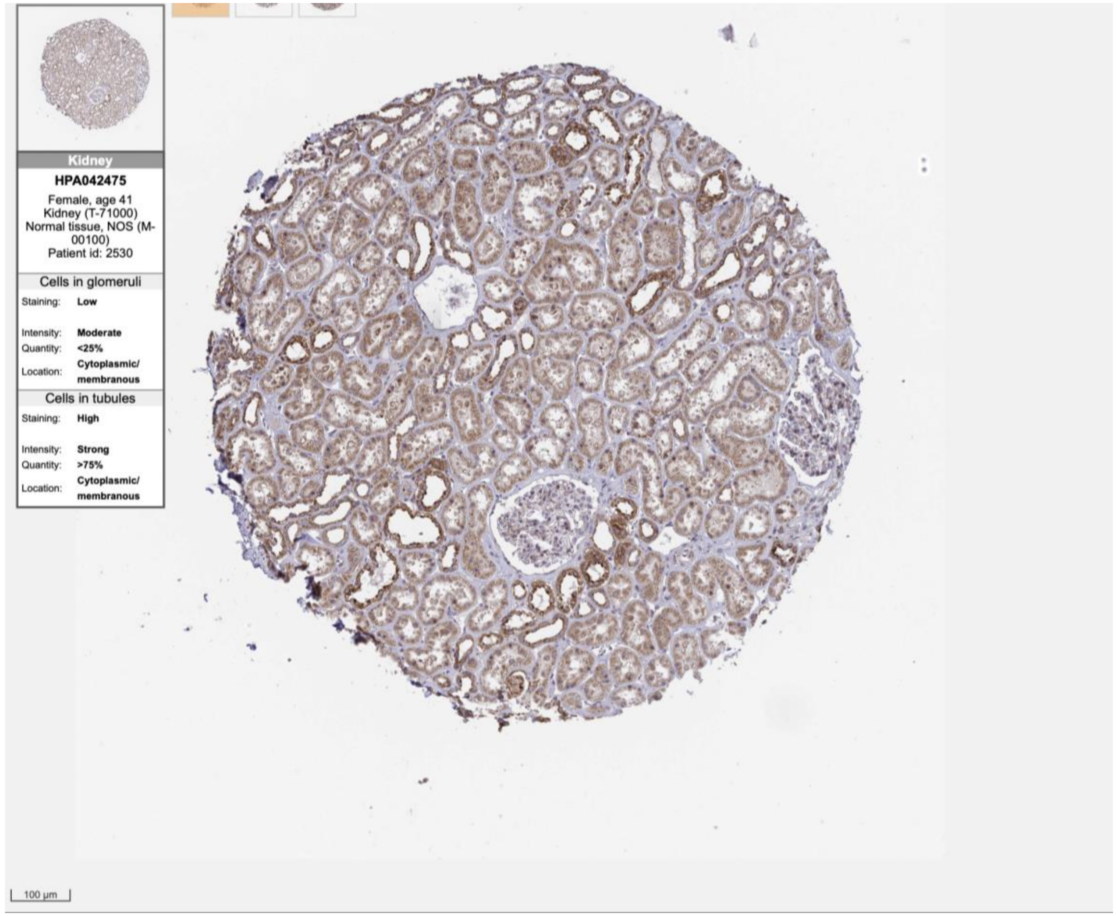
Kidney
HPA042475
Female, age 41
Kidney (T-71000)
Normal tissue, NOS (M-00100)
Patient id: 2530
Cells in glomeruli
Staining: Low
Intensity: Moderate
Quantity: <25%
Location: Cytoplasmic/ membranous
Cells in tubules
Staining: High
Intensity: Strong
Quantity: >75%
Location: Cytoplasmic/ membranous
100 µm

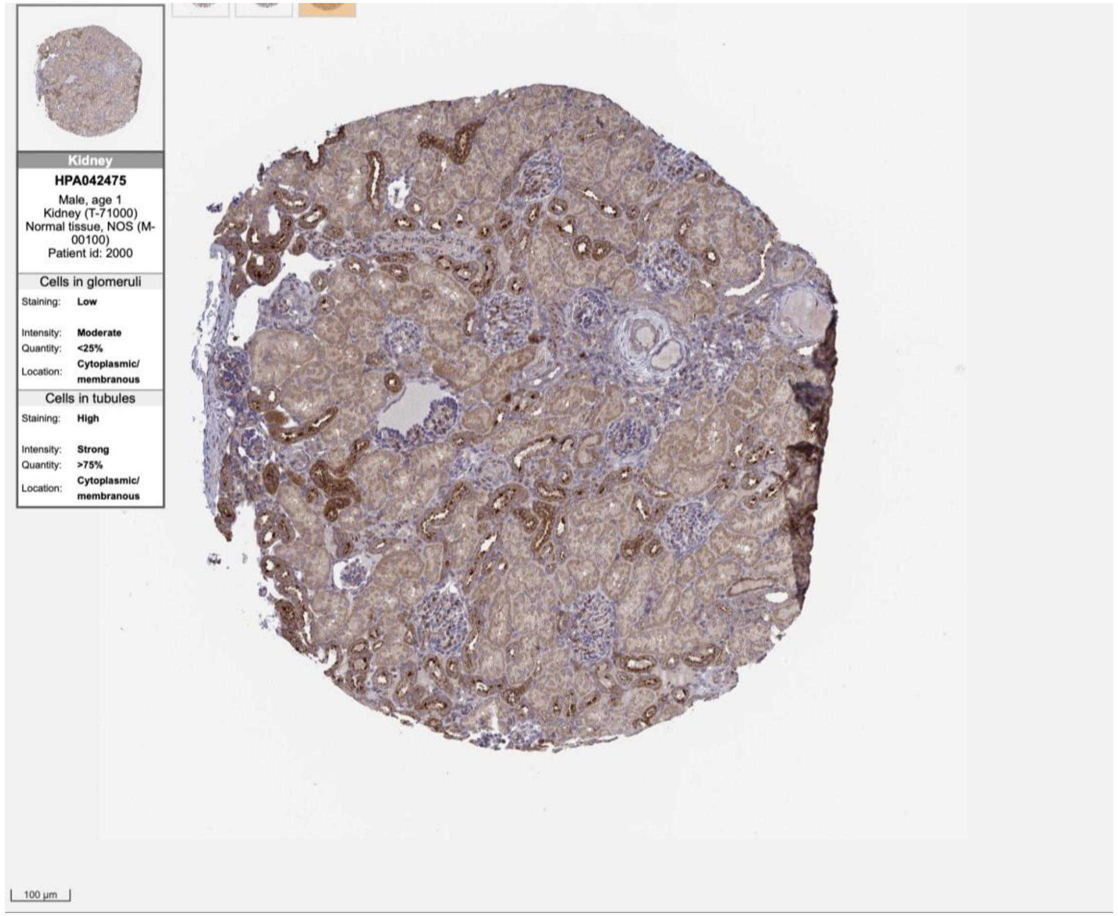
Kidney
HPA042475
Male, age 1
Kidney (T-71000)
Normal tissue, NOS (M-00100)
Patient id: 2000
Cells in glomeruli
Staining: Low
Intensity: Moderate
Quantity: <25%
Location: Cytoplasmic/ membranous
Cells in tubules
Staining: High
Intensity: Strong
Quantity: >75%
Location: Cytoplasmic/ membranous
100 µm

GATA4

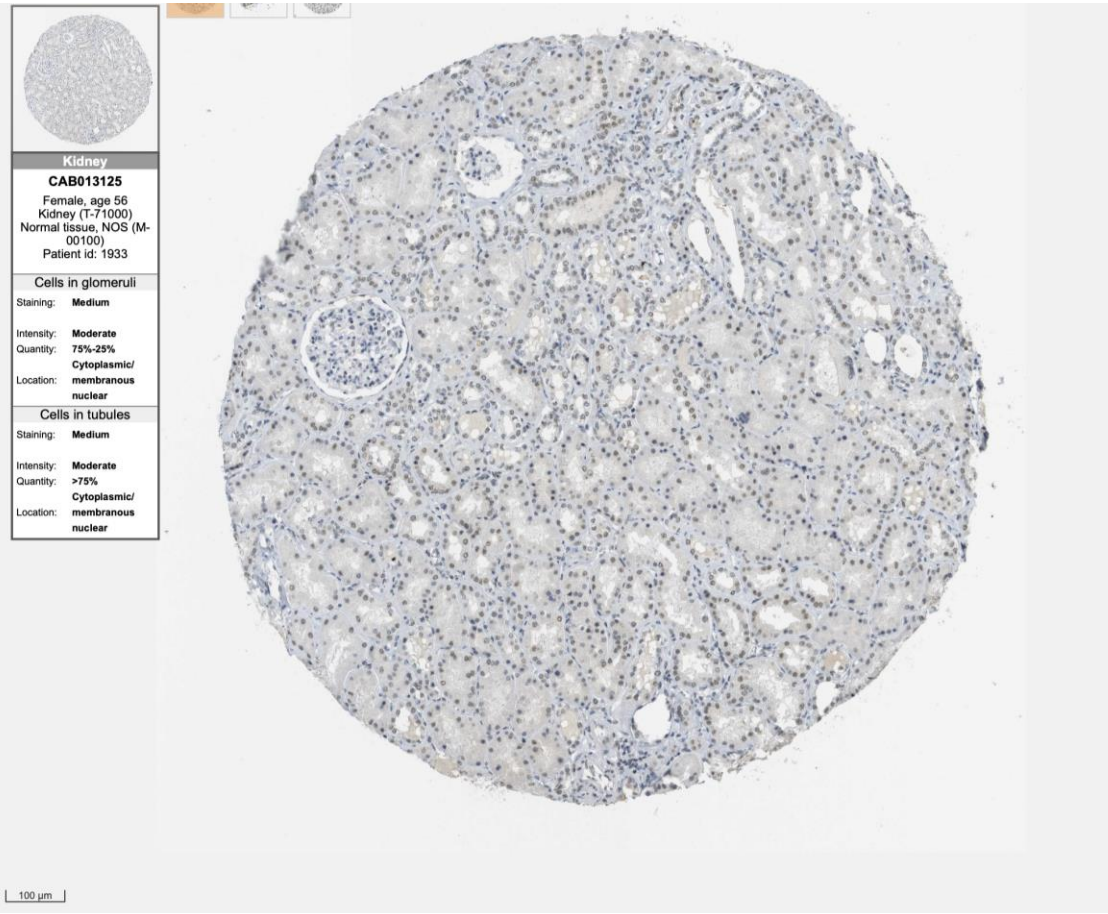
Kidney
CAB013125
Female, age 56
Kidney (T-71000)
Normal tissue, NOS (M-00100)
Patient id: 1933
Cells in glomeruli
Staining: Medium
Intensity: Moderate
Quantity: 75%-25%
Location: Cytoplasmic/ membranous nuclear
Cells in tubules
Staining: Medium
Intensity: Moderate
Quantity: >75%
Location: Cytoplasmic/ membranous nuclear
100 µm

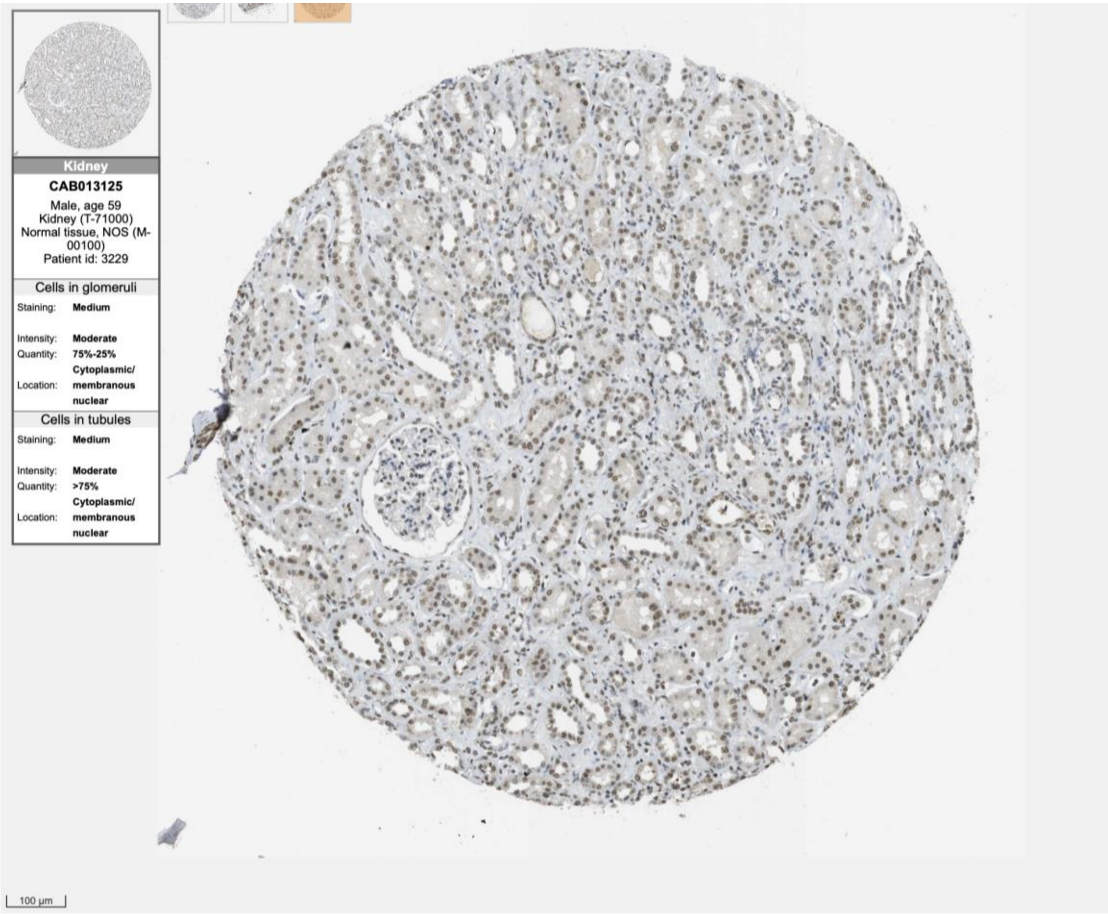
Kidney
CAB013125
Male, age 59
Kidney (T-71000)
Normal tissue, NOS (M-00100)
Patient id: 3229
Cells in glomeruli
Staining: Medium
Intensity: Moderate
Quantity: 75%-25%
Location: Cytoplasmic/ membranous nuclear
Cells in tubules
Staining: Medium
Intensity: Moderate
Quantity: >75%
Location: Cytoplasmic/ membranous nuclear
100 µm

MEIS1

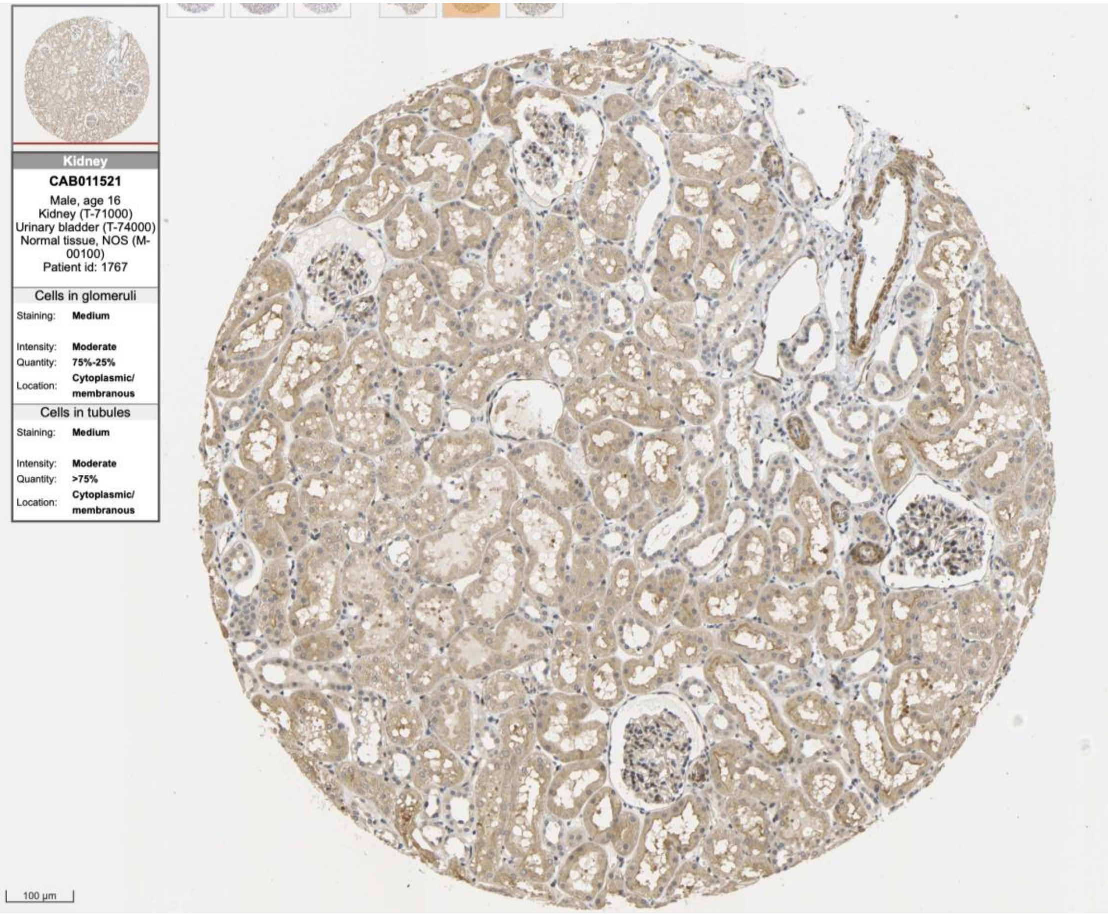
Kidney
CAB011521
Male, age 16
Kidney (T-71000)
Urinary bladder (T-74000)
Normal tissue, NOS (M-00100)
Patient id: 1767
Cells in glomeruli
Staining: Medium
Intensity: Moderate
Quantity: 75%-25%
Location: Cytoplasmic/ membranous
Cells in tubules
Staining: Medium
Intensity: Moderate
Quantity: >75%
Location: Cytoplasmic/ membranous
100 µm

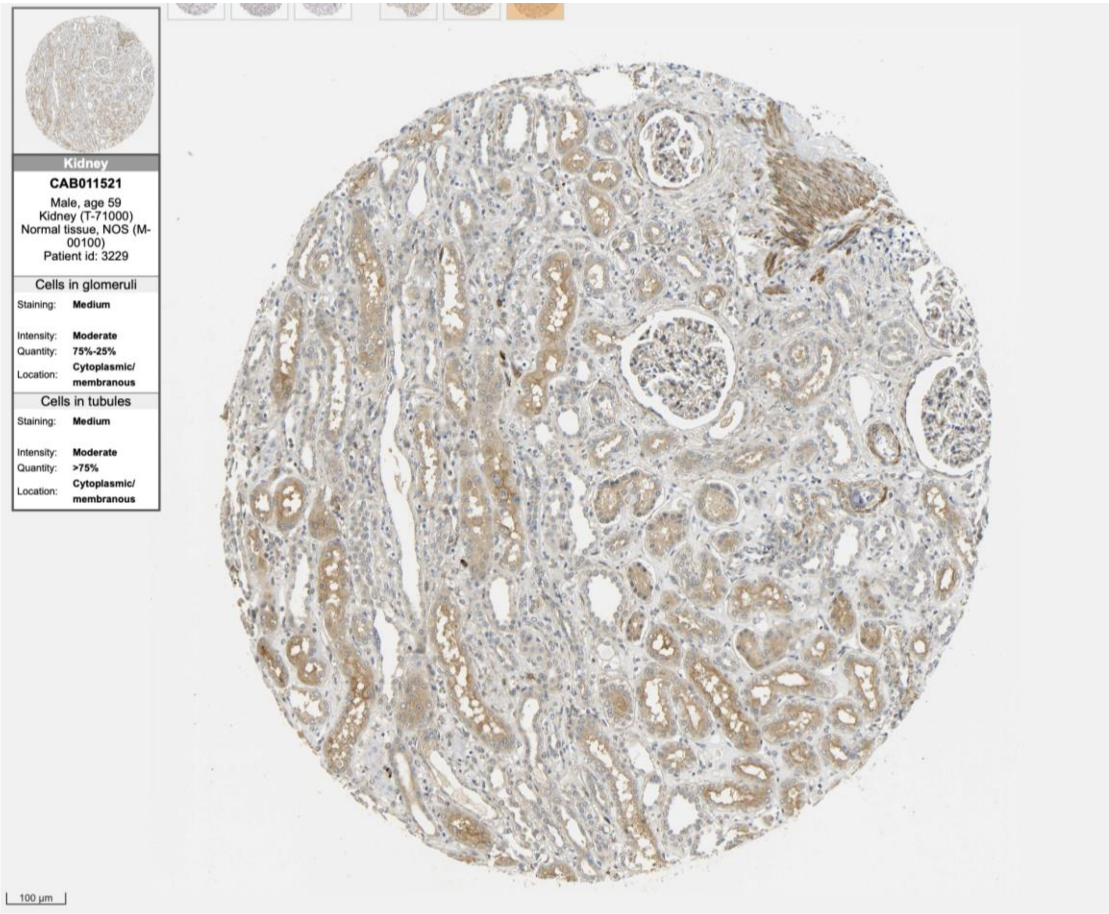
Kidney
CAB011521
Male, age 59
Kidney (T-71000)
Normal tissue, NOS (M-00100)
Patient id: 3229
Cells in glomeruli
Staining: Medium
Intensity: Moderate
Quantity: 75%-25%
Location: Cytoplasmic/ membranous
Cells in tubules
Staining: Medium
Intensity: Moderate
Quantity: >75%
Location: Cytoplasmic/ membranous
100 µm

SFMBT2

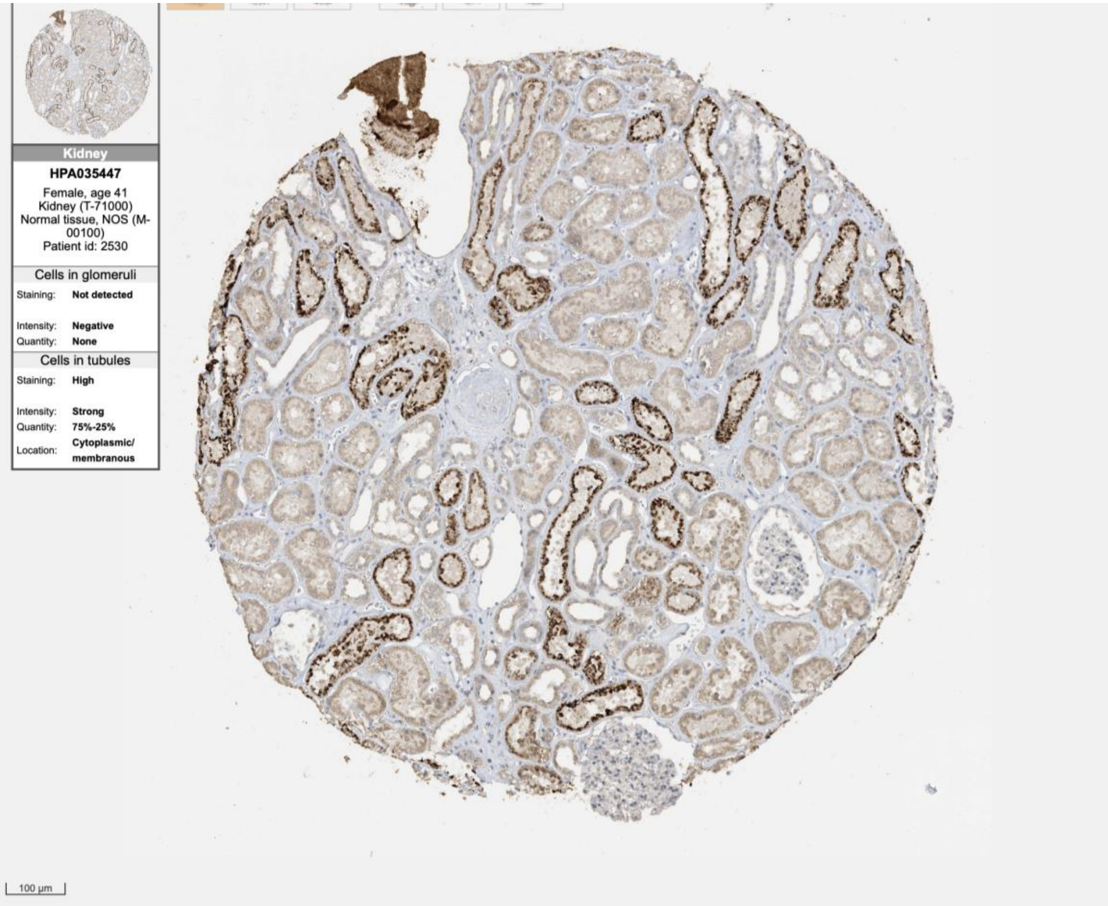

Kidney
HPA035447
Female, age 41
Kidney (T-71000)
Normal tissue, NOS (M-00100)
Patient id: 2530
Cells in glomeruli
Staining: Not detected
Intensity: Negative
Quantity: None
Cells in tubules
Staining: High
Intensity: Strong
Quantity: 75%-25%
Location: Cytoplasmic/ membranous
100 µm

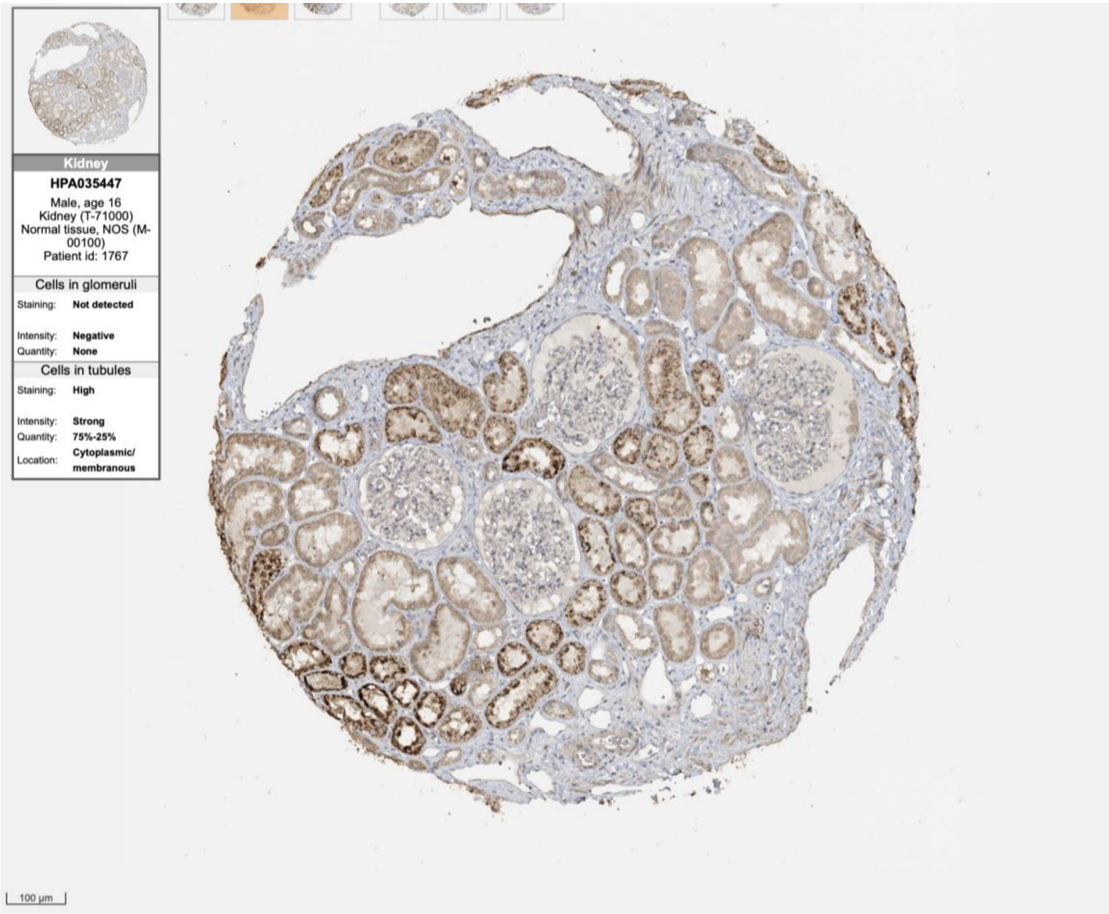

Kidney
HPA035447
Male, age 16
Kidney (T-71000)
Normal tissue, NOS (M-00100)
Patient id: 1767
Cells in glomeruli
Staining: Not detected
Intensity: Negative
Quantity: None
Cells in tubules
Staining: High
Intensity: Strong
Quantity: 75%-25%
Location: Cytoplasmic/ membranous
100 µm

PSMC3IP

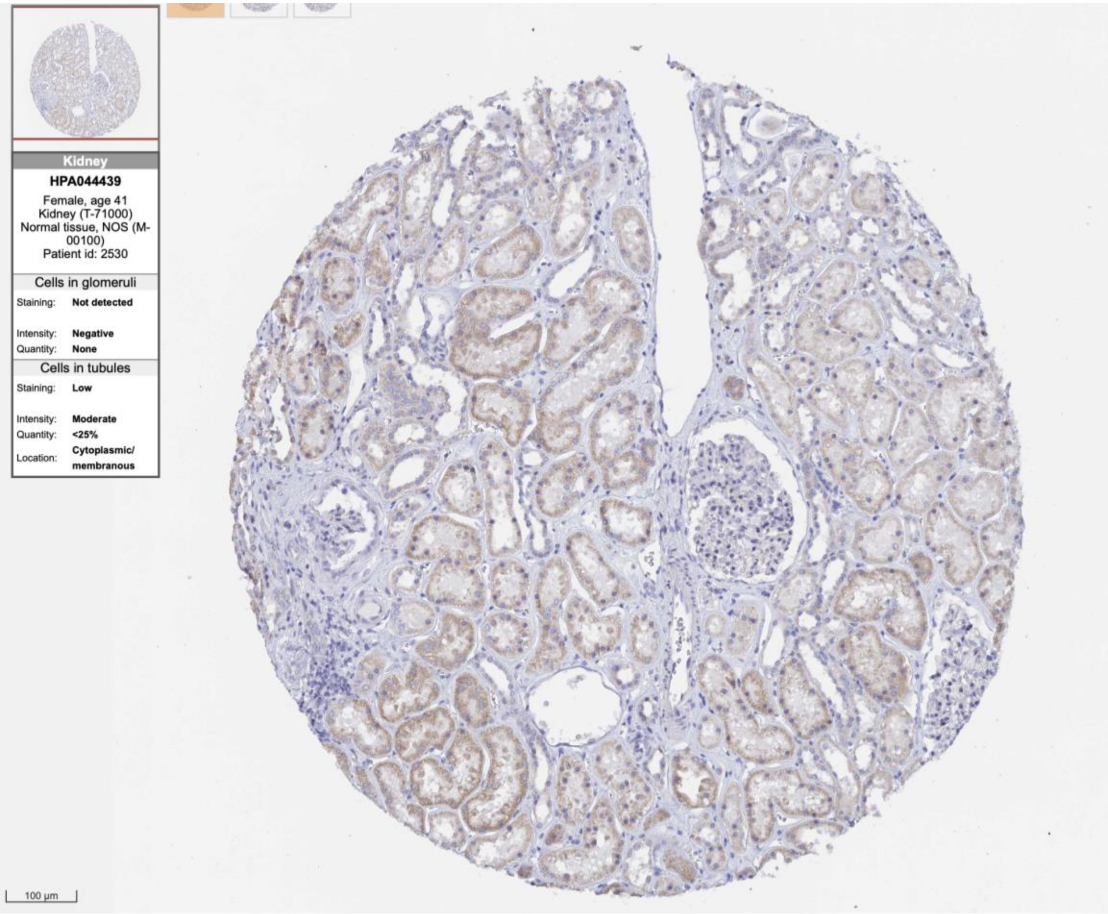
Kidney
HPA044439
Female, age 41
Kidney (T-71000)
Normal tissue, NOS (M-00100)
Patient id: 2530
Cells in glomeruli
Staining: Not detected
Intensity: Negative
Quantity: None
Cells in tubules
Staining: Low
Intensity: Moderate
Quantity: <25%
Location: Cytoplasmic/ membranous
100 µm

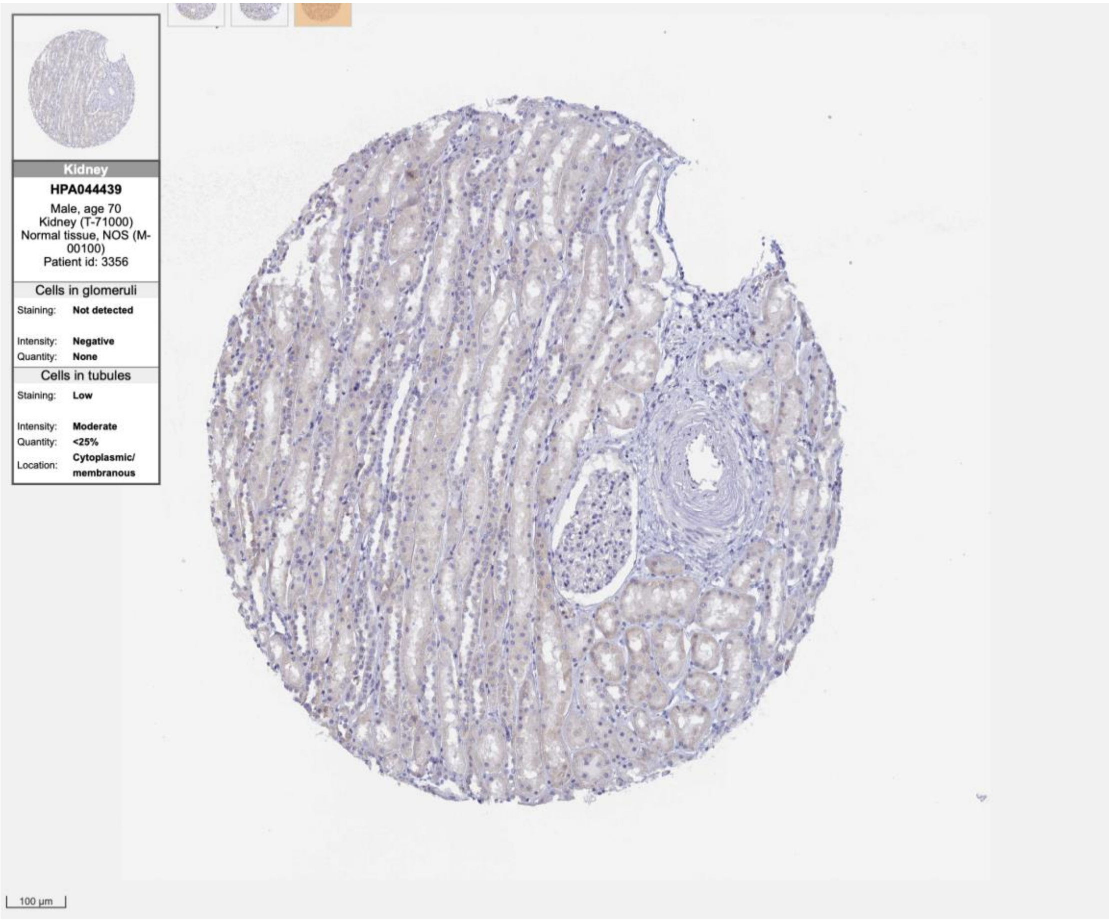
Kidney
HPA044439
Male, age 70
Kidney (T-71000)
Normal tissue, NOS (M-00100)
Patient id: 3356
Cells in glomeruli
Staining: Not detected
Intensity: Negative
Quantity: None
Cells in tubules
Staining: Low
Intensity: Moderate
Quantity: <25%
Location: Cytoplasmic/ membranous
100 µm

TFAP2B

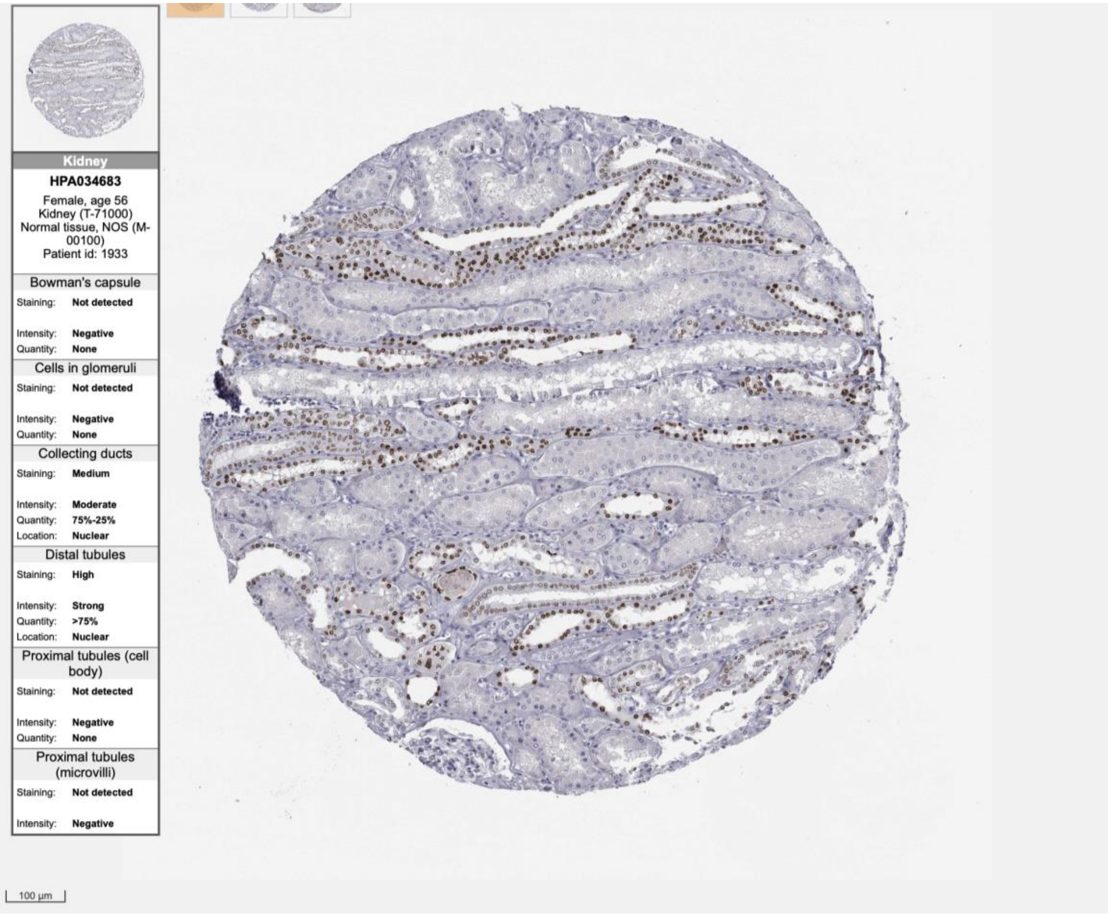
Kidney
HPA034683
Female, age 56
Kidney (T-71000)
Normal tissue, NOS (M-00100)
Patient id: 1933
Bowman's capsule
Staining: Not detected
Intensity: Negative
Quantity: None
Cells in glomeruli
Staining: Not detected
Intensity: Negative
Quantity: None
Collecting ducts
Staining: Medium
Intensity: Moderate
Quantity: 75%-25%
Location: Nuclear
Distal tubules
Staining: High
Intensity: Strong
Quantity: >75%
Location: Nuclear
Proximal tubules (cell body)
Staining: Not detected
Intensity: Negative
Quantity: None
Proximal tubules (microvilli)
Staining: Not detected
Intensity: Negative
100 µm

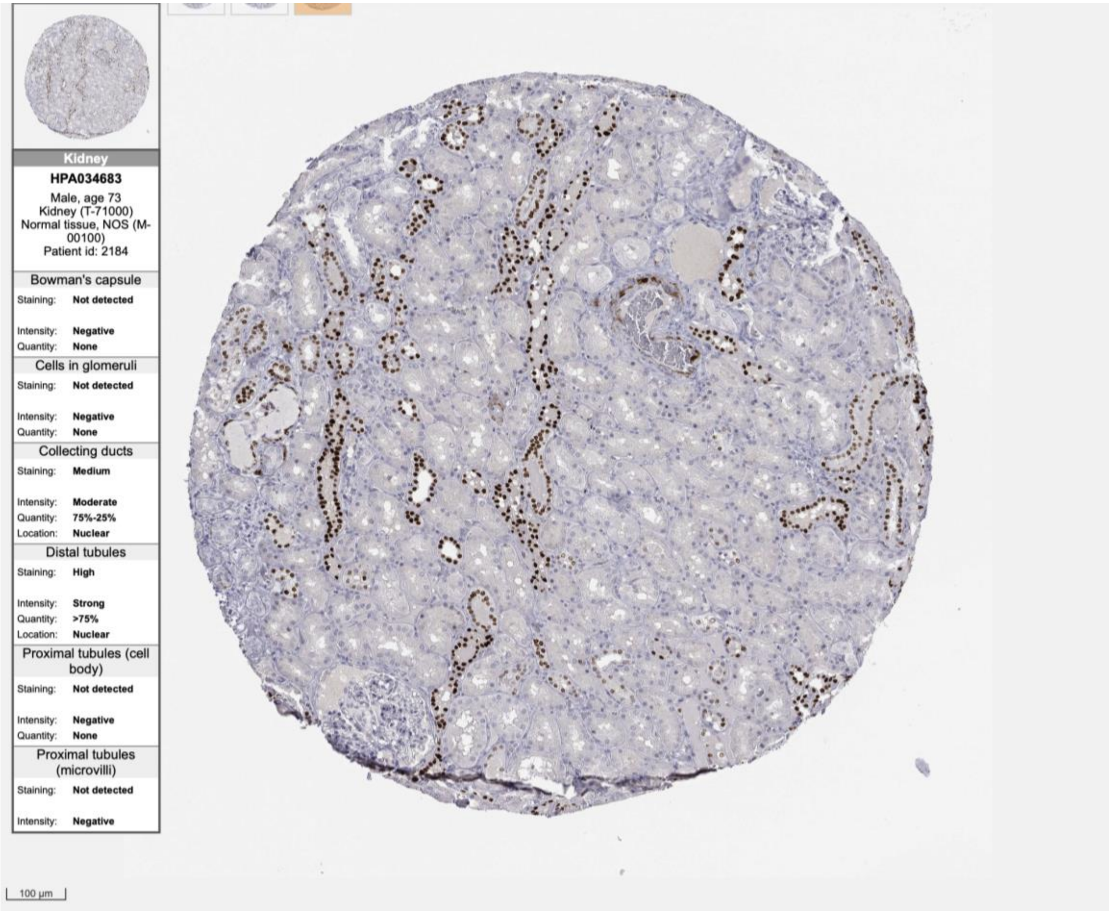
Kidney
HPA034683
Male, age 73
Kidney (T-71000)
Normal tissue, NOS (M-00100)
Patient id: 2184
Bowman's capsule
Staining: Not detected
Intensity: Negative
Quantity: None
Cells in glomeruli
Staining: Not detected
Intensity: Negative
Quantity: None
Collecting ducts
Staining: Medium
Intensity: Moderate
Quantity: 75%-25%
Location: Nuclear
Distal tubules
Staining: High
Intensity: Strong
Quantity: >75%
Location: Nuclear
Proximal tubules (cell body)
Staining: Not detected
Intensity: Negative
Quantity: None
Proximal tubules (microvilli)
Staining: Not detected
Intensity: Negative
100 µm

ZNF343

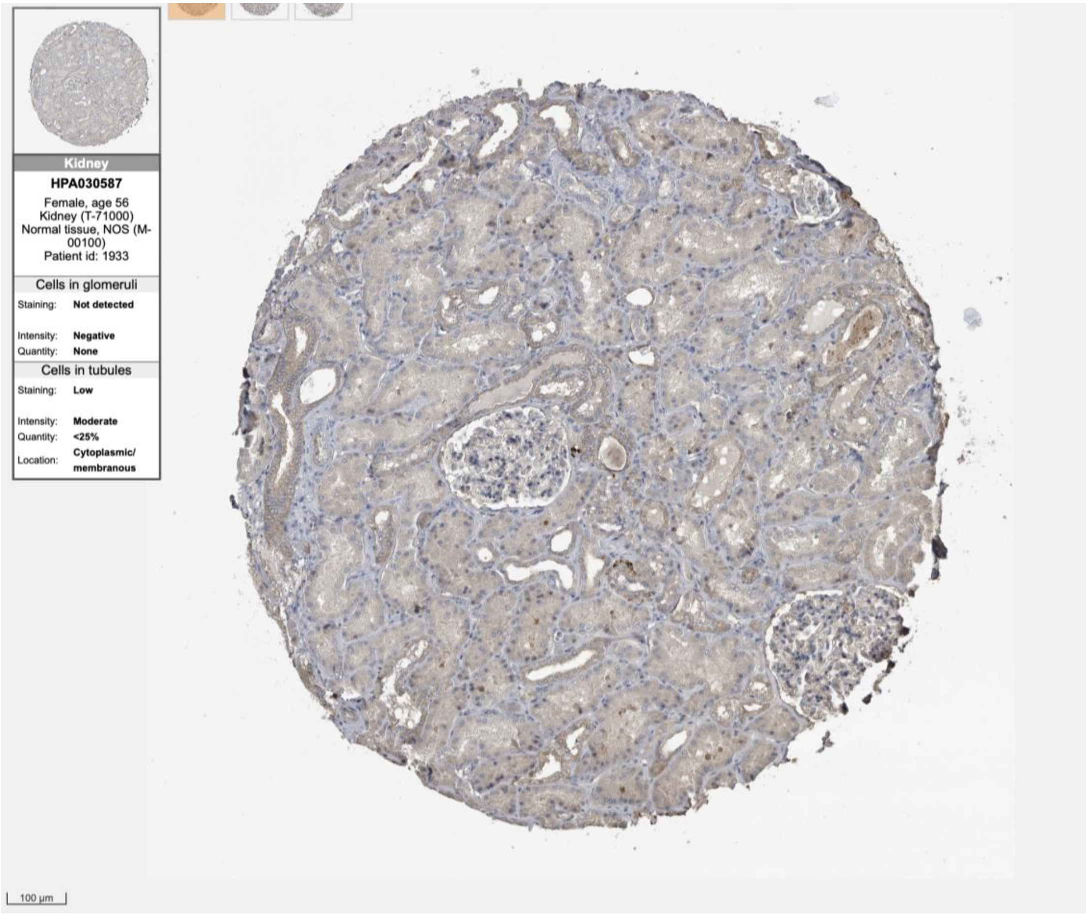
Kidney
HPA030587
Female, age 56
Kidney (T-71000)
Normal tissue, NOS (M-00100)
Patient id: 1933
Cells in glomeruli
Staining: Not detected
Intensity: Negative
Quantity: None
Cells in tubules
Staining: Low
Intensity: Moderate
Quantity: <25%
Location: Cytoplasmic/ membranous
100 µm

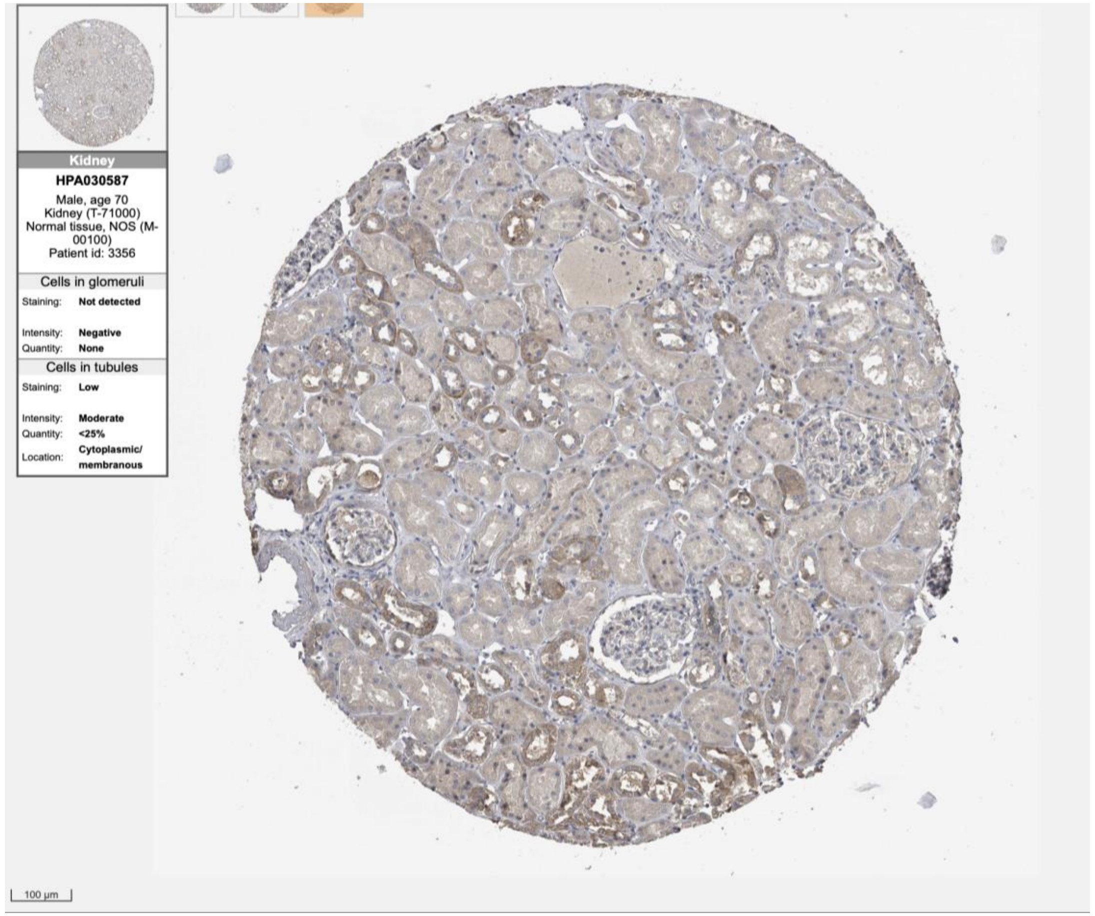
Kidney
HPA030587
Male, age 70
Kidney (T-71000)
Normal tissue, NOS (M-00100)
Patient id: 3356
Cells in glomeruli
Staining: Not detected
Intensity: Negative
Quantity: None
Cells in tubules
Staining: Low
Intensity: Moderate
Quantity: <25%
Location: Cytoplasmic/ membranous
100 µm

ZNF367

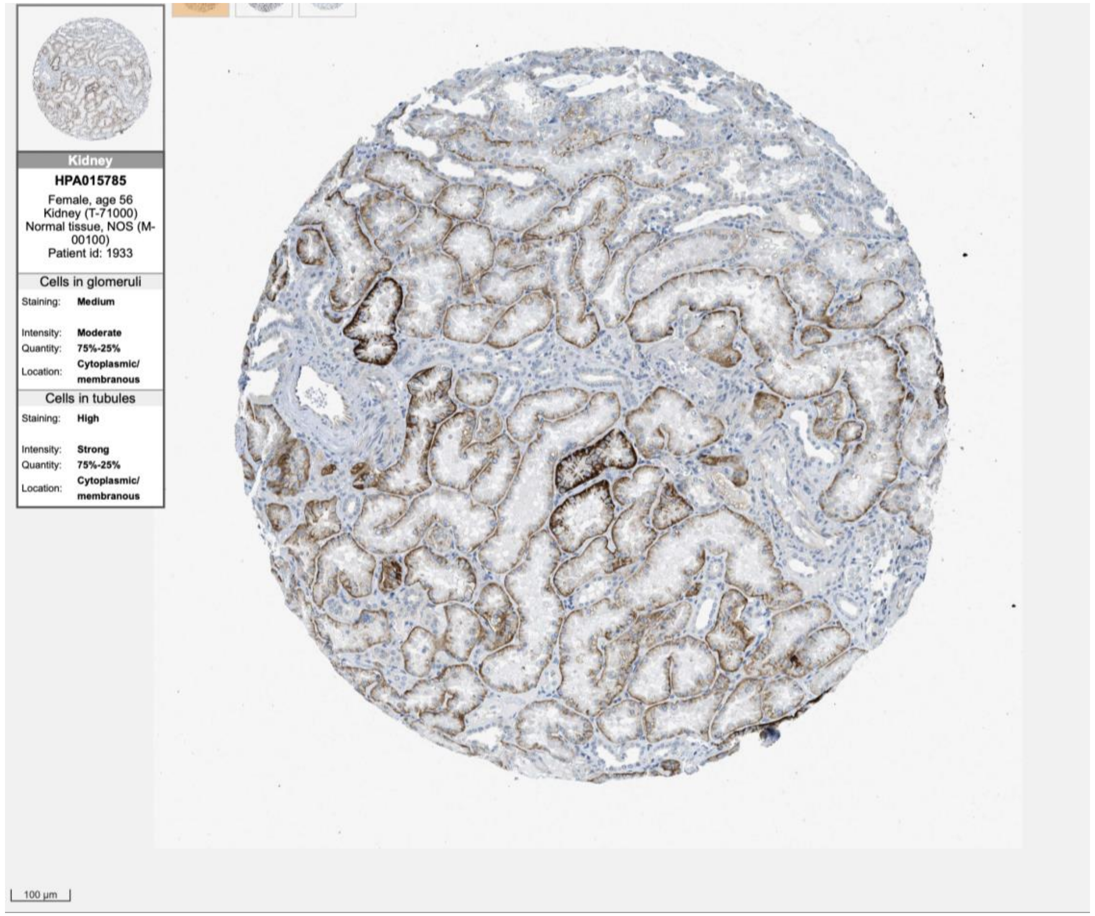
Kidney
HPA015785
Female, age 56
Kidney (T-71000)
Normal tissue, NOS (M-00100)
Patient id: 1933
Cells in glomeruli
Staining: Medium
Intensity: Moderate
Quantity: 75%-25%
Location: Cytoplasmic/membranous
Cells in tubules
Staining: High
Intensity: Strong
Quantity: 75%-25%
Location: Cytoplasmic/membranous
100 µm

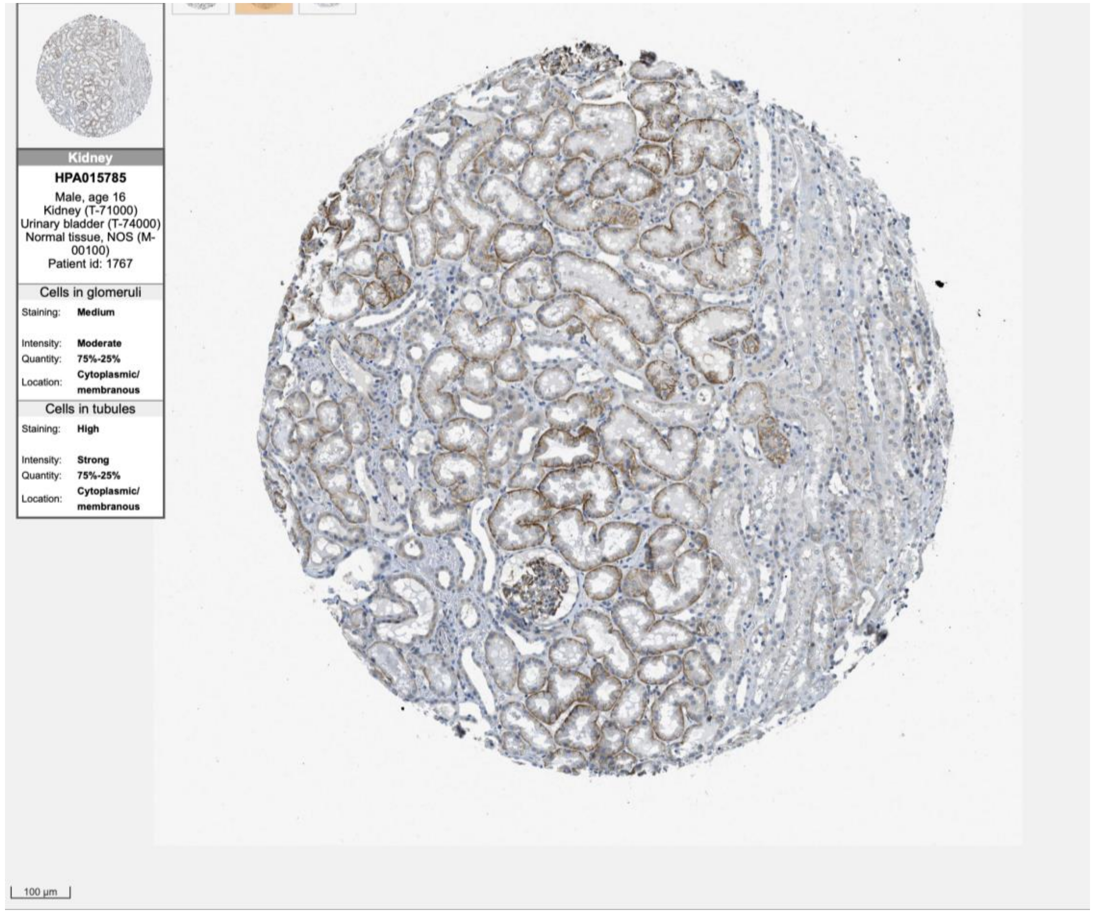
Kidney
HPA015785
Male, age 16
Kidney (T-71000)
Urinary bladder (T-74000)
Normal tissue, NOS (M-00100)
Patient id: 1767
Cells in glomeruli
Staining: Medium
Intensity: Moderate
Quantity: 75%-25%
Location: Cytoplasmic/membranous
Cells in tubules
Staining: High
Intensity: Strong
Quantity: 75%-25%
Location: Cytoplasmic/membranous
100 µm

ZNF529

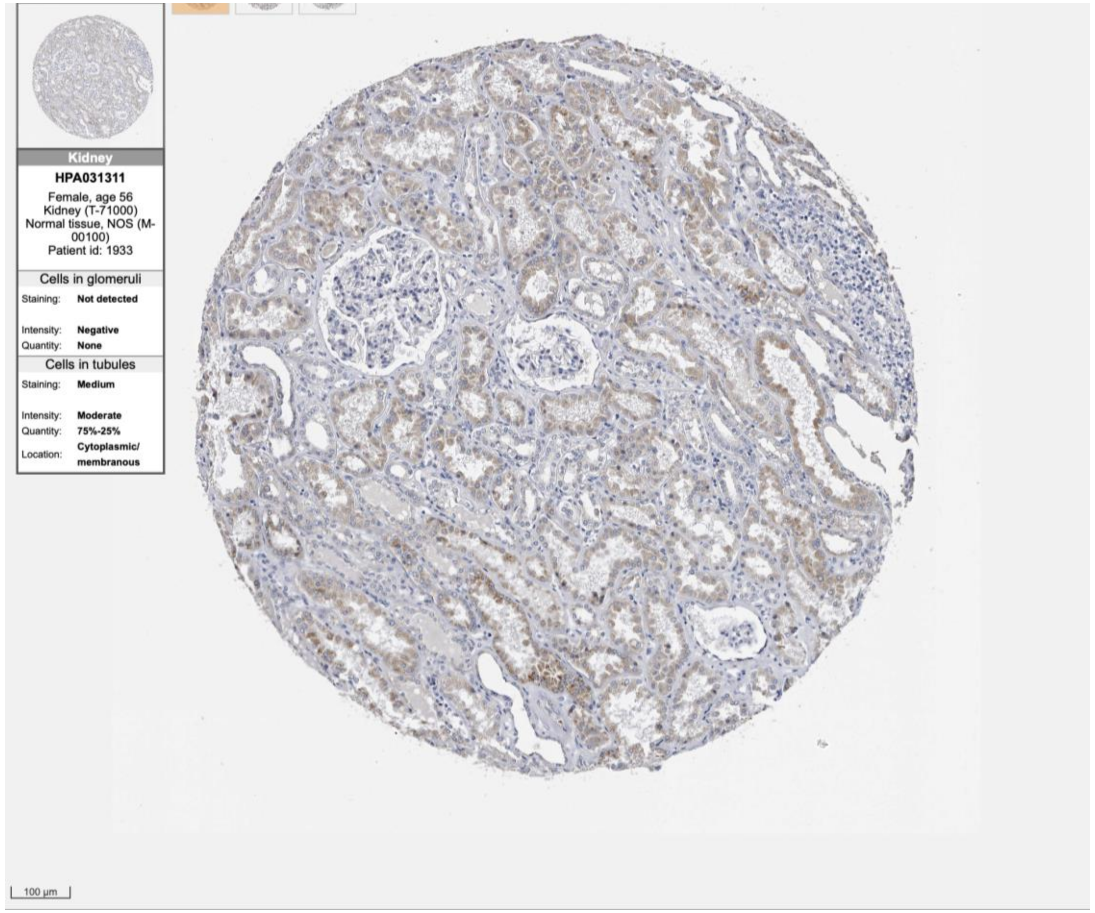
Kidney
HPA031311
Female, age 56
Kidney (T-71000)
Normal tissue, NOS (M-00100)
Patient id: 1933
Cells in glomeruli
Staining: Not detected
Intensity: Negative
Quantity: None
Cells in tubules
Staining: Medium
Intensity: Moderate
Quantity: 75%-25%
Location: Cytoplasmic/ membranous
100 µm

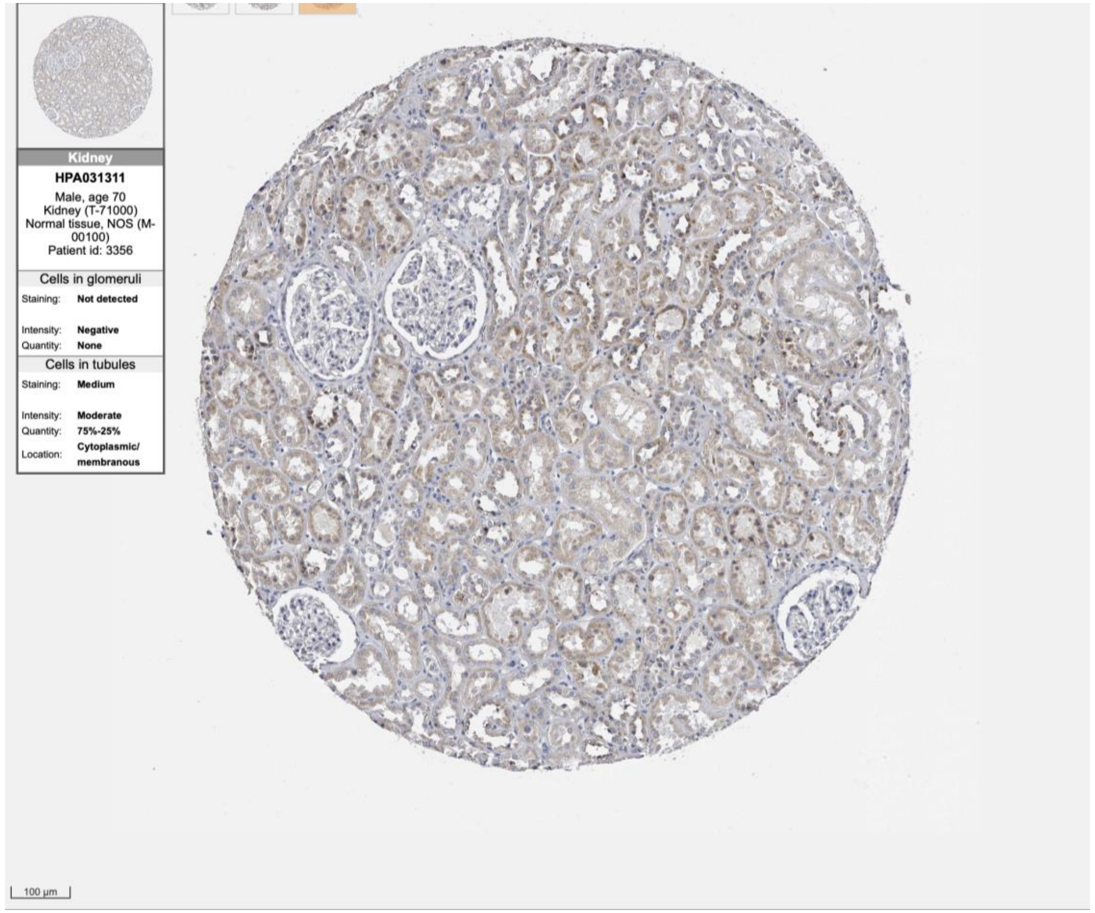
Kidney
HPA031311
Male, age 70
Kidney (T-71000)
Normal tissue, NOS (M-00100)
Patient id: 3356
Cells in glomeruli
Staining: Not detected
Intensity: Negative
Quantity: None
Cells in tubules
Staining: Medium
Intensity: Moderate
Quantity: 75%-25%
Location: Cytoplasmic/ membranous
100 µm

ZNF665

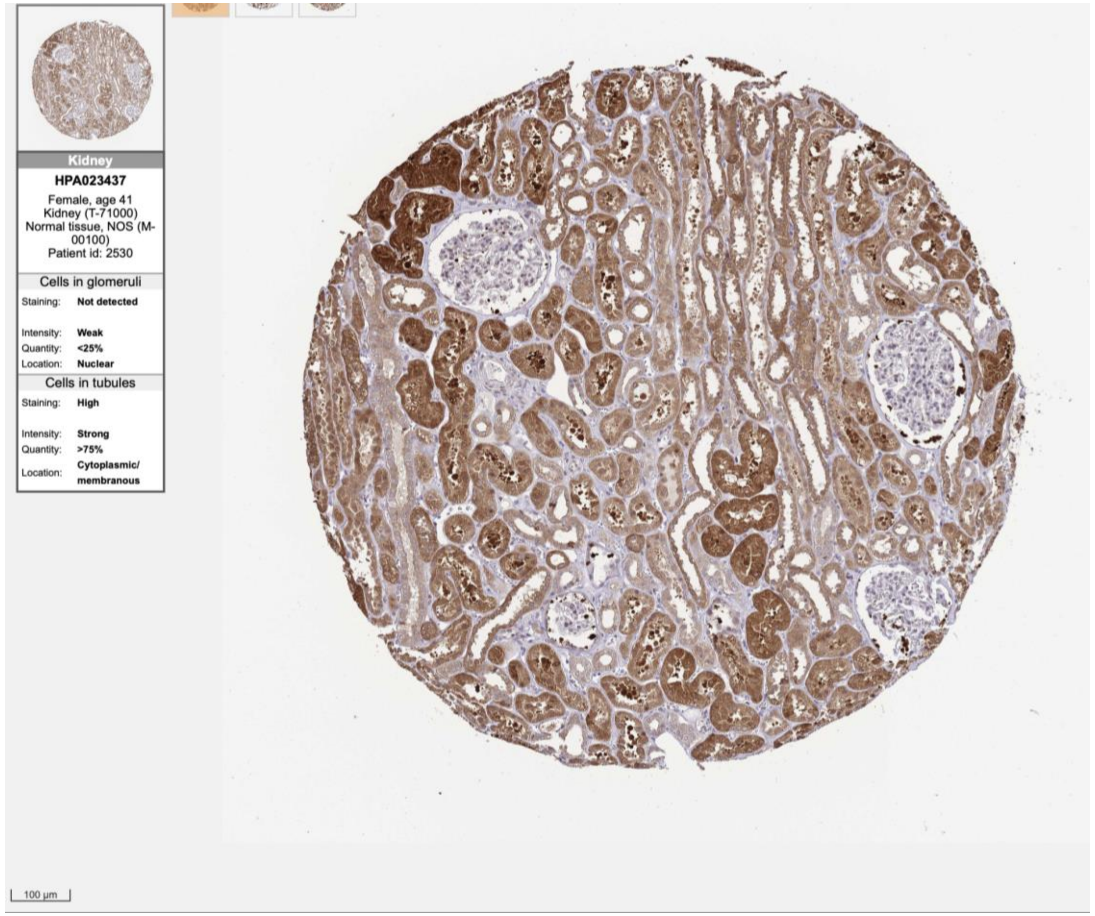
Kidney
HPA023437
Female, age 41
Kidney (T-71000)
Normal tissue, NOS (M-00100)
Patient id: 2530
Cells in glomeruli
Staining: Not detected
Intensity: Weak
Quantity: <25%
Location: Nuclear
Cells in tubules
Staining: High
Intensity: Strong
Quantity: >75%
Location: Cytoplasmic/ membranous
100 µm

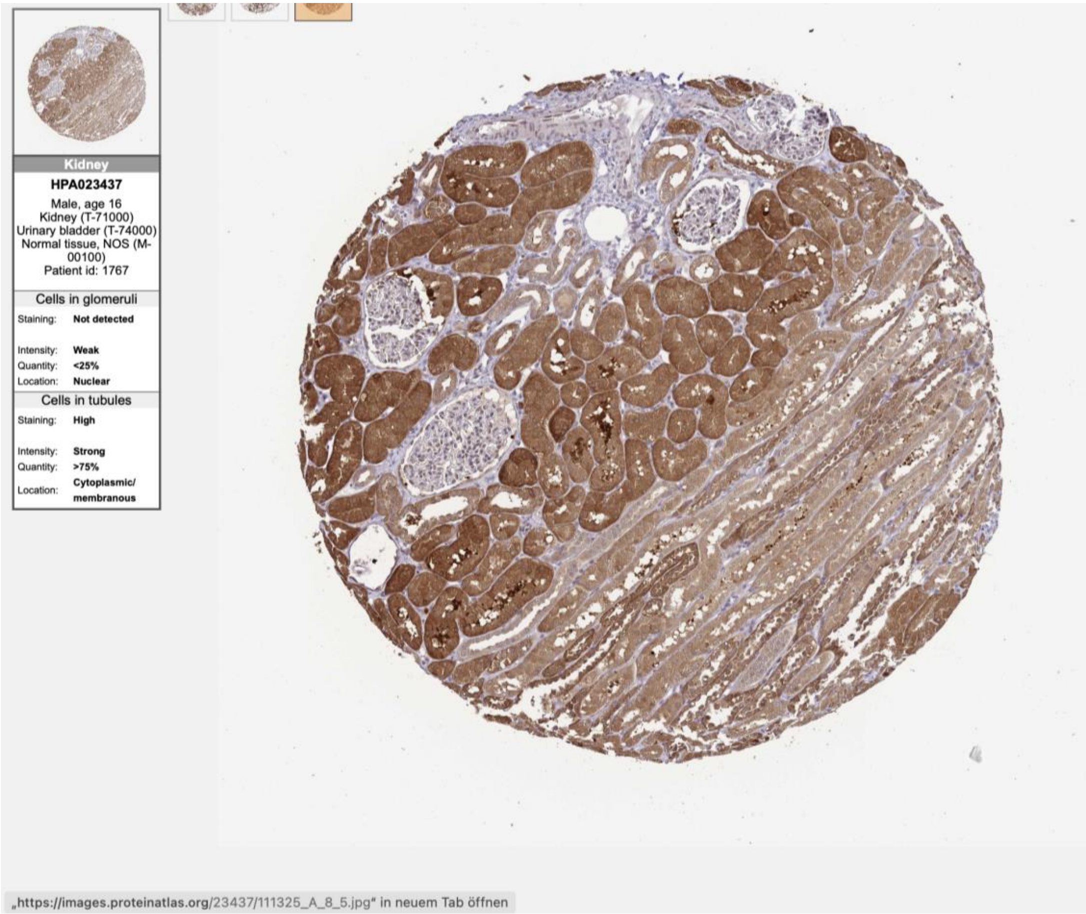
Kidney
HPA023437
Male, age 16
Kidney (T-71000)
Urinary bladder (T-74000)
Normal tissue, NOS (M-00100)
Patient id: 1767
Cells in glomeruli
Staining: Not detected
Intensity: Weak
Quantity: <25%
Location: Nuclear
Cells in tubules
Staining: High
Intensity: Strong
Quantity: >75%
Location: Cytoplasmic/ membranous
„https://images.proteinatlas.org/23437/111325_A_8_5.jpg" in neuem Tab öffnen

ZNF669

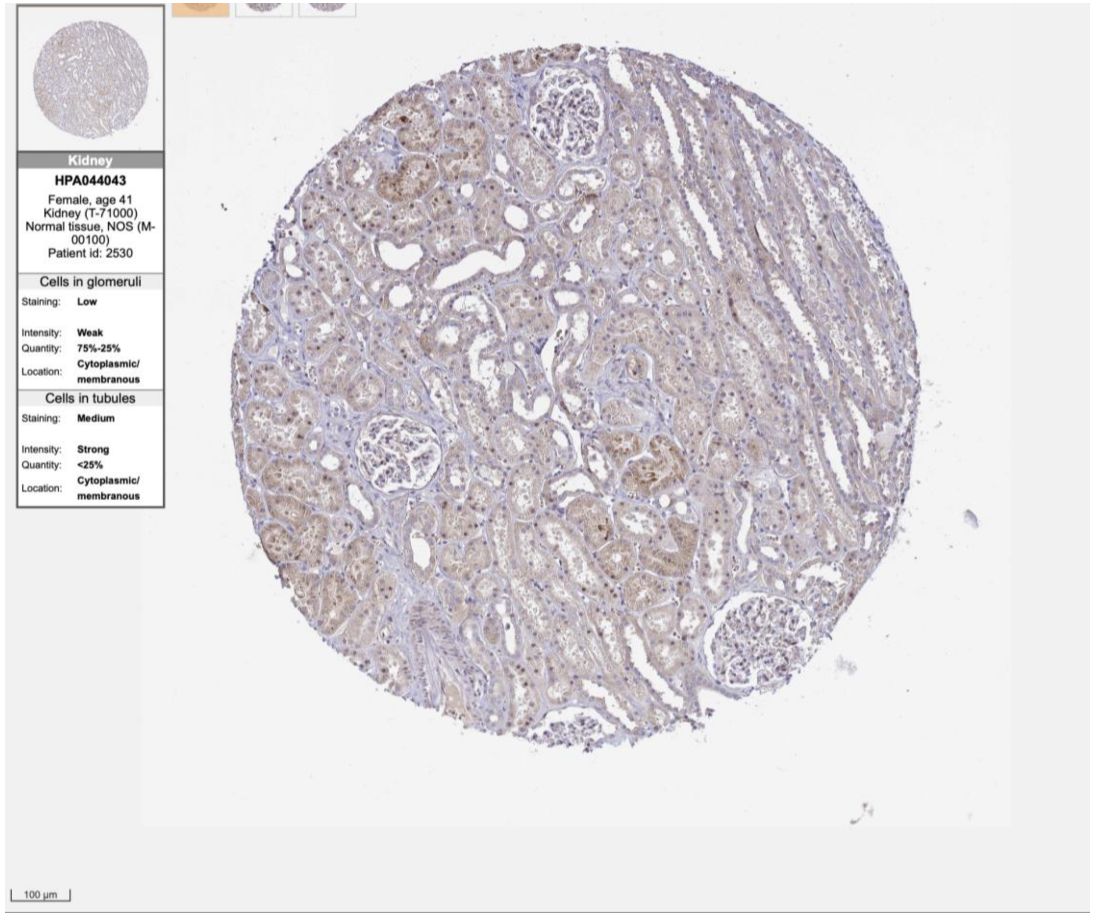
Kidney
HPA044043
Female, age 41
Kidney (T-71000)
Normal tissue, NOS (M-00100)
Patient id: 2530
Cells in glomeruli
Staining: Low
Intensity: Weak
Quantity: 75%-25%
Location: Cytoplasmic/ membranous
Cells in tubules
Staining: Medium
Intensity: Strong
Quantity: <25%
Location: Cytoplasmic/ membranous
100 µm

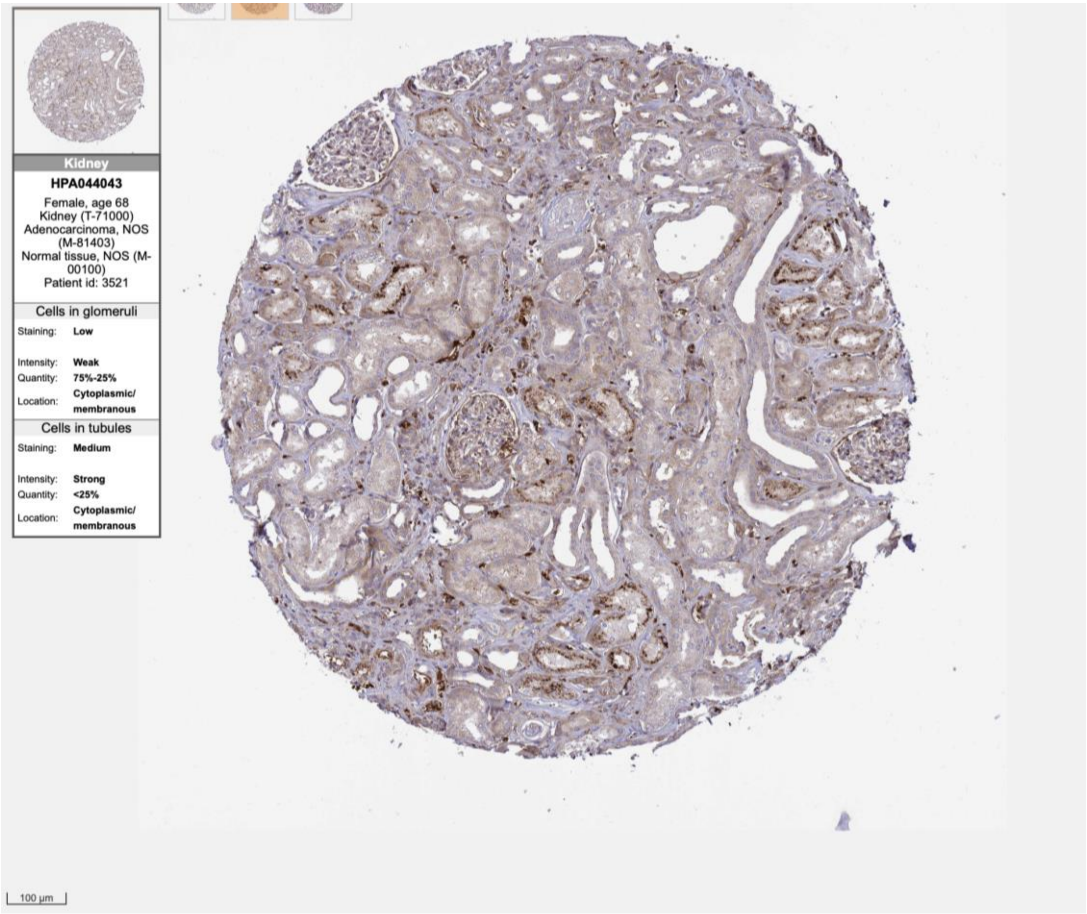
Kidney
HPA044043
Female, age 68
Kidney (T-71000)
Adenocarcinoma, NOS (M-81403)
Normal tissue, NOS (M-00100)
Patient id: 3521
Cells in glomeruli
Staining: Low
Intensity: Weak
Quantity: 75%-25%
Location: Cytoplasmic/ membranous
Cells in tubules
Staining: Medium
Intensity: Strong
Quantity: <25%
Location: Cytoplasmic/ membranous
100 µm

ZNF682

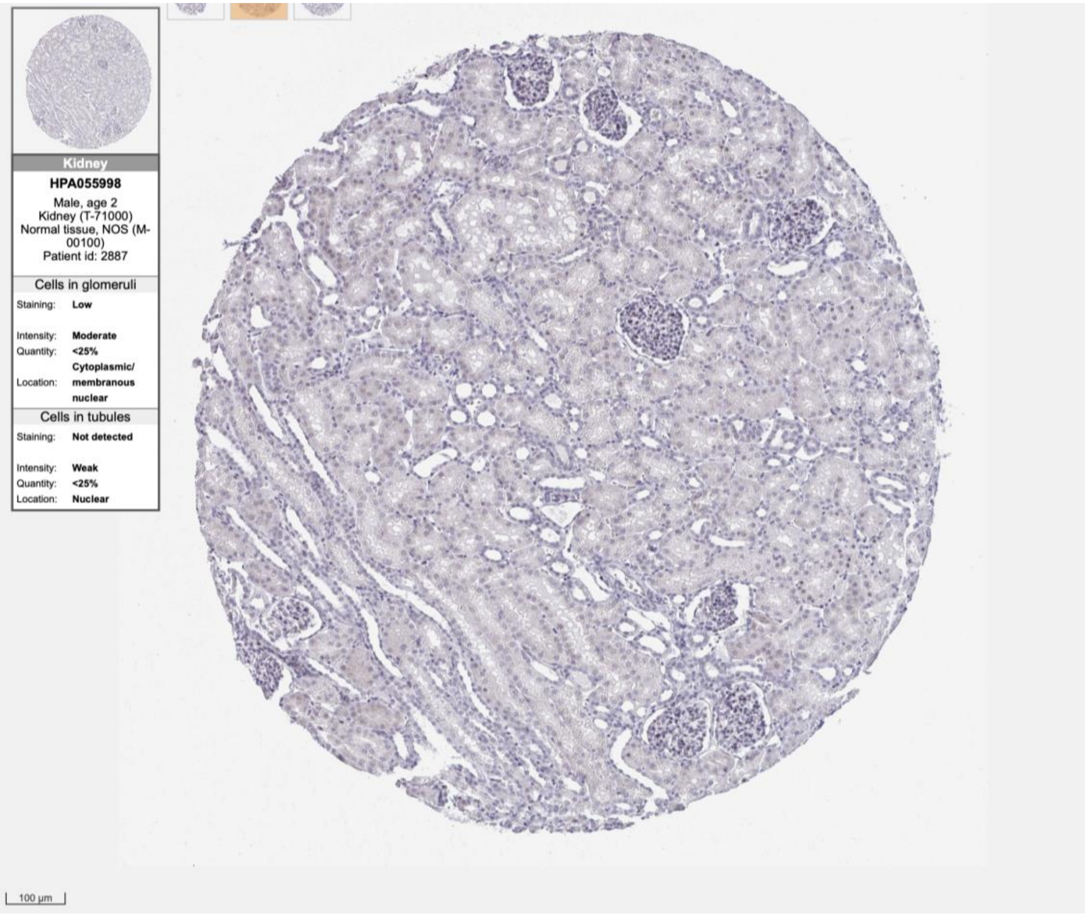
Kidney
HPA055998
Male, age 2
Kidney (T-71000)
Normal tissue, NOS (M-00100)
Patient id: 2887
Cells in glomeruli
Staining: Low
Intensity: Moderate
Quantity: <25%
Location: Cytoplasmic/ membranous nuclear
Cells in tubules
Staining: Not detected
Intensity: Weak
Quantity: <25%
Location: Nuclear
100 µm

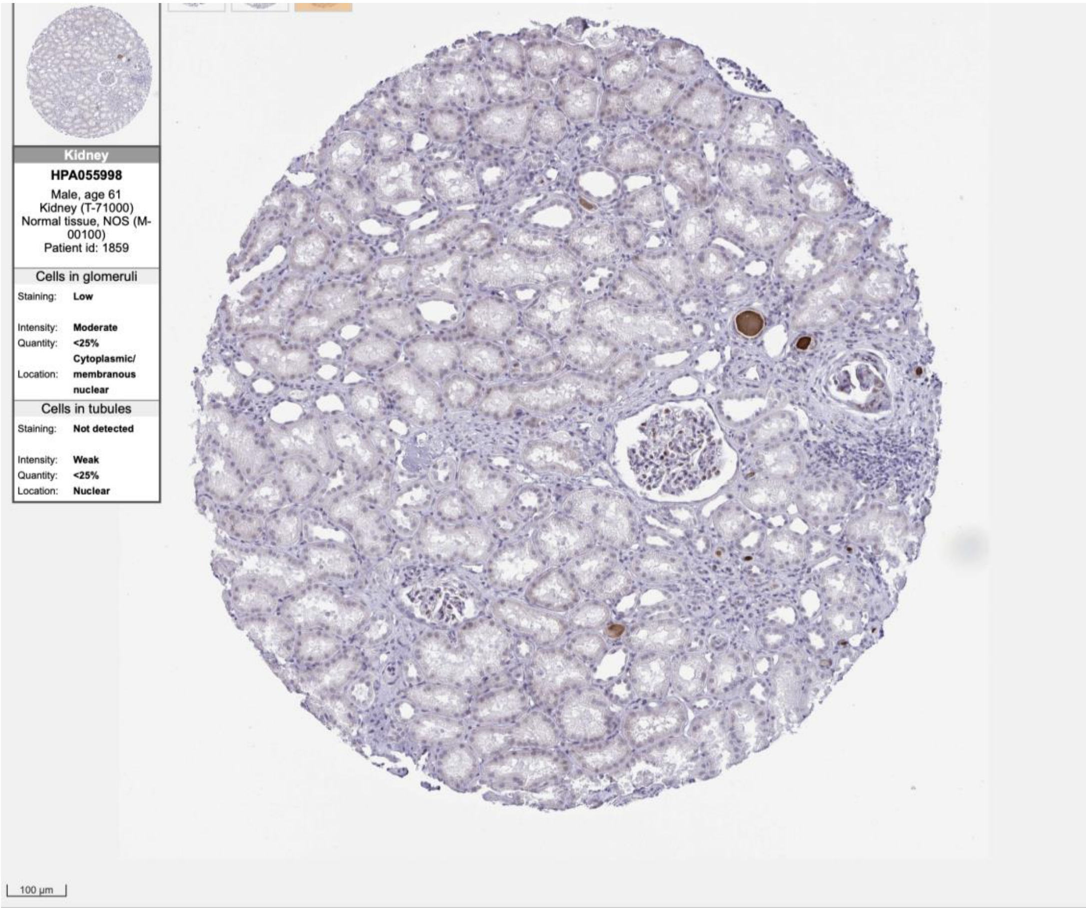
Kidney
HPA055998
Male, age 61
Kidney (T-71000)
Normal tissue, NOS (M-00100)
Patient id: 1859
Cells in glomeruli
Staining: Low
Intensity: Moderate
Quantity: <25%
Location: Cytoplasmic/ membranous nuclear
Cells in tubules
Staining: Not detected
Intensity: Weak
Quantity: <25%
Location: Nuclear
100 µm

ZNF772

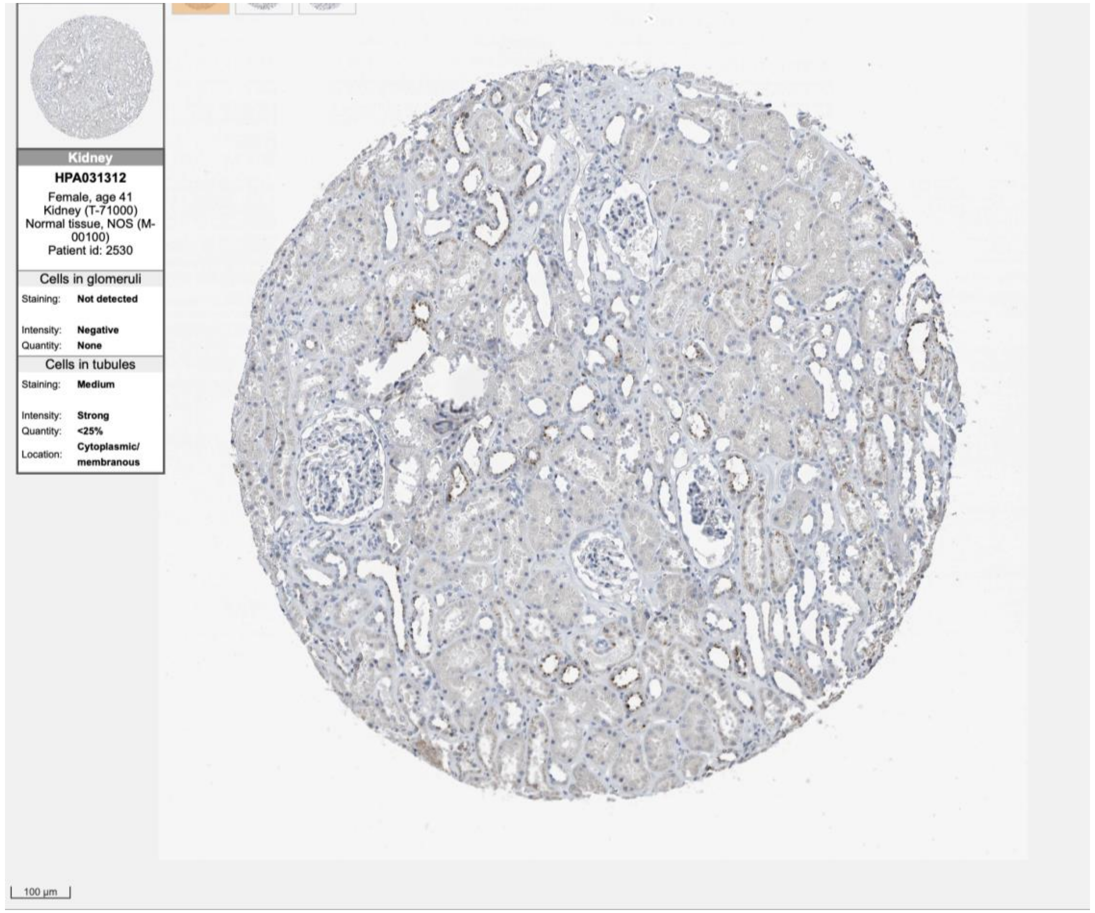
Kidney
HPA031312
Female, age 41
Kidney (T-71000)
Normal tissue, NOS (M-00100)
Patient id: 2530
Cells in glomeruli
Staining: Not detected
Intensity: Negative
Quantity: None
Cells in tubules
Staining: Medium
Intensity: Strong
Quantity: <25%
Location: Cytoplasmic/ membranous
100 µm

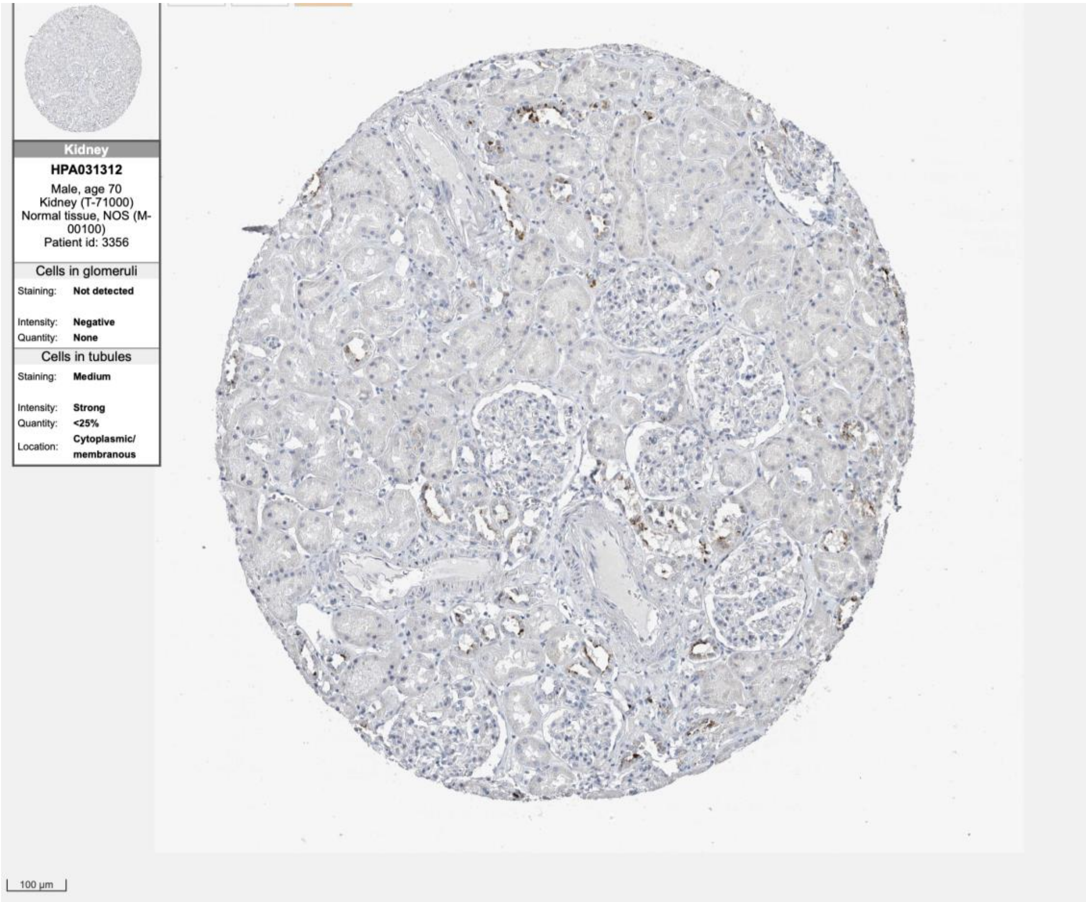
Kidney
HPA031312
Male, age 70
Kidney (T-71000)
Normal tissue, NOS (M-00100)
Patient id: 3356
Cells in glomeruli
Staining: Not detected
Intensity: Negative
Quantity: None
Cells in tubules
Staining: Medium
Intensity: Strong
Quantity: <25%
Location: Cytoplasmic/ membranous
100 µm

SOX2

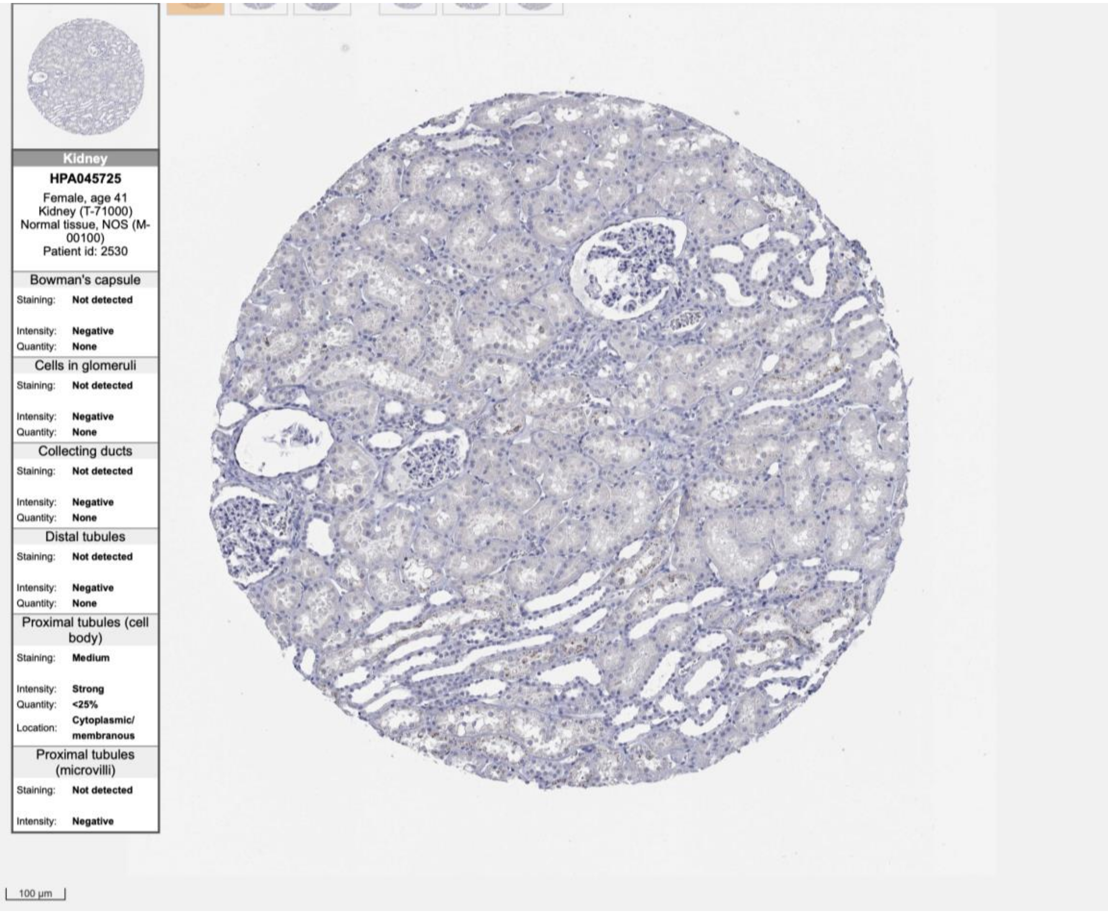
Kidney
HPA045725
Female, age 41
Kidney (T-71000)
Normal tissue, NOS (M-00100)
Patient id: 2530
Bowman's capsule
Staining: Not detected
Intensity: Negative
Quantity: None
Cells in glomeruli
Staining: Not detected
Intensity: Negative
Quantity: None
Collecting ducts
Staining: Not detected
Intensity: Negative
Quantity: None
Distal tubules
Staining: Not detected
Intensity: Negative
Quantity: None
Proximal tubules (cell body)
Staining: Medium
Intensity: Strong
Quantity: <25%
Location: Cytoplasmic/ membranous
Proximal tubules (microvilli)
Staining: Not detected
Intensity: Negative
100 µm

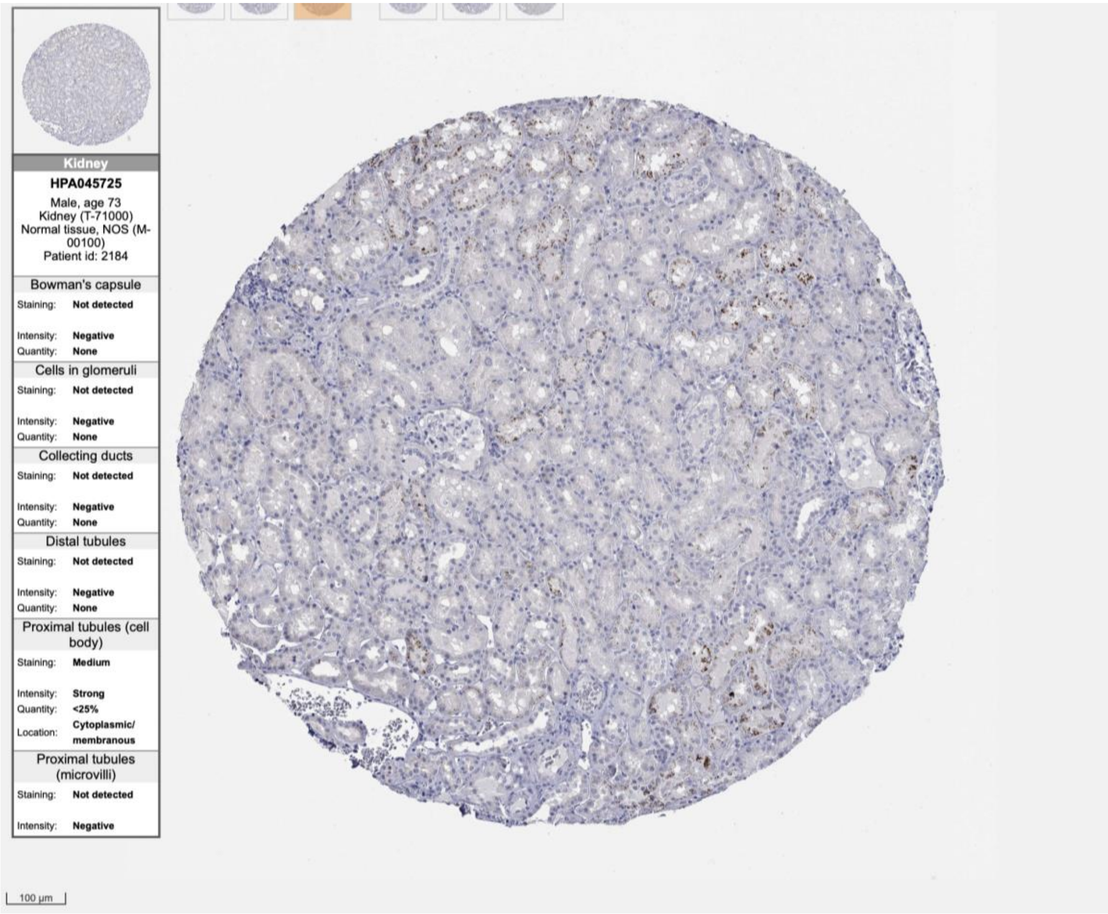
Kidney
HPA045725
Male, age 73
Kidney (T-71000)
Normal tissue, NOS (M-00100)
Patient id: 2184
Bowman's capsule
Staining: Not detected
Intensity: Negative
Quantity: None
Cells in glomeruli
Staining: Not detected
Intensity: Negative
Quantity: None
Collecting ducts
Staining: Not detected
Intensity: Negative
Quantity: None
Distal tubules
Staining: Not detected
Intensity: Negative
Quantity: None
Proximal tubules (cell body)
Staining: Medium
Intensity: Strong
Quantity: <25%
Location: Cytoplasmic/ membranous
Proximal tubules (microvilli)
Staining: Not detected
Intensity: Negative
100 µm

ZNF367

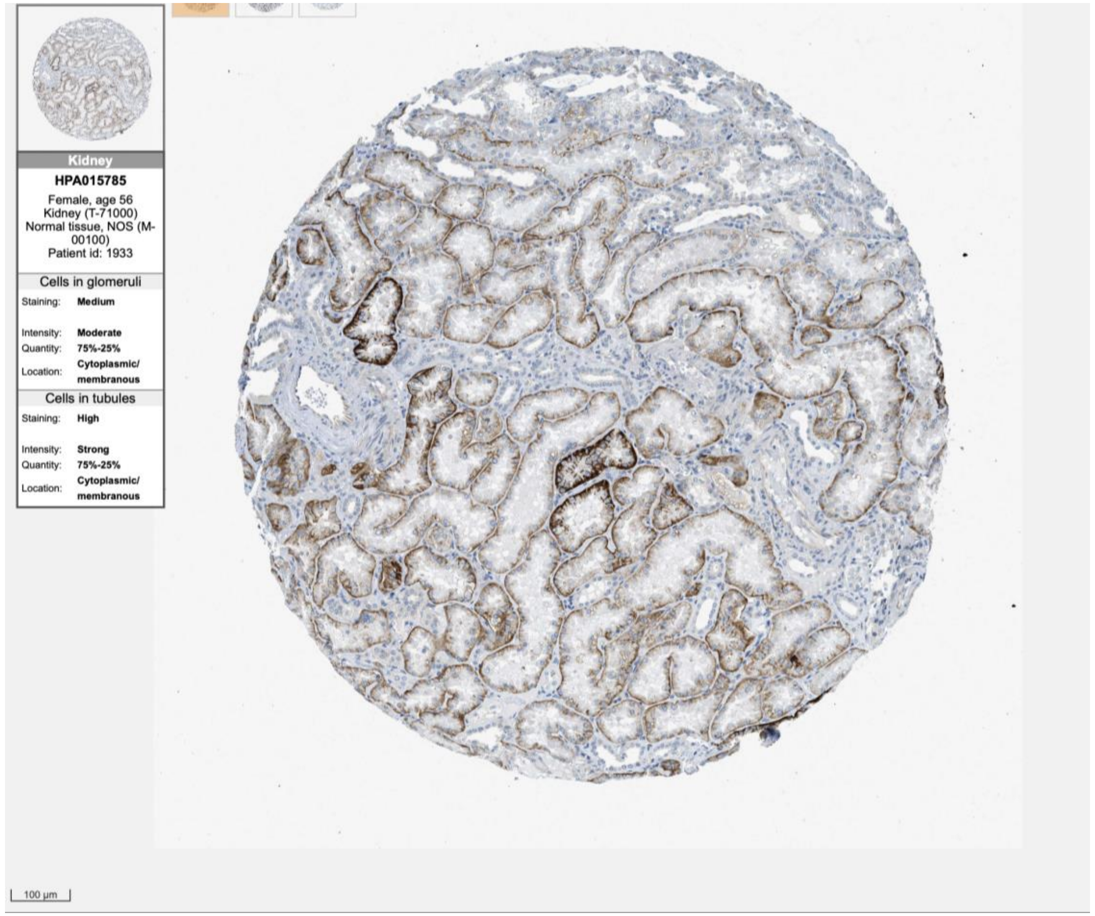
Kidney
HPA015785
Female, age 56
Kidney (T-71000)
Normal tissue, NOS (M-00100)
Patient id: 1933
Cells in glomeruli
Staining: Medium
Intensity: Moderate
Quantity: 75%-25%
Location: Cytoplasmic/membranous
Cells in tubules
Staining: High
Intensity: Strong
Quantity: 75%-25%
Location: Cytoplasmic/membranous
100 µm

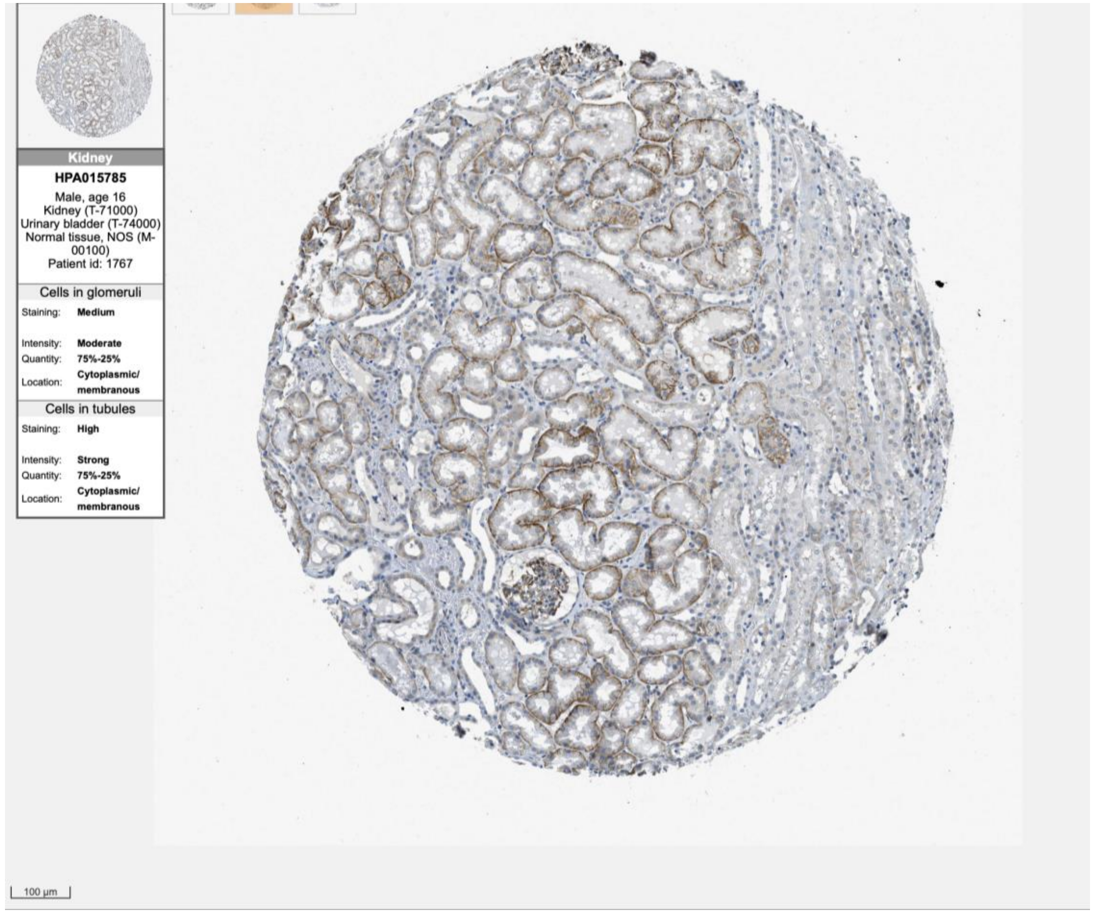
Kidney
HPA015785
Male, age 16
Kidney (T-71000)
Urinary bladder (T-74000)
Normal tissue, NOS (M-00100)
Patient id: 1767
Cells in glomeruli
Staining: Medium
Intensity: Moderate
Quantity: 75%-25%
Location: Cytoplasmic/membranous
Cells in tubules
Staining: High
Intensity: Strong
Quantity: 75%-25%
Location: Cytoplasmic/membranous
100 µm
