# Supplementary material for: Evidence for conserved expression of genes annotated as associated with brain-related biological processes in human podocytes and brain
Source: BMC Nephrol. 2026 Mar 4;27:230. doi: 10.1186/s12882-026-04877-2 (PMC13067571; doi:10.1186/s12882-026-04877-2)
Supplement: Supplementary file 15 — Supplementary Material 15: Figure S9 (figureS9.pdf): SIX2-positive UdRPCs differentiated into human podocytes. Immunofluorescence-based detection revealed that all four urine-derived podocytes and a reference immortalized podocyte cell line (AB8/13) express the podocyte-associated markers. (A) Nephrin (NPHS1). (B) α−Actinin 4 (ACTN4). Scale bars: 100 μm. [file 12882_2026_4877_MOESM15_ESM.pdf]

A

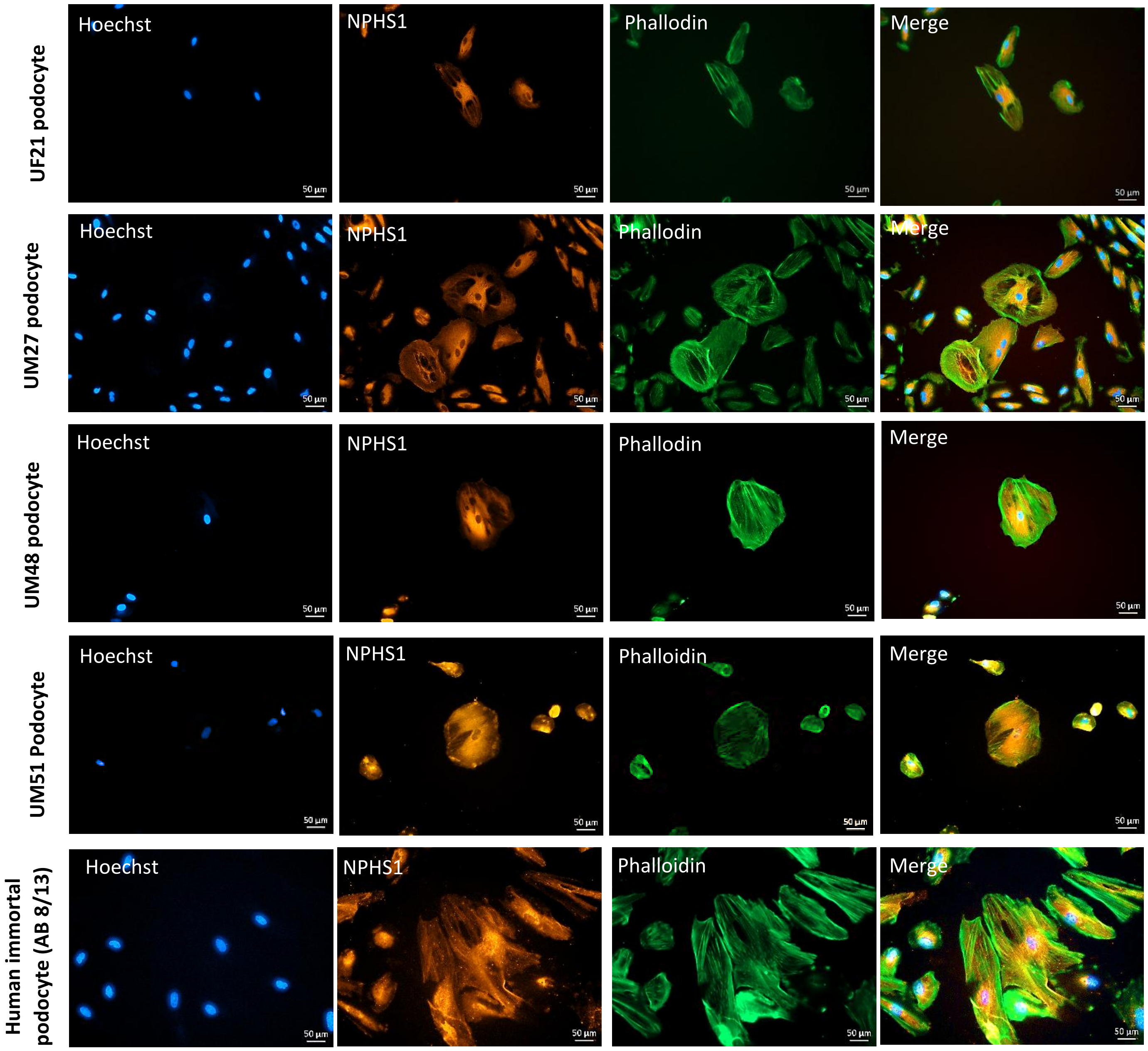

UF21 podocyte
UM27 podocyte
UM48 podocyte
UM51 Podocyte
Human immortal podocyte (AB 8/13)
Hoechst
NPHS1
Phallodin
Phalloidin
Merge
50 μm

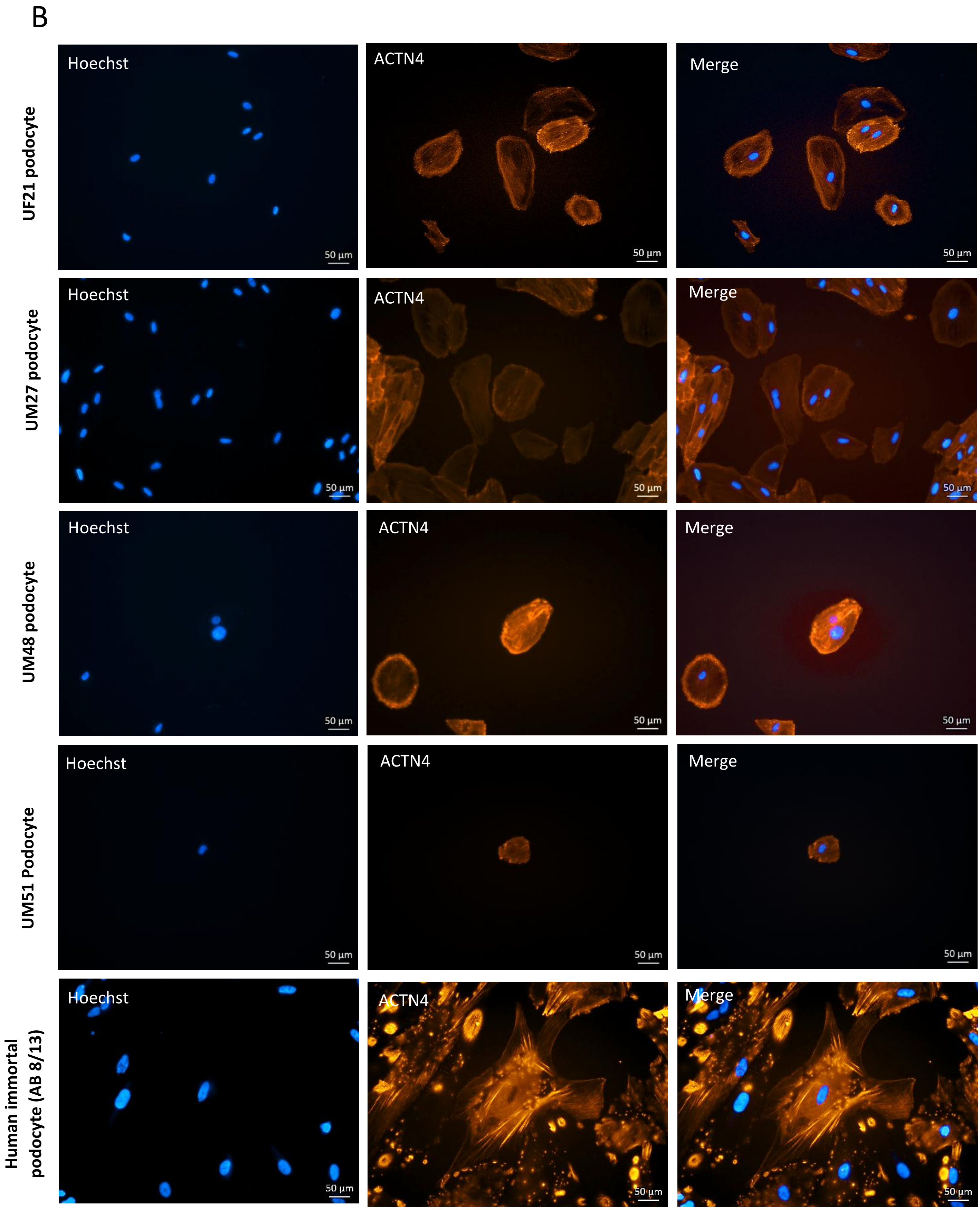
B
UF21 podocyte
UM27 podocyte
UM48 podocyte
UM51 Podocyte
Human immortal podocyte (AB 8/13)
Hoechst
ACTN4
Merge
50 µm

Figure S9 (figureS9.pdf): SIX2-positive UdRPCs differentiated into human podocytes.
Immunofluorescence-based detection revealed that all four urine-derived podocytes and a reference immortalized podocyte cell line (AB8/13) express the podocyte-associated markers

(A) Nephrin (NPHS1)

(B) α–Actinin 4 (ACTN4). Scale bars: 100 µm.
