# Supplementary material for: Evidence for conserved expression of genes annotated as associated with brain-related biological processes in human podocytes and brain
Source: BMC Nephrol. 2026 Mar 4;27:230. doi: 10.1186/s12882-026-04877-2 (PMC13067571; doi:10.1186/s12882-026-04877-2)
Supplement: Supplementary file 16 — Supplementary Material 16: Figure S10 (figureS10.pdf): SIX2-positive UdRPCs are bi-potential. Immunofluorescence-based detection revealed that podocytes express Nephrin (NPHS1) and not the tubular markers- AQP1, CLCNKB and Na+-K+-ATPase as seen in the tubular differentiated cells. Secondary antibody alone was negative. Scale bars: 100 μm. [file 12882_2026_4877_MOESM16_ESM.pdf]

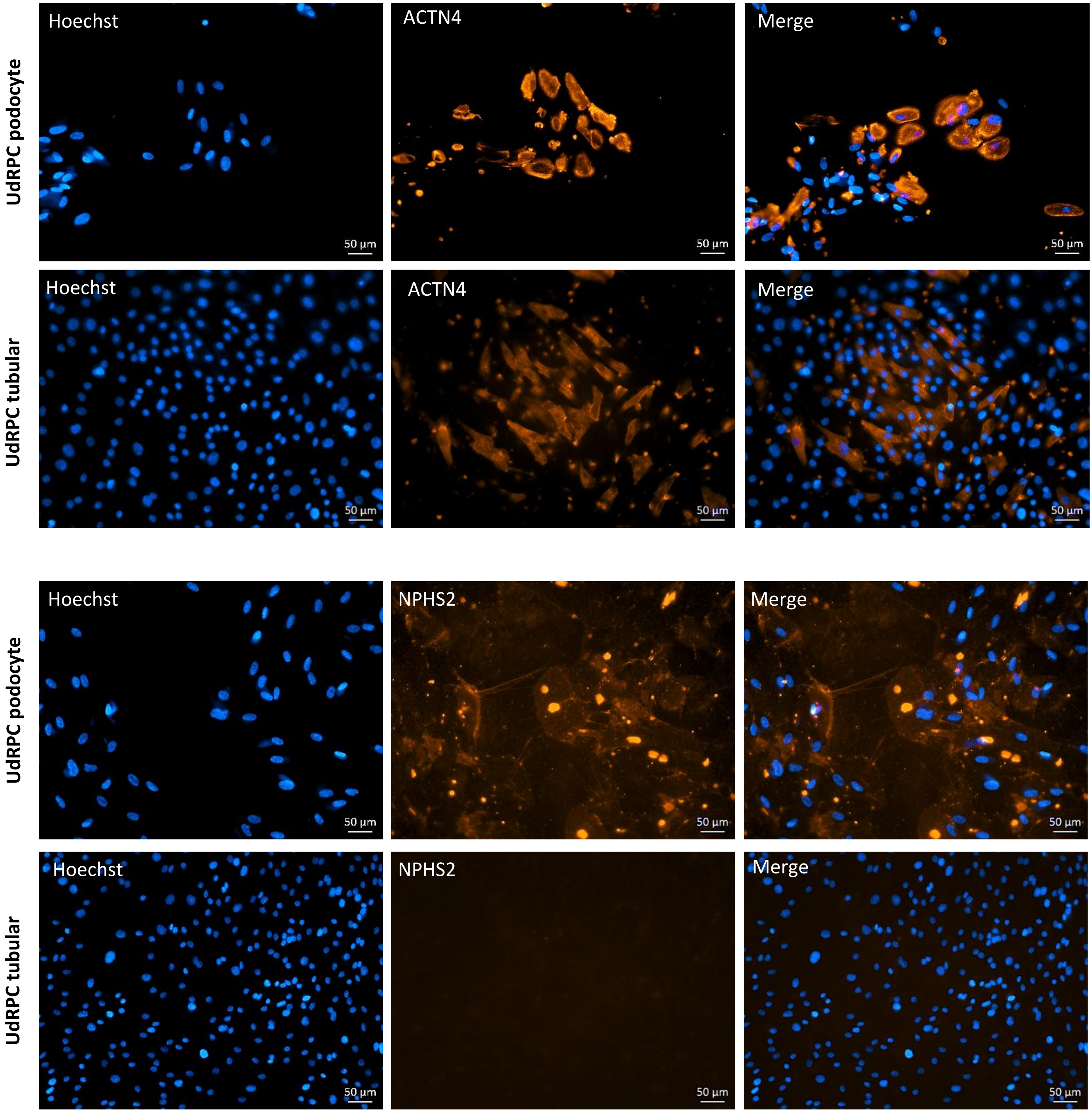
UdRPC podocyte
Hoechst
ACTN4
Merge
UdRPC tubular
Hoechst
ACTN4
Merge
UdRPC podocyte
Hoechst
NPHS2
Merge
UdRPC tubular
Hoechst
NPHS2
Merge
50 µm

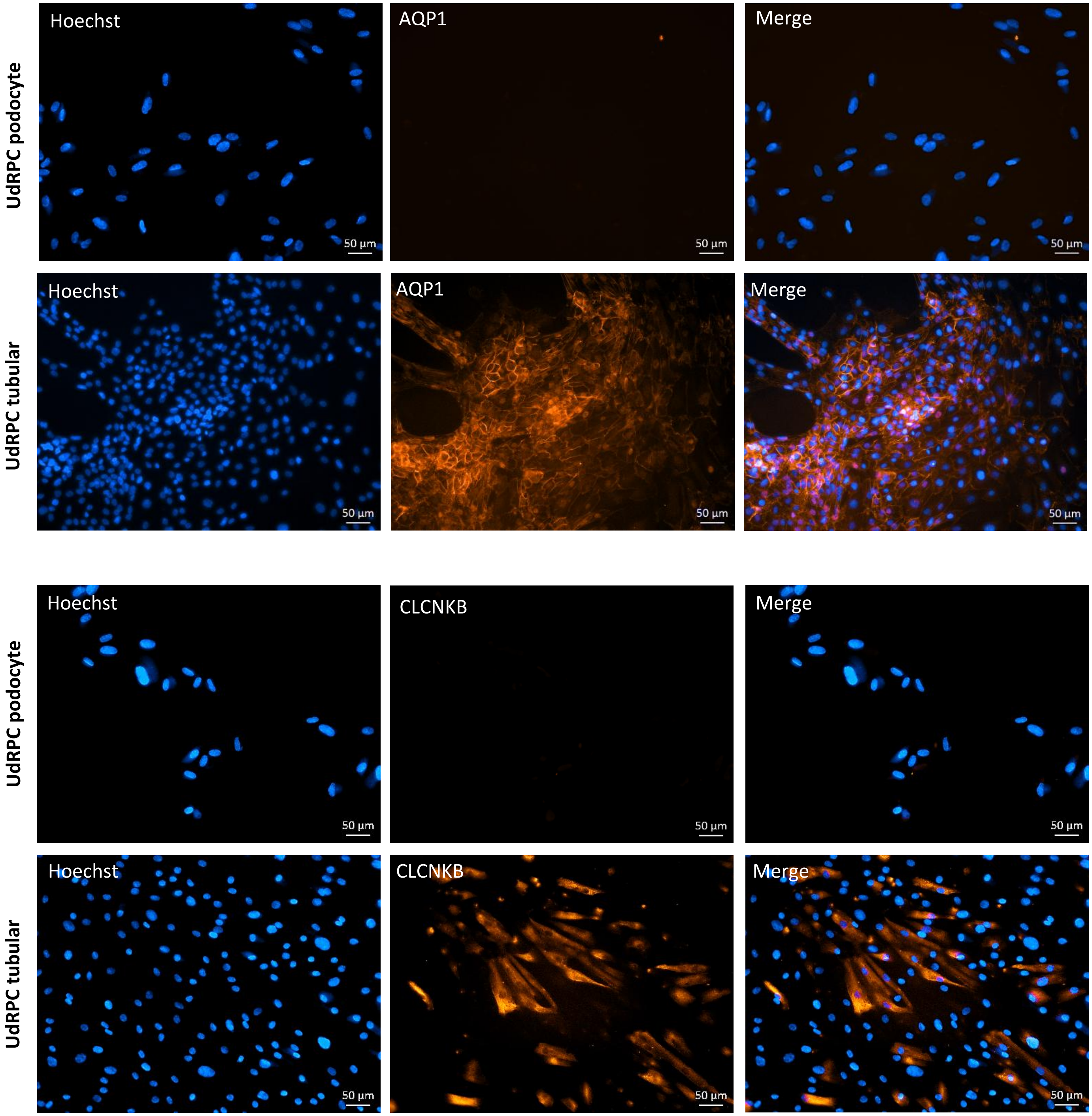
UdRPC podocyte
UdRPC tubular
Hoechst
AQP1
Merge
CLCNKB
50 µm

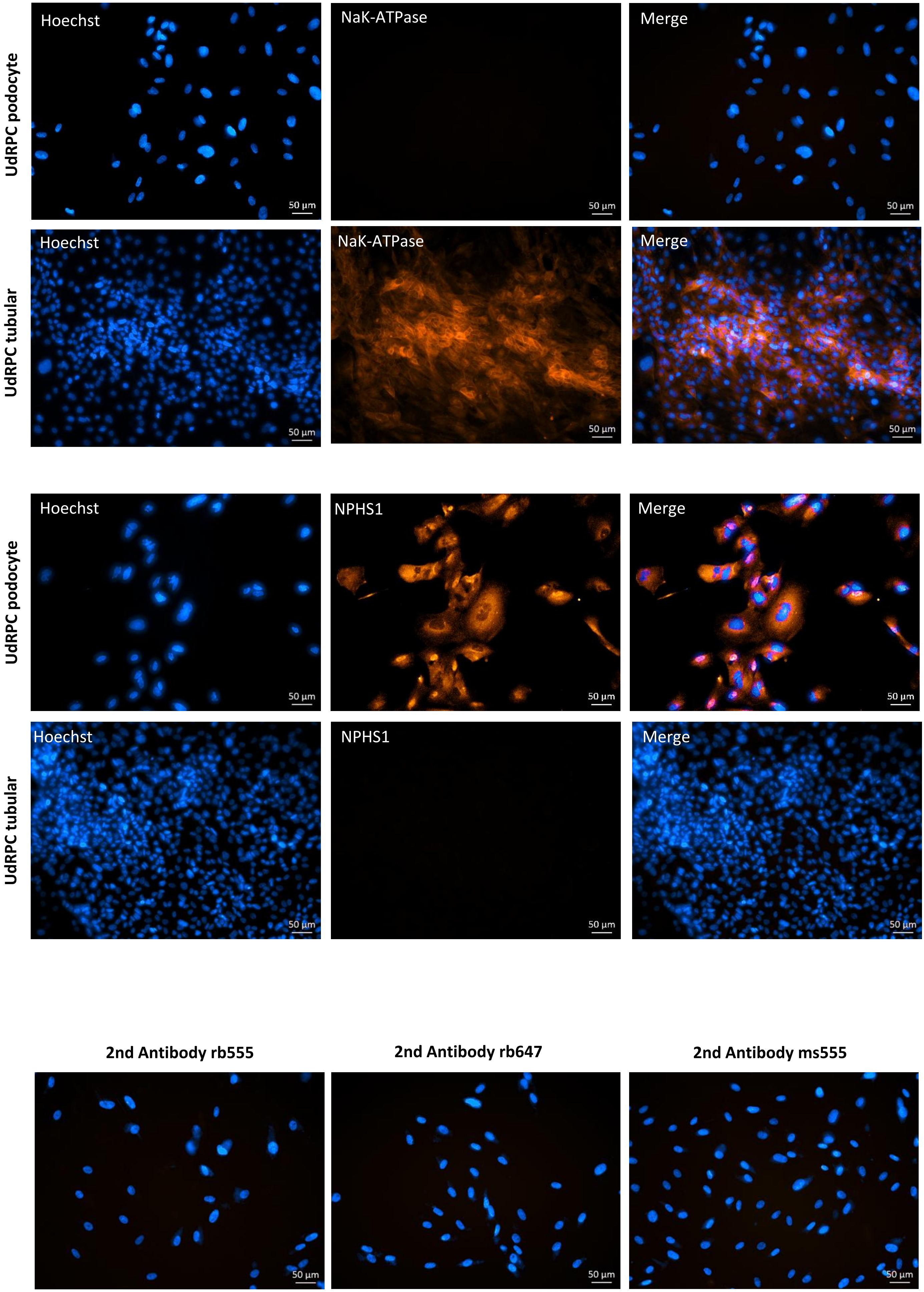
UdRPC podocyte
Hoechst
NaK-ATPase
Merge
50 µm
UdRPC tubular
Hoechst
NaK-ATPase
Merge
UdRPC podocyte
Hoechst
NPHS1
Merge
UdRPC tubular
Hoechst
NPHS1
Merge
2nd Antibody rb555
2nd Antibody rb647
2nd Antibody ms555

Figure S10 (figureS10.pdf): SIX2-positive UdRPCs are bi-potential. Immunofluorescence-based detection revealed that podocytes express Nephrin (NPHS1) and not the tubular markers- AQP1, CLCNKB and Na+-K+-ATPase as seen in the tubular differentiated cells. Secondary antibody alone was negative. Scale bars: 100 µm.
