# Supplementary material for: Evidence for conserved expression of genes annotated as associated with brain-related biological processes in human podocytes and brain
Source: BMC Nephrol. 2026 Mar 4;27:230. doi: 10.1186/s12882-026-04877-2 (PMC13067571; doi:10.1186/s12882-026-04877-2)
Supplement: Supplementary file 17 — Supplementary Material 17: Figure S11: Complete images of Western Blot analyses of brain-associated proteins expressed in human podocytes (figS11_WB_uncropped.pdf). [file 12882_2026_4877_MOESM17_ESM.pdf]

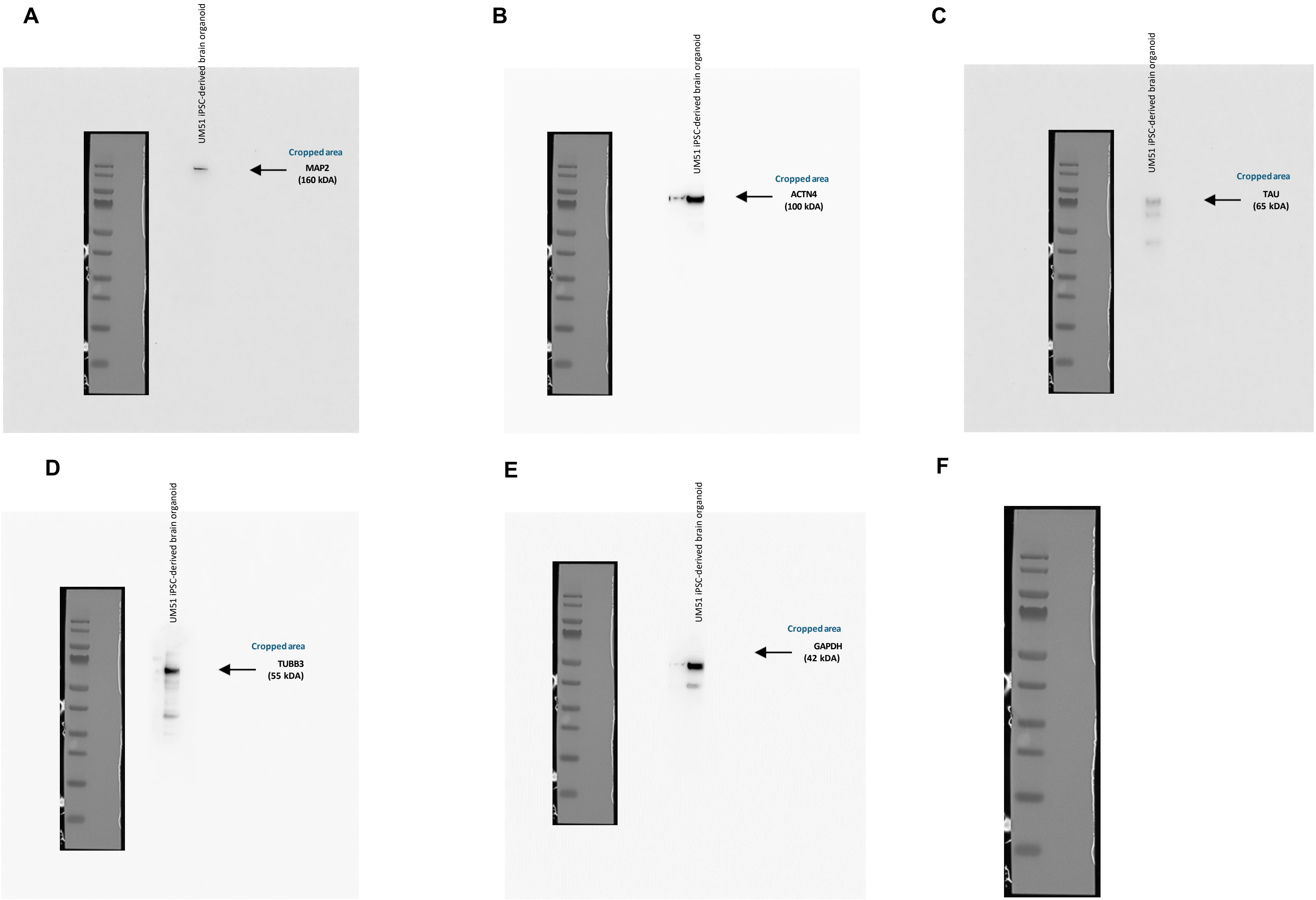
A
UM51 iPSC-derived brain organoid
Cropped area
MAP2
(160 kDA)
B
UM51 iPSC-derived brain organoid
Cropped area
ACTN4
(100 kDA)
C
UM51 iPSC-derived brain organoid
Cropped area
TAU
(65 kDA)
D
UM51 iPSC-derived brain organoid
Cropped area
TUBB3
(55 kDA)
E
UM51 iPSC-derived brain organoid
Cropped area
GAPDH
(42 kDA)
F

Supplementary Figure S11: Uncropped western Blot images UM51-iPSC derived cortical brain organoid

The detected proteins are presented from left to right. A: MAP2, B: ACTN4, C: TAU, D: TUBB3, E:GAPDH. Supplementary figure F represents the ladder for the western blot images. The loading scheme starting next to the ladder from the left side as follows: UM51-iPSC derived cortical brain organoid. The uncropped western blot images belong to Figure 6A.

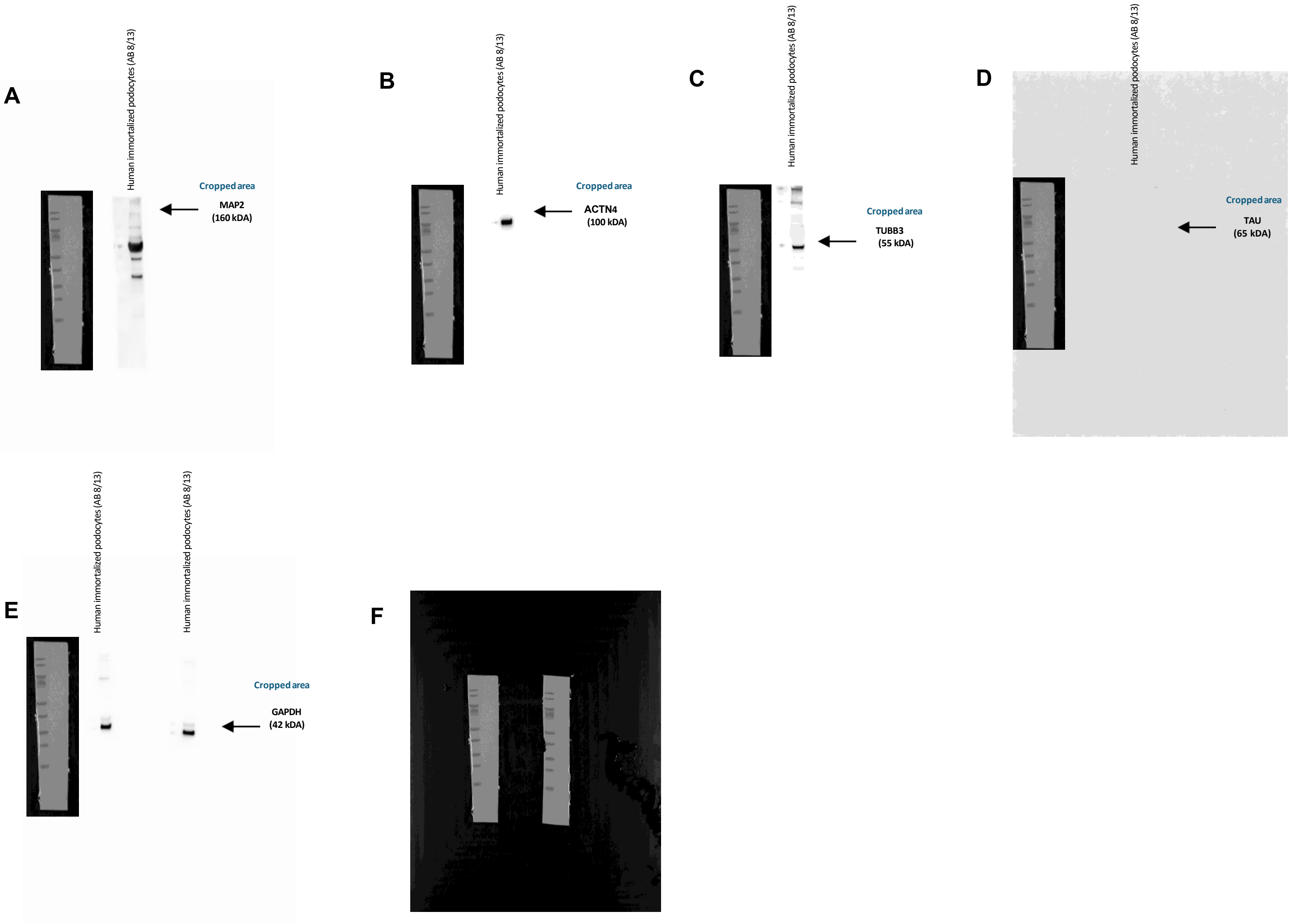
A
Human immortalized podocytes (AB 8/13)
Cropped area
MAP2
(160 kDA)
B
Human immortalized podocytes (AB 8/13)
Cropped area
ACTN4
(100 kDA)
C
Human immortalized podocytes (AB 8/13)
Cropped area
TUBB3
(55 kDA)
D
Human immortalized podocytes (AB 8/13)
Cropped area
TAU
(65 kDA)
E
Human immortalized podocytes (AB 8/13)
Human immortalized podocytes (AB 8/13)
Cropped area
GAPDH
(42 kDA)
F

Supplementary Figure S11: Uncropped western Blot images Human immortalized podocytes (AB 8/13).

The detected proteins are presented from left to right. A: MAP2, B: : ACTN4, C: TUBB3, D: TAU, E: GAPDH. Supplementary figure F represents the ladder for the western blot images. The loading scheme starting next to the ladder from the left side as follows: Human immortalized podocytes (AB 8/13). The uncropped western blot images belong to Figure 6B.

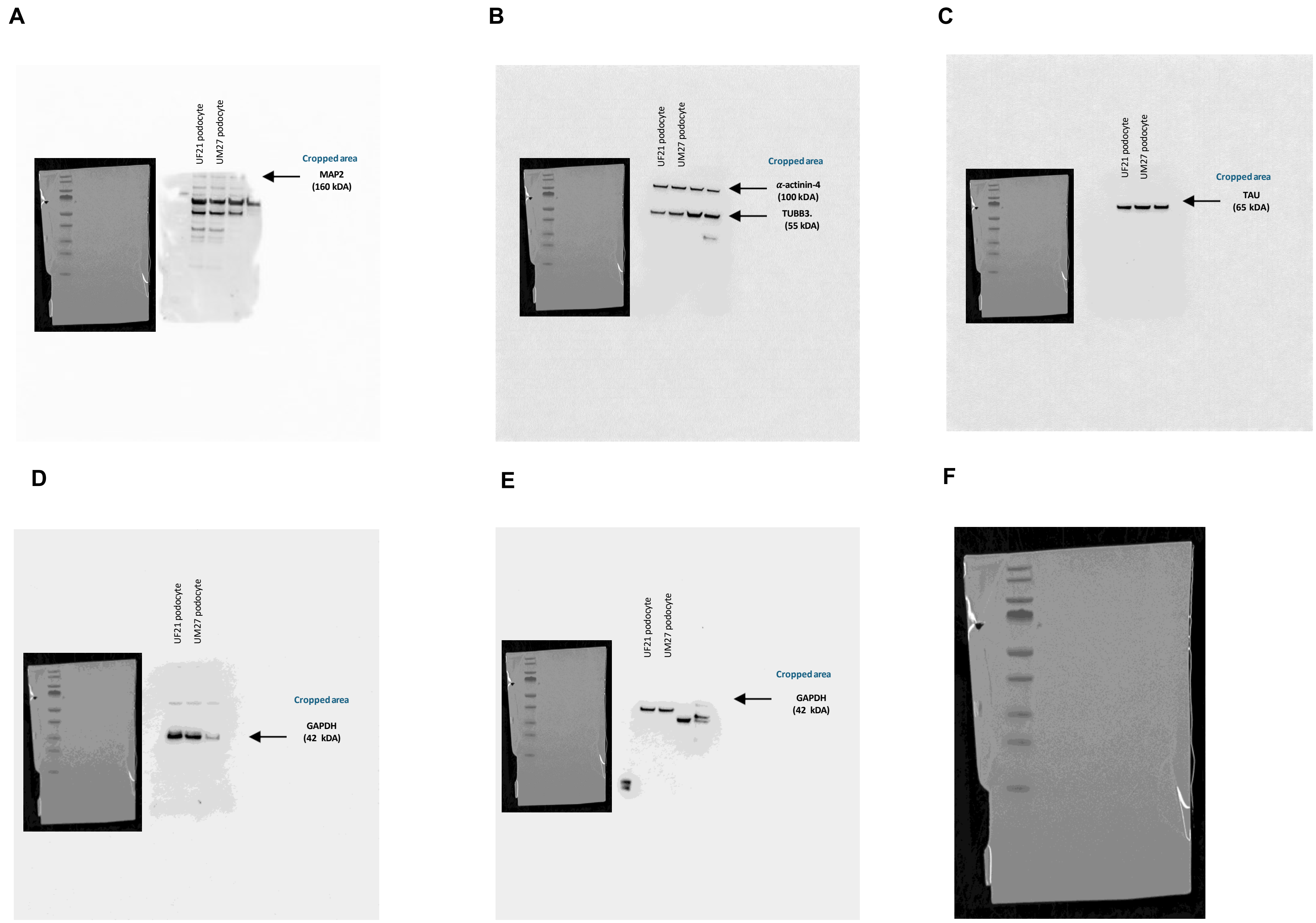
A
UF21 podocyte
UM27 podocyte
Cropped area
MAP2
(160 kDA)
B
UF21 podocyte
UM27 podocyte
Cropped area
α-actinin-4
(100 kDA)
TUBB3.
(55 kDA)
C
UF21 podocyte
UM27 podocyte
Cropped area
TAU
(65 kDA)
D
UF21 podocyte
UM27 podocyte
Cropped area
GAPDH
(42 kDA)
E
UF21 podocyte
UM27 podocyte
Cropped area
GAPDH
(42 kDA)
F

Supplementary Figure S11: Uncropped western Blot images UF21/UM27.

The detected proteins are presented from left to right. A: MAP2, B: ACTN4 + TUBB3, C: TAU, D+E: GAPDH. Supplementary figure F represents the ladder for the western blot images. The loading scheme starting next to the ladder from the left side as follows: UF21 podocyte and UM27 podocytes. The uncropped western blot images belong to Figure 6C.

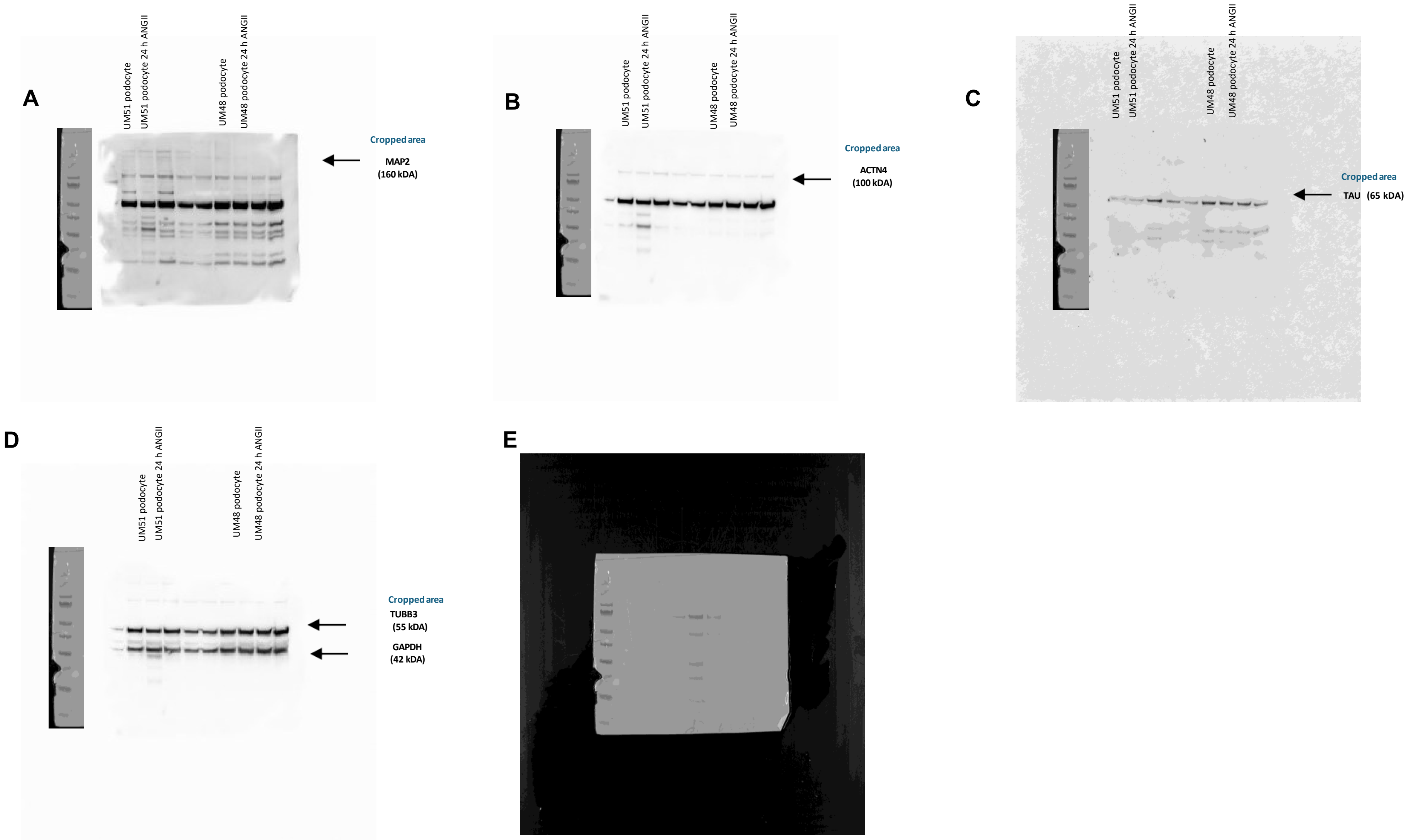
A
UM51 podocyte
UM51 podocyte 24 h ANGII
UM48 podocyte
UM48 podocyte 24 h ANGII
Cropped area
MAP2
(160 kDA)
B
UM51 podocyte
UM51 podocyte 24 h ANGII
UM48 podocyte
UM48 podocyte 24 h ANGII
Cropped area
ACTN4
(100 kDA)
C
UM51 podocyte
UM51 podocyte 24 h ANGII
UM48 podocyte
UM48 podocyte 24 h ANGII
Cropped area
TAU (65 kDA)
D
UM51 podocyte
UM51 podocyte 24 h ANGII
UM48 podocyte
UM48 podocyte 24 h ANGII
Cropped area
TUBB3
(55 kDA)
GAPDH
(42 kDA)
E

Supplementary Figure S11: Uncropped western Blot images UM48/UM51 podocytes.

The detected proteins are presented from left to right. A: MAP2, B: ACTN4, C: TAU, D: TUBB3+GAPDH. Supplementary figure E represents the ladder for the western blot images. The loading scheme starting next to the ladder from the left side as follows: UM51 podocytes, UM51 podocytes 24 h ANGII, three rows samples out of context, UM48 podocytes, UM48 podocytes 24 h ANGII, two rows samples out of context. The uncropped western blot images belong to Figure 6D.
